# Long-read sequencing reveals pre-meiotic gene conversion in sperm

**DOI:** 10.1038/s41586-026-10901-0

**Published:** 2026-08-26

**Authors:** Regev Schweiger, Sangjin Lee, Chenxi Zhou, Tsun-Po Yang, Stacy Li, Rashesh Sanghvi, Matthew Neville, Katie Smith, Kirsty Roberts, Ayrun Nessa, Sam Wadge, Kerrin S. Small, Peter J. Campbell, Kristian Almstrup, Peter H. Sudmant, Raheleh Rahbari, Richard Durbin

**Affiliations:** 1 Department of Human Genetics and Computational Medicine, Gray School of Medical Sciences, Gray Faculty of Medical and Health Sciences, Tel Aviv University, Tel Aviv, Israel; 2 Blavatnik School of Computer Science and AI, Sackler Faculty of Exact Sciences, Tel Aviv University, Tel Aviv, Israel; 3 Department of Genetics, University of Cambridge, Cambridge, UK; 4 Cancer Ageing and Somatic Mutation, Wellcome Sanger Institute, Hinxton, UK; 5 Department of Integrative Biology, University of California, Berkeley, Berkeley, CA USA; 6 Department of Twin Research and Genetic Epidemiology, King’s College London, London, UK; 7 Wellcome–MRC Cambridge Stem Cell Institute, Cambridge Biomedical Campus, Cambridge, UK; 8 Department of Growth and Reproduction, Copenhagen University Hospital–Rigshospitalet, Copenhagen, Denmark; 9 Department of Cellular and Molecular Medicine, Faculty of Health and Medical Sciences, University of Copenhagen, Copenhagen, Denmark; 10 International Center for Research and Research Training in Endocrine Disruption of Male Reproduction and Child Health, University of Copenhagen, Copenhagen, Denmark; 11 Center for Computational Biology, University of California, Berkeley, Berkeley, CA USA

**Keywords:** Genomics, Haplotypes, DNA recombination

## Abstract

Meiotic recombination is a fundamental process that generates genetic diversity by creating new combinations of existing alleles^1^. Whereas crossovers in humans are well characterized^2^, the more frequent non-crossovers that lead to gene conversion remain challenging to study. Here we show that single high-fidelity long sequencing reads from sperm can capture both crossovers and non-crossovers, which enables effectively arbitrary sample sizes for analysis from a single male. We analysed 2,382 candidate non-crossovers in 15 sperm samples from 13 donors, and identified a consistent component with properties distinct from PRDM9-induced recombination. This phenomenon was not associated with meiotic double-strand break sites identified by DMC1 binding, the crossover recombination map or GC-biased gene conversion, but was associated with genomic fragile sites. This component is also seen in paternal non-crossover gene conversions in pedigree data^3^. Applying the same analysis to 12 blood samples^4^, we observed non-crossover gene conversions with similar properties, but very few crossover events. Further, we demonstrate variation between donors for the different types of recombination, even when they share the same PRDM9 genotype. We suggest that a substantial fraction of the non-crossover gene conversion events seen in sperm arise prior to meiosis.

## Abstract (web summary)

Single-molecule long-read sequencing of human sperm reveals variation in recombination across donors and that a substantial fraction of non-crossover gene conversions arises before meiosis, distinct from PRDM9-driven recombination and associated with fragile sites.

## Main

In meiotic recombination, repair of programmed double-strand breaks (DSBs) causes exchange of genetic material between homologous chromosomes. The accurate execution of this process is essential for maintaining genome integrity and for fertility in many organisms^1^. In humans and many other vertebrates, meiotic DSB generation is localized by sequence-specific binding of the zinc-finger PRDM9 protein^5^. A minority of these DSBs resolve into crossovers (COs), resulting in reciprocal exchange of alleles beyond the recombination site, and many more DSBs resolve as non-crossovers (NCOs), accompanied by a unidirectional transfer of genetic information over short intervals (gene conversion). Additionally, complex recombination events, in which multiple haplotype switches cluster in close proximity (up to a few kilobases), have been observed, in much smaller numbers than simple COs and NCOs^2,6,7^.

It is generally believed that germline NCOs, similar to COs, result from meiotic SPO11-induced DSBs^1^, but the fine-scale map of NCOs is known to be different from that of COs^8^, and we know that gene conversion also occurs in somatic tissues^9^. Indeed, recombination is an essential component of DSB repair in non-meiotic cells, which enables use of homologous sequence as a template during repair^10^. Most of our knowledge about meiotic recombination comes from pedigree^3,6,7,11–13^ and population-based statistical^14–20^ approaches, but these yield limited events per individual or rely on simplifying assumptions about demography, mutation rate and selective neutrality. More directly, it is possible to sequence many sperm cells from a single individual, each of which represents an independent meiosis. Early sperm sequencing assays targeted recombination hotspots by PCR and were limited to a few loci, while newer single-cell methods allow genome-wide insights into COs but lack resolution for NCOs^8,21^. Recently, long-read sperm sequencing spanning multiple heterozygous markers has enabled direct visualization of CO events^22–25^, but confident detection of NCOs requires very high accuracy to avoid confounding with sequence errors. One recent study of recombination events using long-read sequencing data from sperm and testis in human and six primates has examined NCO positional properties and tract lengths across species^26^.

Here we use accurate long-read PacBio CCS sequencing of bulk sperm from 13 donors to identify both COs and NCOs genome-wide, revealing a substantial pre-meiotic NCO component that is shared with somatic tissue, individual-level variation and improved characterization of NCO tract lengths.

We obtained 15 sperm samples from 13 donors (aged 24–74 years; Extended Data Table 1, Supplementary Table 1 and Methods), including a pair of monozygotic twins and 2 other individuals sampled at 2 time points. Ten donors were homozygous for *PRDM9* allele PRDM9_A_, with the others heterozygous for PRDM9_A_ with either PRDM9_B_, PRDM9_D_^ (refs. 27,28^) or a novel allele (Supplementary Note 1). We assigned identifiers to the sperm donors based on their PRDM9 genotype. Nine of the samples were from the TwinsUK^29^ project (hereafter TwinsUK samples) and were sequenced in two batches on both a Pacific Biosciences Sequel II and a Revio instrument, and six were from the Sudmant laboratory (hereafter SL samples) and were sequenced on a Revio instrument. Sequence coverage per sperm sample ranged from 17× to 159× (average 78.2×), with average read length 13,160 bp and a total of 267,461,560 reads (Supplementary Table 1).

In addition to the sperm sequence data, we analysed 94,043,625 PacBio circular consensus sequencing (CCS) reads with an average length of 14,479 bp sequenced by the Platinum Pedigree consortium^4^ from blood samples of 12 individuals of a 4-generation pedigree from the Centre d′Etudes du Polymorphisme Humain (CEPH) from Utah (CEU) collection (Extended Data Table 2 and Supplementary Table 2).

## Identifying candidate recombinant reads

**Fig. 1:**
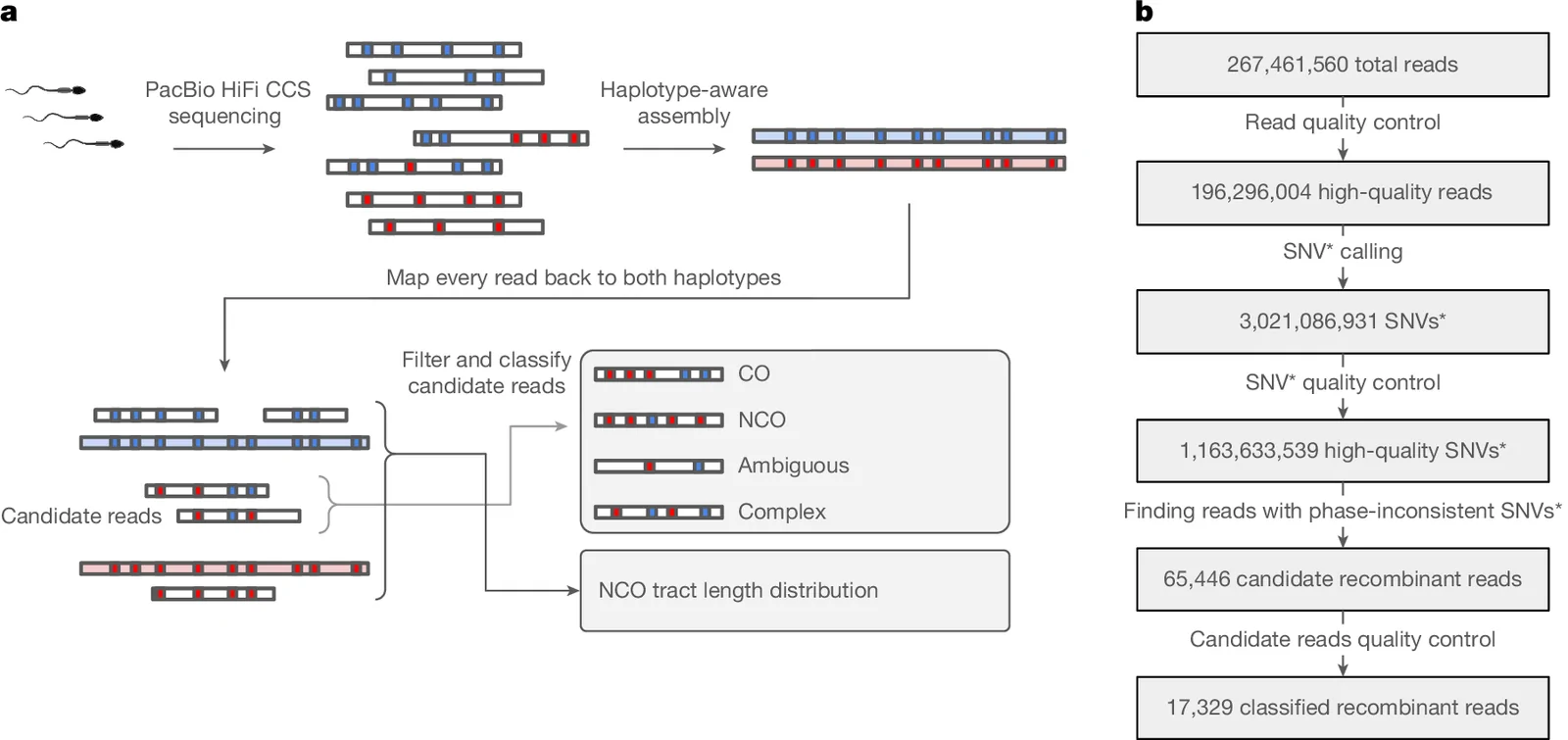
Detecting recombinant reads in sperm samples. **a**, Workflow for data processing. Icons created in BioRender; Rahbari, R. https://BioRender.com/0iaq75j (2026). **b**, Number of reads as the entire dataset passes through the workflow. Asterisks indicate candidate SNVs assigned at read level, and not at sample level.

To ensure accurate read mapping and avoid reference bias we assembled a haplotype-resolved de novo assembly for each individual using hifiasm^30^, resulting in two sets of chromosome sequences, which we refer to below as haplotypes (Fig. 1a). We then separately aligned each read to each of its donor’s two haplotypes, retaining for further analysis only 196,296,004 reads that map unambiguously to both haplotypes and satisfy other quality control filters (Methods). Mentions below of ‘all reads’ refer to this set.

**Extended Data Fig. 1:**
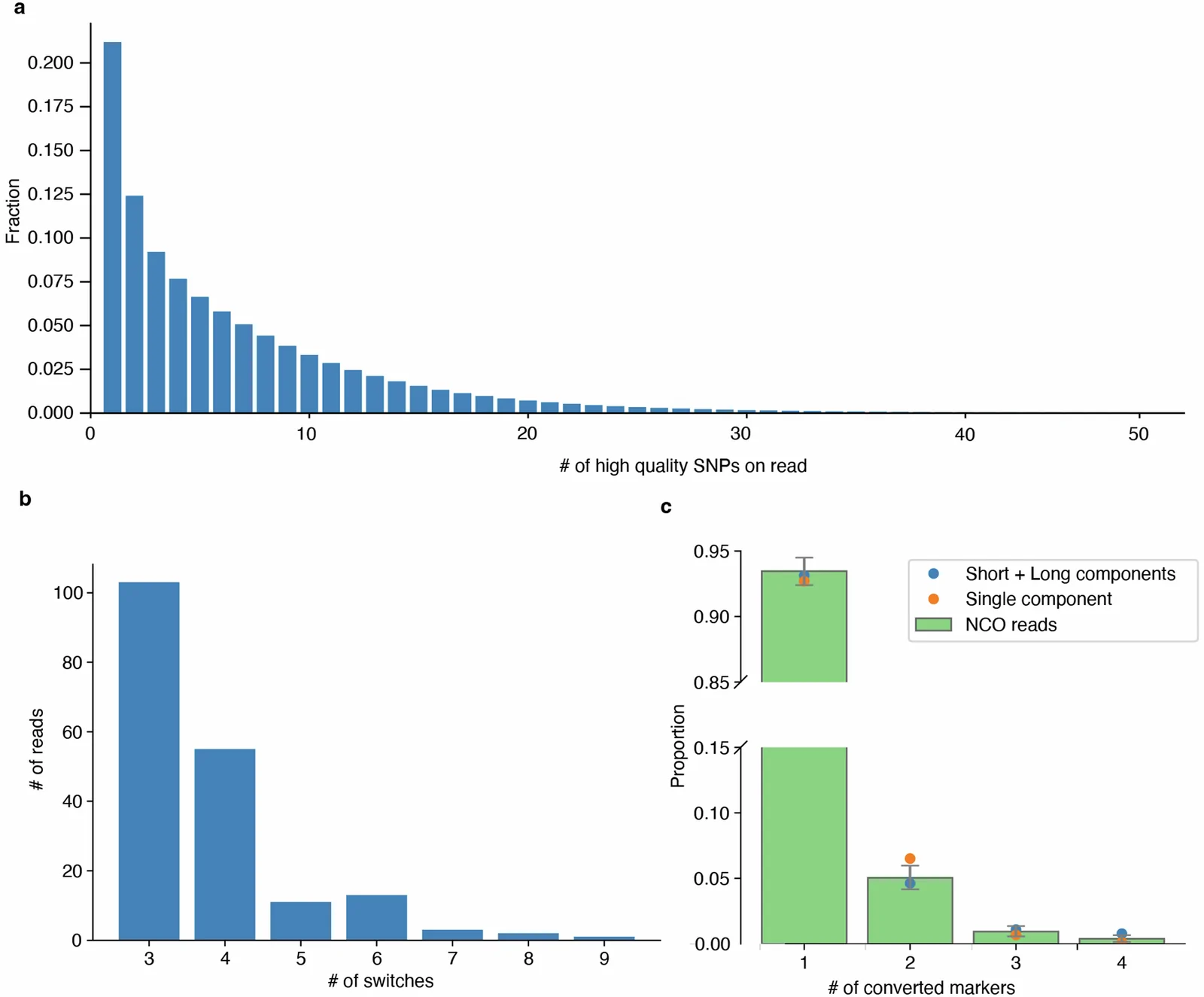
Distributions of SNVs, switches, and converted SNP counts in recombinant reads. **a**, The distribution of the number of SNVs passing quality control on reads. **b**, The distribution of the number of observed switches in reads with complex recombination events. **c**, Fit of the observed number of consecutive converted SNVs in n = 2,143 NCO reads to single geometric and mixture of short and long geometric tract length distributions, with 95% binomial confidence intervals indicated.

We next generated candidate single nucleotide variant (SNV) calls on each read separately, identified as base calls that matched to one haplotype but not the other. After removing candidate SNVs with low base quality (BQ) score, in regions of low complexity, tandem repeats or those mapped to haplotype regions where few other reads aligned (Methods), this resulted in 1,163,633,539 SNVs across all reads, with a mean 5.9 SNVs per read (Fig. 1b and Extended Data Fig. 1a).

**Fig. 2:**
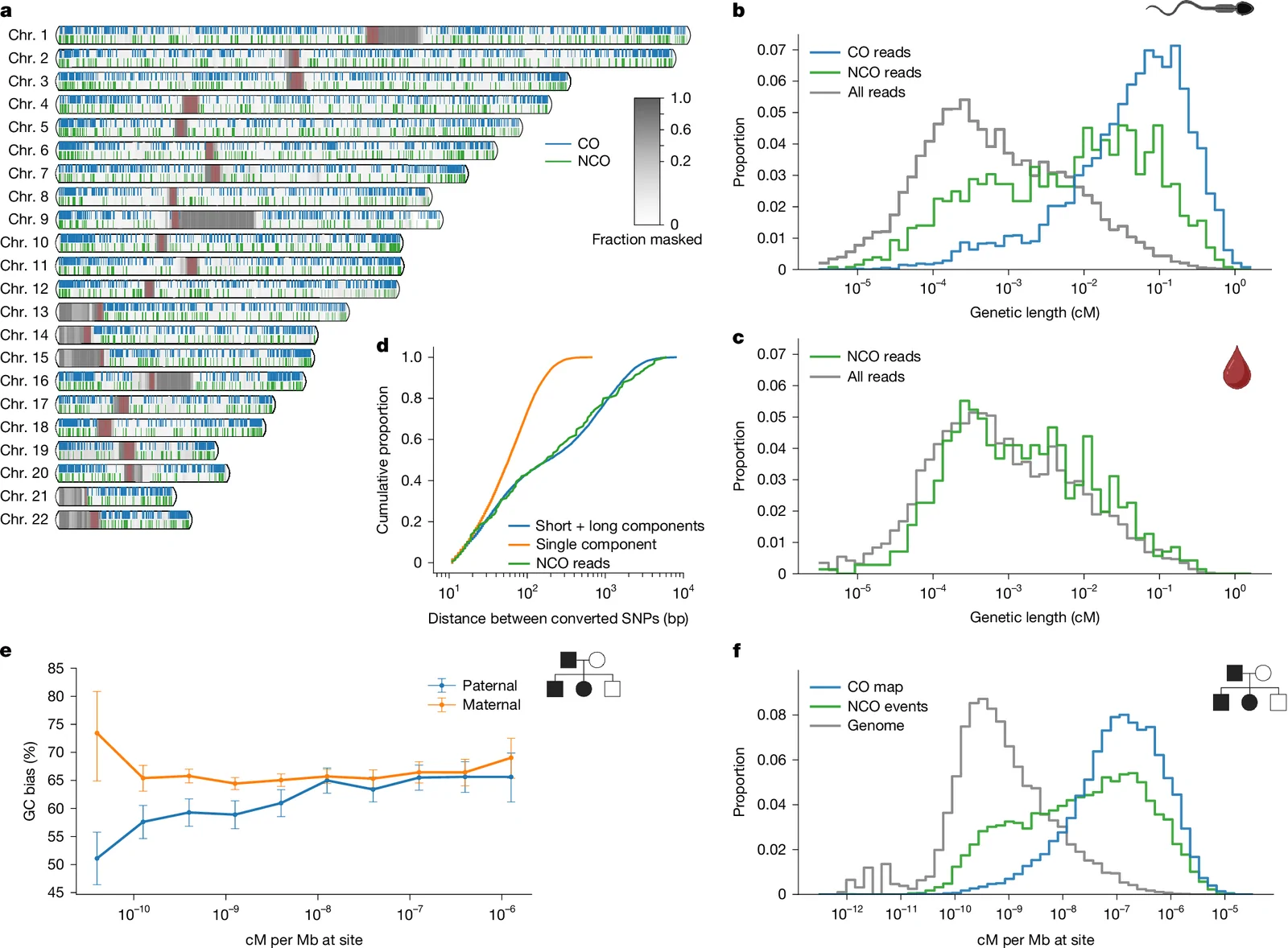
CO and NCO properties. **a**, Genomic distribution of called events: candidate COs (blue), NCOs (green) and centromeres (red). The grey scale shows the fraction of callable bases in 1 Mb windows. Chr., chromosome. **b**,**c**, Enrichment of recombinant reads of COs and NCOs from sperm sequencing (**b**) and NCOs from blood sequencing (**c**) as a function of length of the read in cM according to the male genetic map. **d**, Fit of one-component and two-component models to the distribution of distances between the outer pair of converted SNVs in an NCO when there is more than one converted SNV. **e**, gBGC at converted NCO SNPs in the deCODE dataset, partitioned by whether conversion happened paternally (*n* = 18,836 converted SNPs) or maternally (*n* = 59,904 converted SNPs), with 95% binomial confidence intervals indicated. **f**, Distribution of male CO map rates at paternal deCODE converted NCO SNPs in green, compared with full genome and the CO site distribution. Icons in **b**,**c**,**e**,**f** created in BioRender; Rahbari, R. https://BioRender.com/0iaq75j (2026).

By construction, each allele matches one of the two haplotypes. We designated a read as a candidate recombinant read if it carried alleles from both haplotypes and met several additional quality control criteria (Methods). We label a candidate read as follows: ‘CO’ if it contains a single switch with at least two SNVs supporting it on each side (for example, two or more SNVs from haplotype 1 to the left and two or more SNVs from haplotype 2 to the right); ‘NCO’ if there are two switches; ‘complex’ if it has three switches or more; or ‘ambiguous’ if there is only one switch but not enough SNVs supporting it (that is, only one SNV on one side or the other). We improved resolution for these steps by including some previously filtered SNVs, and excluded a few NCOs after manual inspection (Methods). Ultimately this resulted in 7,143 COs, 2,382 NCOs, 219 complex and 7,585 ambiguous events across all sperm samples (Fig. 2a, Extended Data Table 1 and Supplementary Tables 1 and 3). The number of switches in complex events varies between 3 and 9, with 3 and 4 being most frequent (Extended Data Fig. 1b).

**Extended Data Fig. 2:**
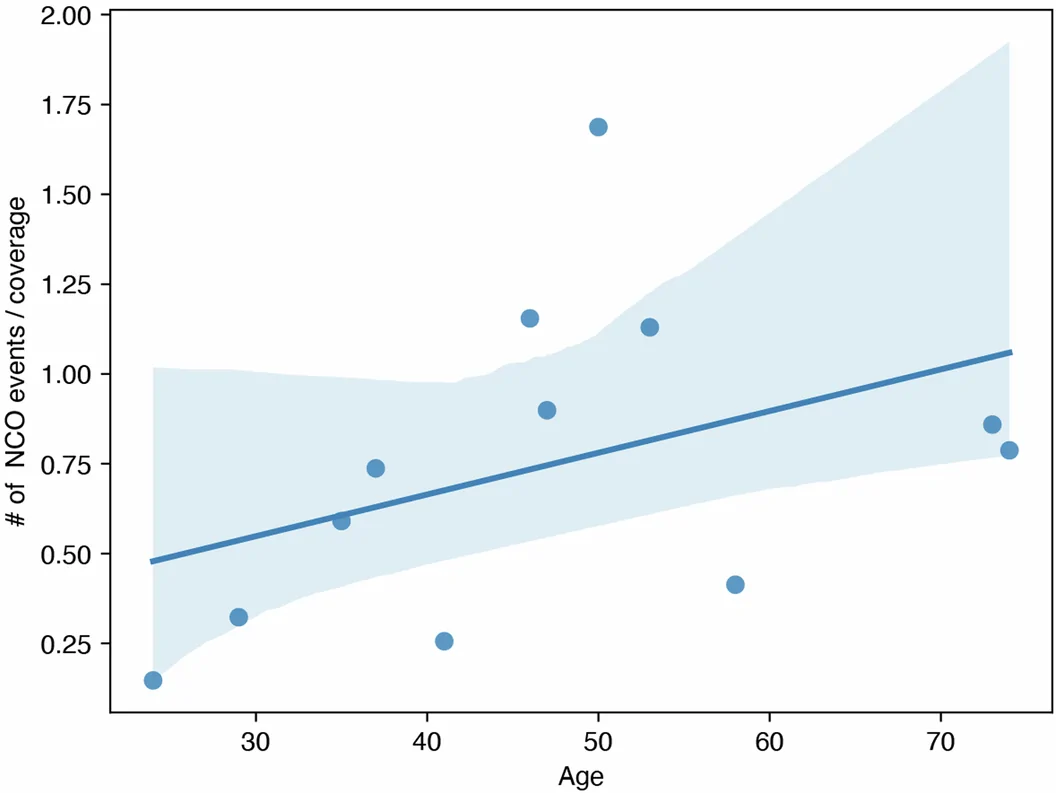
Recombination event counts by donor age. The number of total recombination events detected in blood samples vs. age of the sample. We observe a slope of 0.0157 additional events per genome per year. The solid blue line shows the fitted linear regression estimate, with 95% nonparametric bootstrap CI shaded in blue.

We processed the Platinum Pedigree blood sequence data as the sperm reads, aligning to the assemblies from the Platinum Pedigree study. These resulted in 389,685,716 high-quality read SNV calls, from which we detected 11 COs, 369 NCOs, 9 complex events and 143 ambiguous events. After correcting for coverage these represent 0.4%, 40%, 4.8% and 18.7%, respectively, of the rates seen in sperm samples. The near-complete lack of CO events is consistent with suppression of COs between homologues in somatic tissues^31^. There is some support for blood NCO events being better fit by a process proportional to age (0.016 events per genome per year) rather than being independent of age (likelihood ratio 16.85; Extended Data Fig. 2 and Methods), but with the small sample size this does not reach statistical significance (permutation *P* = 0.1).

## A pre-meiotic component to germline NCOs

**Extended Data Fig. 3:**
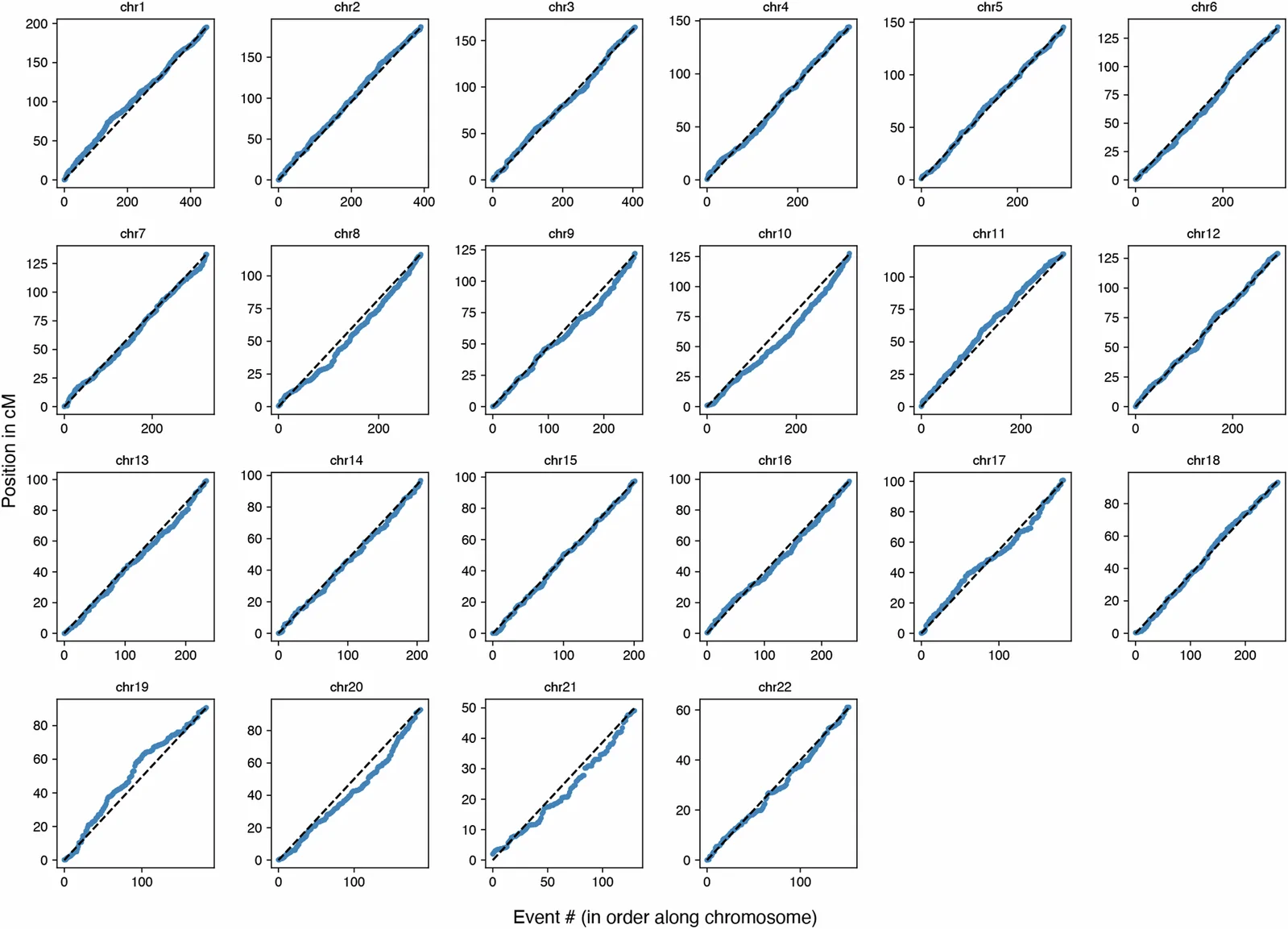
Crossover read positions. The positions of CO reads along the chromosomes, in genetic coordinates.

When combining all sperm samples, the distribution of detected CO events along each chromosome largely follows the male-specific genetic map^32^ as expected (Extended Data Fig. 3). The average map recombination rate of CO events is 30.4 cM Mb^−1^, much higher than the background rate of 1.1 cM Mb^−1^ in all reads (Methods; Kolmogorov–Smirnoff two-sample test, *P* < 10^−10^). By contrast, the average map recombination rate at NCO converted markers is 14.4 cM Mb^−1^, significantly lower than the rate for COs, although still enriched compared to the background rate (Kolmogorov–Smirnoff two-sample test, both *P* < 10^−10^). Finally, complex events show even less correspondence with the genetic map, with an average rate across whole reads of only 2.4 cM Mb^−1^, substantially lower than either COs (8.8 cM Mb^−1^ across whole reads) or NCOs (4.2 cM Mb^−1^) and close to the background level (1.1 cM Mb^−1^).

To further control for possible ascertainment effects induced by variation in SNV density, we compared the distribution of genetic lengths between the second SNV and second-to-last SNV of CO, NCO and all reads (Fig. 2b and Methods). All three distributions, of CO, NCO and all remaining reads are significantly different (Fig. 2b, Kolmogorov–Smirnoff two-sample test, all *P* < 10^−10^). Notably, the NCO distribution is bimodal, with one component overlapping the high-recombination-rate peak characteristic of meiotic COs and another aligning with the low-rate background of non-recombinant reads, representing a substantial fraction of all NCOs. This bimodal pattern was reproducible across both the TwinsUK and SL datasets.

These observations suggest that a fraction of sperm NCO events may arise from a separate origin distinct from the PRDM9-mediated DSBs giving rise to COs. In plants, large-scale studies have suggested that a substantial fraction of inherited NCO gene conversions arise from mitotic processes between the zygote and the gamete^33^. During spermatogenesis, spermatogonia undergo multiple mitotic divisions before entering meiosis, and repair of DNA damage by recombination prior to the programmed meiotic DSB activity may produce gene conversion events that are transmitted to sperm, providing a potential source of PRDM9-independent NCOs^34^. Such NCOs should exhibit distinctive properties. First, they would not align with PRDM9 binding sites or known meiotic DSB hotspot maps. Second, they may be enriched at fragile genomic sites prone to breakage. Finally, because they arise in a different context, they may have different GC-biased allele transmission properties from meiotic NCO-mediated gene conversion; GC bias is known to vary across event types and species^12,35,36^.

To provide a model for non-meiotic repair processes, we examined NCOs detected in the Platinum Pedigree blood samples. In contrast to sperm, the distribution of NCOs in blood was unimodal and overlapped the low-rate background, with no enrichment at high-recombination-rate regions (Fig. 2c). This supports the interpretation of dual origins of NCOs in sperm, arising from non-meiotic repair processes akin to those operating in somatic cells, as well as PRDM9-mediated meiotic events.

## NCOs at DSB hotspots and fragile sites

An alternative explanation for lower conformance of NCOs than COs to the CO map is that the observed NCOs in sperm do arise during meiosis, but have different patterns of enrichment at PRDM9-directed DSB sites. To assess this possibility, we compared CO and NCO regions to a previously published meiotic DSB map of 28,286 human recombination hotspots, based on the measurement of the occupancy of a meiotic DSB repair protein (DMC1) in chromatin from testes of a PRDM9_A_ homozygous male^37,38^. Each called hotspot is centred around a 31 bp PRDM9 motif^38^, which we refer to as DSB-PRDM9 motifs. Since this map was created from the breaks, not how they were resolved, PRDM9-directed meiotic NCOs and COs should be equally consistent with it.

We found that CO locations closely follow the DSB map: 67% of CO reads overlapped a DSB-PRDM9 motif, compared with 13.5% of all reads (Fisher’s exact test, *P* < 10^−10^). Among the remaining CO reads not overlapping a DSB-PRDM9 motif, 58% contained a PRDM9 motif compared to 25% of all non-overlapping reads (Methods). Conversely, only 45% of NCO reads overlapped a DSB-PRDM9 motif (compared with 13.5%; Fisher’s exact test, *P* < 10^−10^), and among the remaining NCO reads, 43% contained a PRDM9 motif (Methods). These proportions suggest that the NCOs are a mixture between the canonical meiotic class induced by PRDM9 and another class of PRDM9-independent events, consistent with the bimodal distribution observed above. A similar pattern was seen for somatic NCOs in blood, where 12.4% overlapped DSB-PRDM9 motifs (statistically indistinguishable from the background rate) further supporting the presence of a non-meiotic subset of NCOs.

In CO reads overlapping a DSB-PRDM9 motif, the motif lies within the CO interval 58% of the time, whereas for NCO reads, the motif is contained within the tract bounded by the flanking markers 62% of the time (not significantly different). By contrast, only 21% of complex event reads contain a PRDM9 motif (not significantly different from background 25%).

We next examined the association of NCO events with a map of human genome fragile sites^39^, which we expect to be enriched for non-meiotic DSB sites. NCO reads with genetic length below 10^−3^ cM overlap fragile regions significantly more frequently (26.2%) than those with genetic length above 10^−3^ cM (21.3%, Fisher’s exact *P* = 0.011), CO reads (21.5%, *P* = 10^−7^), or all recombinant reads (21.9%, binomial *P* = 9.4 × 10^−4^), and near significant in comparison to all reads (23.3%, binomial *P* = 0.066). The fraction of NCO reads from blood samples overlapping fragile sites is 26.1%, essentially the same as the 26.2% fraction for NCO reads from sperm with genetic length below 10^−3^ cM.

## GC bias in NCO tracts

**Extended Data Fig. 4:**
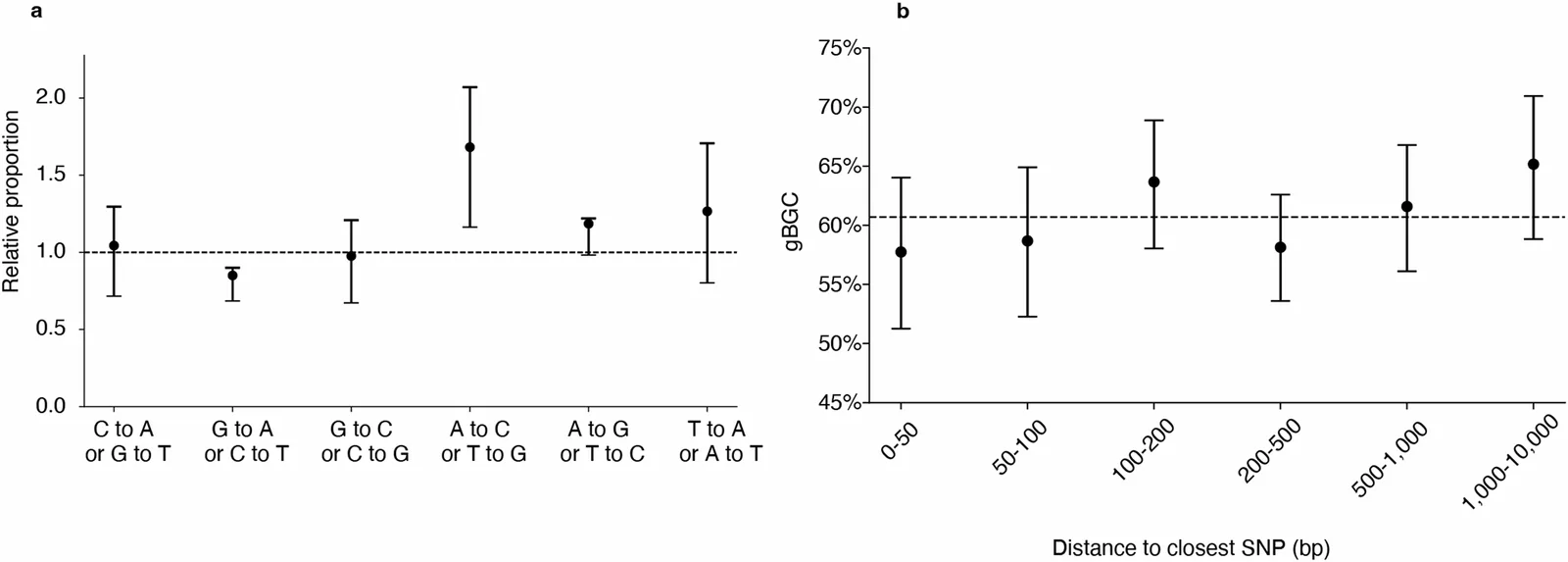
GC bias in converted SNPs. **a**, Proportion of observed single-SNP NCOs for each strand-equivalent donor/recipient allele combination, normalized by the proportion at nearest non-converted markers (unbiased for GC content) across n = 2,608 converted SNPs. Points indicate observed normalized proportions; vertical lines indicate 95% confidence intervals (binomial test). **b**, GC-bias in groups of converted SNPs, binned according to their distance to the nearest SNP across a total of n = 2,099 converted SNPs. Points indicate observed GC-bias proportions; vertical lines indicate 95% confidence intervals.

We next examined whether NCO tracts exhibit the characteristic GC-biased gene conversion (gBGC) associated with meiotic mismatch repair, which reflects the preferential incorporation of G or C nucleotides during repair. We observed a GC bias of 60.7% (confidence interval 58.4–63%) in 1,815 converted single nucleotide polymorphisms (SNPs) in 1,692 NCO events. This is towards the lower end of the Halldorsson et al.^7^ estimate for NCOs in male individuals (confidence interval 57.1–69.1%) and in line with a recent deCODE study^3^ that reported 61.9% (confidence interval 60.6–63.3%). When we limit our analysis to NCO reads (either single or multi-SNV tracts) overlapping a DSB-PRDM9 motif, we do observe a significantly higher value of 65.7% (*n* = 841, confidence interval 62.4–68.9%, Fisher’s exact test, *P* = 6.2 × 10^−5^). Specific relative rates of conversion for each possible base change are shown in Extended Data Fig. 4a. In contrast to a previous result in mice^12^, but consistent with a more recent analysis^40^, we see no significant difference to this rate in multi-SNP tracts (62.3%, *n* = 218, confidence interval 55.7–68.6%), and no significant difference in the GC bias as a function of distance to the nearest adjacent SNP (Extended Data Fig. 4b).

In contrast to sperm, somatic NCOs detected in blood show no evidence of GC bias, with a bias of 44.4% (confidence interval 40.9–47.9%), indistinguishable from random expectation. The overall GC bias of 60.7% observed in sperm is therefore again consistent with a mixture of two components, a PRDM9-dependent class showing strong bias (65.7%) and a non-meiotic class resembling the unbiased pattern seen in somatic cells, further supporting the dual-origin model of sperm NCOs.

## Pedigree evidence for pre-meiotic NCOs

To further assess whether the PRDM9-independent component we observe in sperm reflects a genuine biological signal rather than a feature of long-read detection, we analysed the deCODE pedigree dataset^3^, which identifies NCOs through multi-generational transmission rather than haplotype switches in reads. Stratifying paternal NCOs by local male recombination rate at converted SNPs, we reproduce the same bimodal structure seen in our data: one component enriched in high-recombination regions consistent with canonical meiotic activity, and a second component overlapping the background distribution (Fig. 2f). The recurrence of this bimodal pattern in a pedigree-based dataset argues strongly against a technical explanation tied to our sequencing or filtering strategy. Consistent with our low-recombination sperm read-derived NCOs being more associated with fragile sites than high-recombination NCOs, the fraction of paternal deCODE NCOs from regions with recombination rate below 10^−8^ cM Mb^−1^ that overlap fragile sites (25.6%) is significantly higher than that for high-recombination rate NCOs (22.2%; Fisher’s exact *P* = 0.0001).

gBGC in the deCODE data shows a clear dependence on local recombination rate in the paternal lineage. When paternal NCOs are binned by cM Mb^−1^ at the converted site, GC bias is highest in regions of elevated recombination and declines progressively towards approximately 50% in the lowest-rate bins, indicating a loss of detectable gBGC in the low-rate component (Fig. 2e). By contrast, maternal NCOs exhibit a stable GC bias across the same range of recombination rates, with no systematic attenuation at low cM Mb^−1^ (Fig. 2e). Because paternal and maternal events were processed identically, this sex-specific pattern cannot be attributed to differences in detection or filtering. Rather, it supports a paternal NCO component arising from a process that is distinct from canonical meiotic recombination and lacks measurable GC bias.

## Pre-meiotic germline NCOs in testis

To directly assess whether the PRDM9-independent NCO component observed in sperm has pre-meiotic origins, we re-analysed the human long-read sequencing data of Porsborg et al.^26^. We applied our pipeline to call recombination events separately in the 3 sperm samples (977 COs and 241 NCOs) and 6 testis biopsies (503 COs and 307 NCOs). NCOs showed a clear enrichment in genomic regions with low recombination rates and a corresponding reduction at canonical recombination hotspots, whereas COs remained strongly concentrated in high-recombination-rate regions, reproducing the pattern observed in our dataset.

**Extended Data Fig. 5:**
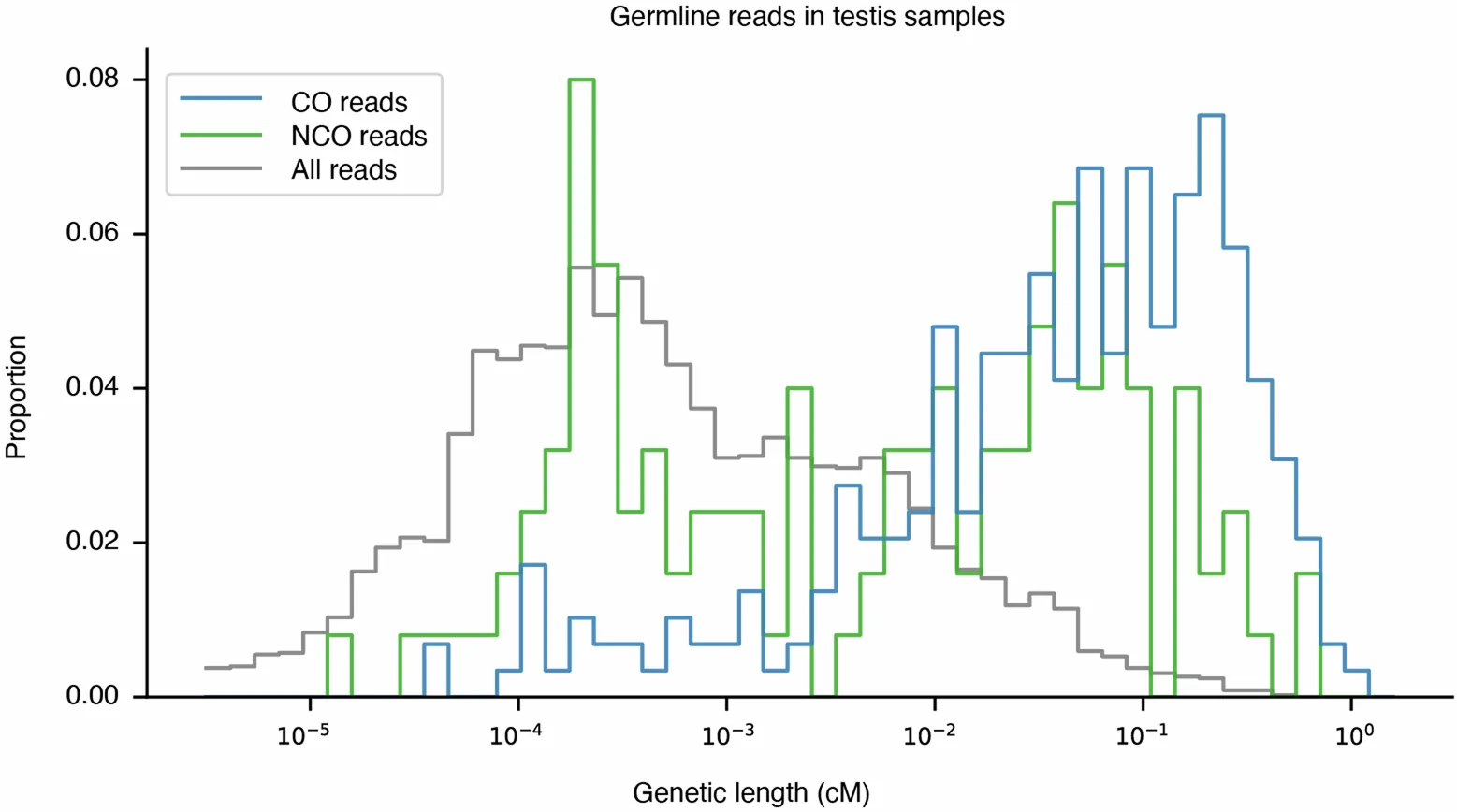
Recombination in germline cells from testis. Enrichment of recombinant reads of COs and NCOs from germline cells obtained from testis tissue samples.

Testis tissues contain pre-meiotic (for example, spermatogonia) as well as post-meiotic germline cells, providing a more direct opportunity to detect products of pre-meiotic DNA repair. We used the methylation-based classification of sequencing reads provided in Porsborg et al.^26^ to classify reads from testes as originating from either somatic or germline cells. The high-confidence (>2 likelihood ratio) germline subset contained 22,093,802 reads with 125 detected NCOs. This subset, which includes a large fraction of pre-meiotic cells, displays a pronounced low-recombination-rate component, closely mirroring the PRDM9-independent NCO signature seen in sperm and strongly supporting a pre-meiotic origin of these events (Extended Data Fig. 5). We observed no gBGC in NCOs observed in testis tissue (50%, confidence interval 45.8–54.3%), consistent with an unbiased transmission pattern and in contrast to the GC bias seen at PRDM9-associated NCOs in sperm.

## Individual variation in COs and NCOs

A key advantage of sequencing sperm directly is that we can observe large numbers of recombination events per sample, proportional to read coverage rather than the number of offspring of an individual. Increasing coverage gives greater power to investigate variation between individuals or between samples from the same individual.

**Fig. 3:**
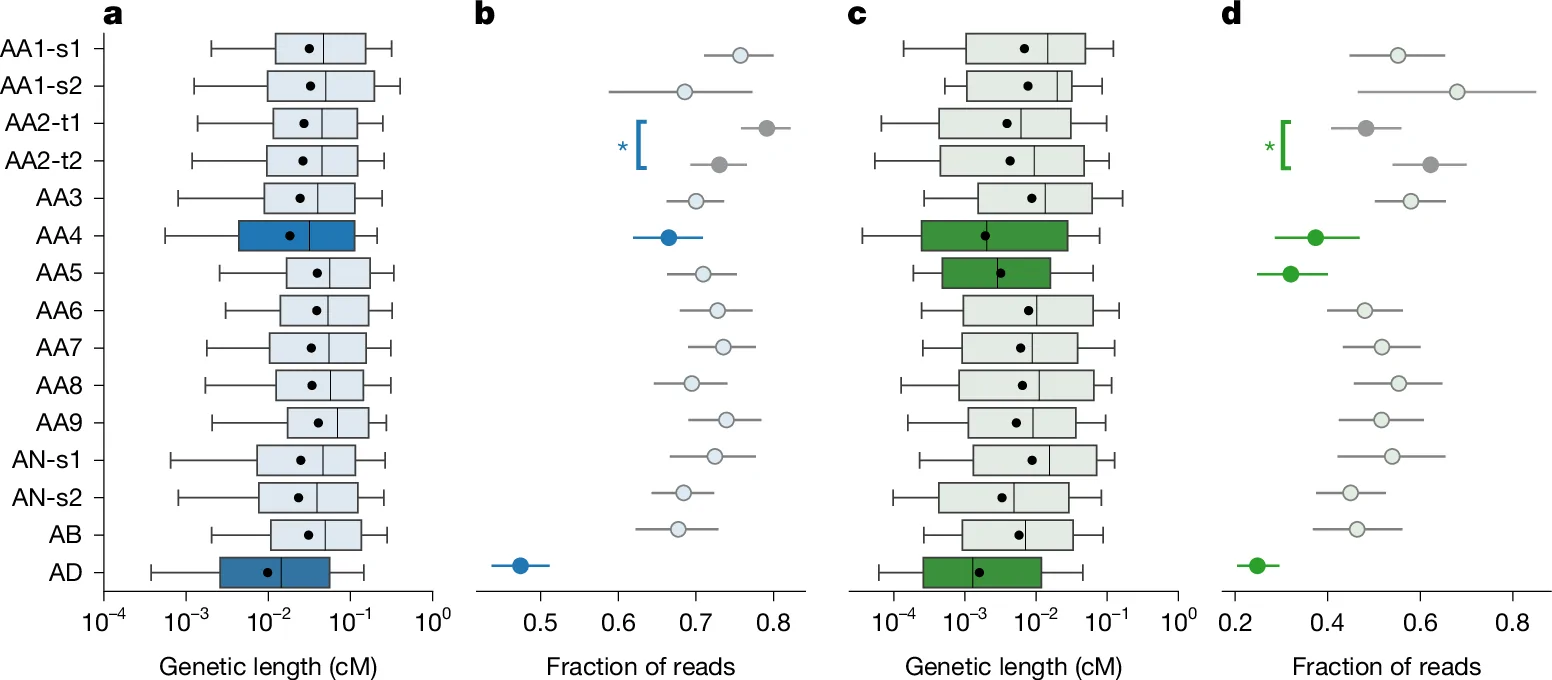
Individual variation in properties of meiotic recombination. **a**, The distribution of genetic length of CO reads (*n* = 6,532 reads from 15 samples from 13 donors: AA1–AA9, AN, AB and AD). **b**, The fraction of CO reads that overlap a DSB-PRDM9 motif. **c**, The distribution of genetic length of NCO reads (*n* = 2,143 reads). **d**, The fraction of NCO reads that overlap a DSB-PRDM9 motif. In **a**,**c**, boxes show the 25%, 50% and 75% quantiles, whiskers indicate 10% and 90% quantiles, and the black circle indicates the mean. In **b**,**d**, circles indicate the observed fraction and the lines indicate the 95% binomial confidence interval. Coloured boxes and circles indicate samples that are statistically significantly different from other samples after Bonferroni correction (two-sample Anderson–Darling statistic with permutation testing; all *P* < 10^−4^). Brackets with asterisks for AA2 comparisons indicate results that differ significantly between monozygotic twins (Fisher’s exact test; *P* = 0.04 for COs, *P* = 0.01 for NCOs).

**Extended Data Fig. 6:**
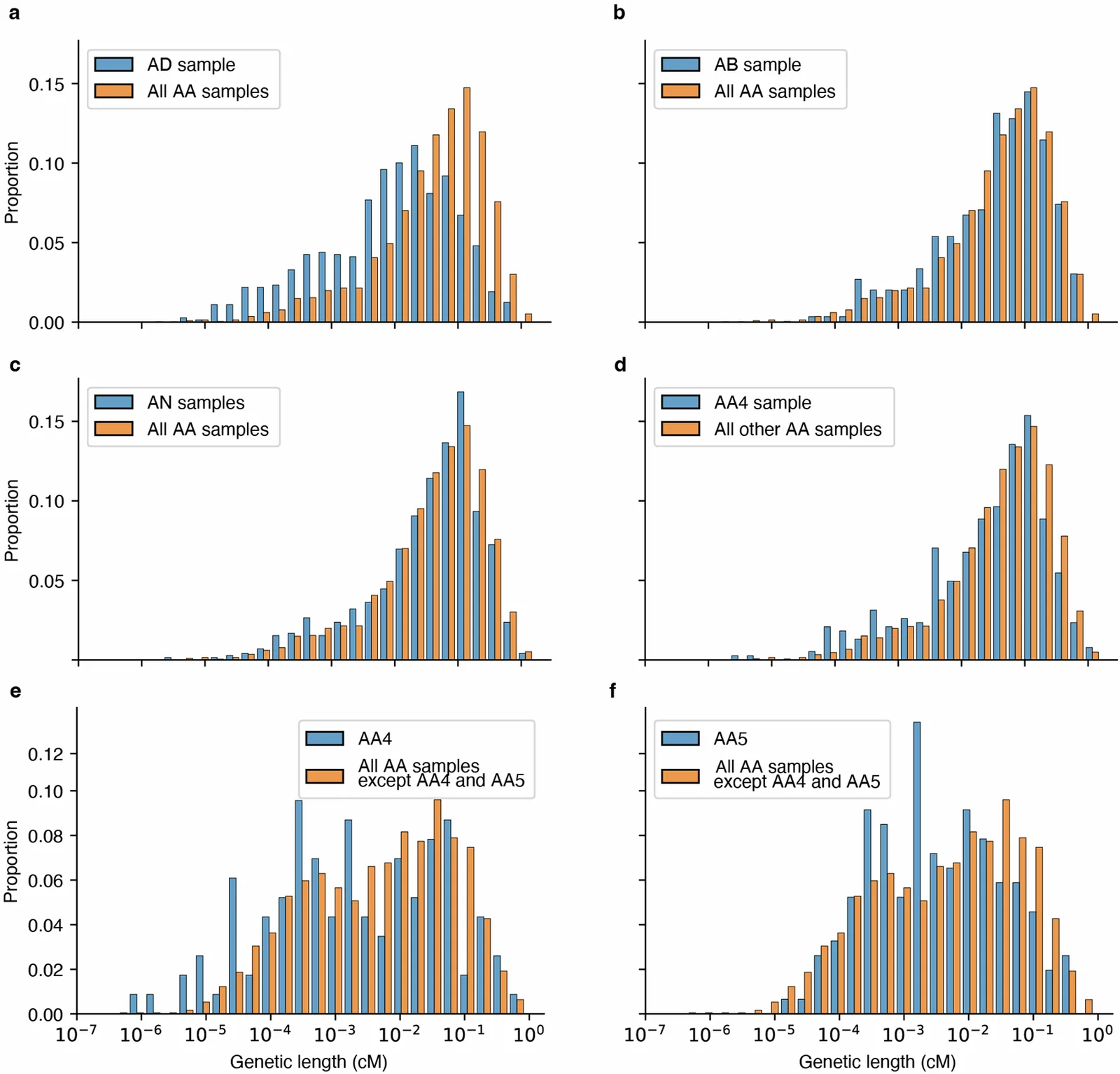
Differences in crossover localization across samples. The difference in distribution of genetic length (in cM) of CO reads between: **a**, the AD sample and the A/A samples (two-sample Anderson-Darling statistic with permutation testing, P < 1e-4); **b**, the AB sample and the A/A samples (P < 1e-4); **c**, the AN samples and the A/A samples (non-significant after Bonferroni correction; P = 0.05 and P = 0.71 for AN-s1 and AN-s2 respectively); **d**, the AA4 samples and the other A/A samples (P < 1e-4). The difference in distribution of genetic length (in cM) of NCO reads between **e**, the AA4 sample and all other A/A samples except AA5 (two-sample Anderson-Darling statistic with permutation testing, P < 1e-4); **f**, the AA5 sample and all other A/A samples except AA4 (P < 1e-4).

To this end, we examined the variability between individuals according to two different measures for both COs and NCOs: first, the distribution of genetic lengths of recombinant reads, which reflects the degree that they follow the CO recombination map (Fig. 3a); and second, the fraction of reads that overlap a DSB-PRDM9 motif (Fig. 3b). COs from donor AD had significantly smaller genetic length (Anderson–Darling statistic with permutation testing, *P* < 10^−4^) and lower DSB-overlapping fraction (Fisher’s exact test, *P* < 10^−10^) than those from AA, consistent with the PRDM9_D_ map differing from the standard European map dominated by PRDM9_A_ (Fig. 3a,b and Extended Data Fig. 6a). NCOs in donor AD show a similar but weaker genetic length effect (*P* < 10^−4^). By contrast, donor AB exhibited less difference from donors AA1–AA9 (Fig. 3a and Extended Data Fig. 6b,c), in line with previous reports of a similar DSB map between AA and AB genotypes^37^. Indeed, AA4 showed larger differences from the other AA samples than from AB (Extended Data Fig. 6d). With respect to NCOs, along with AA4, AA5 reads were also significantly less concordant with both the genetic map and the DSB hotspot map than other AA samples (permutation testing *P* < 10^−4^ for both; Fig. 3c and Extended Data Fig. 6e,f). Notably, we also observed significant differences in the fraction of DSB-overlapping reads between the monozygotic twins AA2-t1 and AA2-t2 (Fisher’s exact test *P* = 0.04 for COs, *P* = 0.01 for NCOs; Fig. 3d).

## NCO tract lengths and positioning

Finally, we investigated gene conversion tract lengths and positioning. NCO tracts are not observed directly: they only leave evidence at heterozygous markers that are converted. Most tracts convert only a single SNP (2,222 out of 2,382 candidate NCO reads; 93.3%), although there is one that converts 10 SNPs. We therefore use a statistical approach to infer the distribution of tract lengths (Methods). As others have done^12,40–43^, we model the tract length as following a geometric distribution, corresponding to a constant probability of it ending at each base. When we fit a single geometric distribution, we inferred a mean tract length of 71 bp (95% nonparametric bootstrap confidence interval 60.5–89.3 bp). However, the expected distribution of the number of converted markers that this implies is inconsistent with the observed data, with an excess of long tracts (Fig. 2d and Extended Data Fig. 1c), as observed previously^6,7^. Indeed, in a recent pedigree study of baboons^11^ a non-negligible fraction of long (greater than 1 kb) NCO tracts was also observed, and a mixture of two geometric distributions (one short with mean around 24 bp and one long with mean greater than 4 kb) fit the data better; a similar two-component model was fit in the recent analysis of the deCODE human pedigree data^3^. Consequently, we also fit the tract length distribution as a mixture of two geometric distributions, leading to a substantially better fit for the expected distribution of converted markers (Fig. 2d and Extended Data Fig. 1c), with a mixture proportion of 0.9% (95% nonparametric bootstrap confidence interval 0.6–1.2%) of long tracts with mean 1,220 bp (confidence interval 680–1,520 bp), on top of the major component capturing short tracts with inferred mean 31 bp (confidence interval 22–41 bp). Although only approximately 1% of NCO events come from the longer component, this suggests that about 12% (confidence interval 11.4–12.5%) of detected NCOs are caused by long events. Indeed, because most NCO tracts are short, around 99% (confidence interval 98.9–99.2%) of them will not be observed because they do not overlap a heterozygous site.

**Extended Data Fig. 7:**
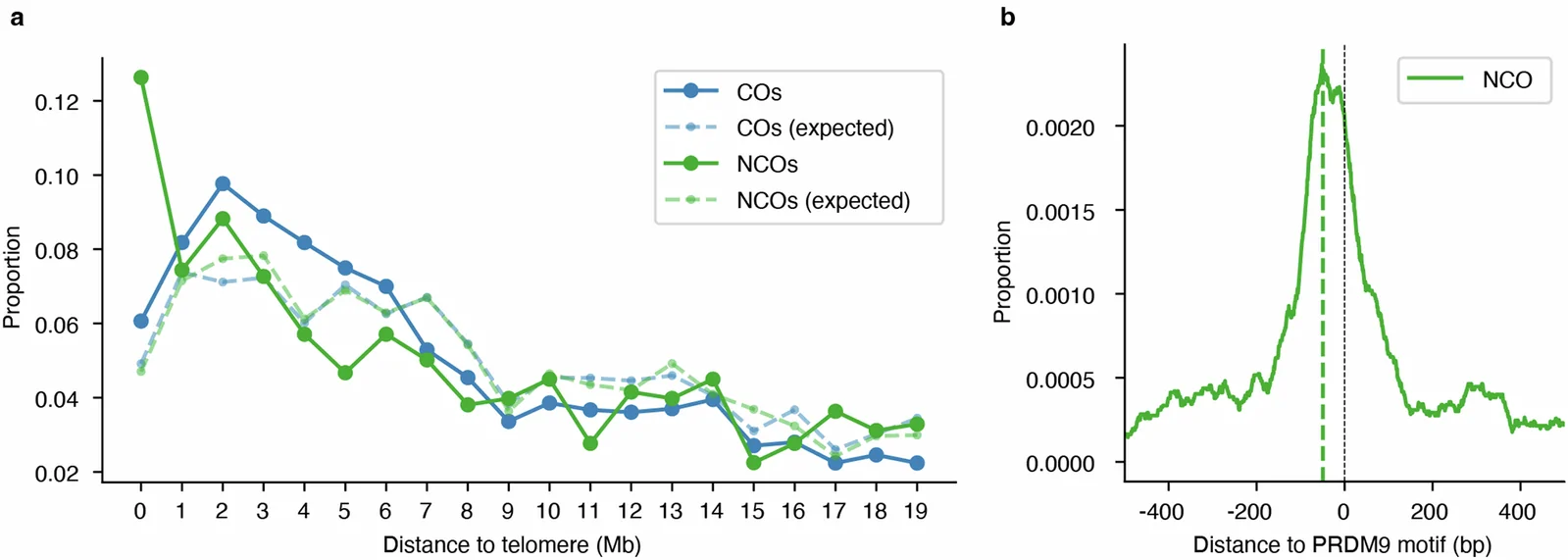
Recombination event localization near telomeres and PRDM9 motifs. **a**, The distribution of distances of CO and NCO events from telomeres, for events that occur within 20 Mb of chromosome ends, and excluding acrocentric chromosomes. The expectation is calculated taking into account the CO recombination rates and the marker patterns on reads, to account for hotspots and marker density. **b**, Relative positions of converted SNVs for called NCOs relative to the closest oriented DSB-PRDM9 motif.

Mapping of long reads to an individualized assembly enabled us to call CO and NCO events in repeat-rich regions such as the subtelomeric regions, previously inaccessible to short-read based pedigree studies or earlier sperm sequencing studies. An assessment of the genomic distribution of CO and NCO events showed that both are elevated near telomeres, consistent with previous results^7,12^ (Extended Data Fig. 7a), with more NCOs than COs in the 1 Mb region nearest the telomere, which we confirmed is not due to an excess of marker density leading to higher NCO detectability (Methods). We note an apparent difference in the distribution of NCOs between the TwinsUK and the SL samples, which is not explained by age or sequencing parameters (Supplementary Fig. 6).

Finally, the localization of PRDM9 motifs within DSB hotspots allows us to study the relative positions of recombination events to PRDM9 binding sites, as recently also reported by the deCODE group^3^. Focusing on NCO reads containing a DSB-PRDM9 motif, we estimated the distribution of offsets between converted markers in NCO reads and the centre of the oriented DSB-PRDM9 motif. Because NCO tracts are typically short (less than 100 bp, see below), focusing on converted markers allowed us to localize the recombination breakpoints with high resolution. The resulting distribution showed an average distance of around 285 bp to the motif centre, but this pattern was asymmetric, with 60% of the converted bases occurring upstream of the DSB-PRDM9 motif, at a median distance around −34 bp (Extended Data Fig. 7b, permutation test *P* = 10^−4^), and only 40% occurring downstream. This suggests a directional influence of PRDM9 binding on the location of NCO events.

## Discussion

Here we exploit long sequencing reads from sperm samples to study recombination processes in the male germline. In particular, the very high accuracy of Pacific Biosciences CCS base calls enables us to confidently identify NCO events, as recently done across primates^26^, and extend beyond previous genome-wide sperm sequencing studies that were limited to CO events^8,21,25^.

**Fig. 4:**
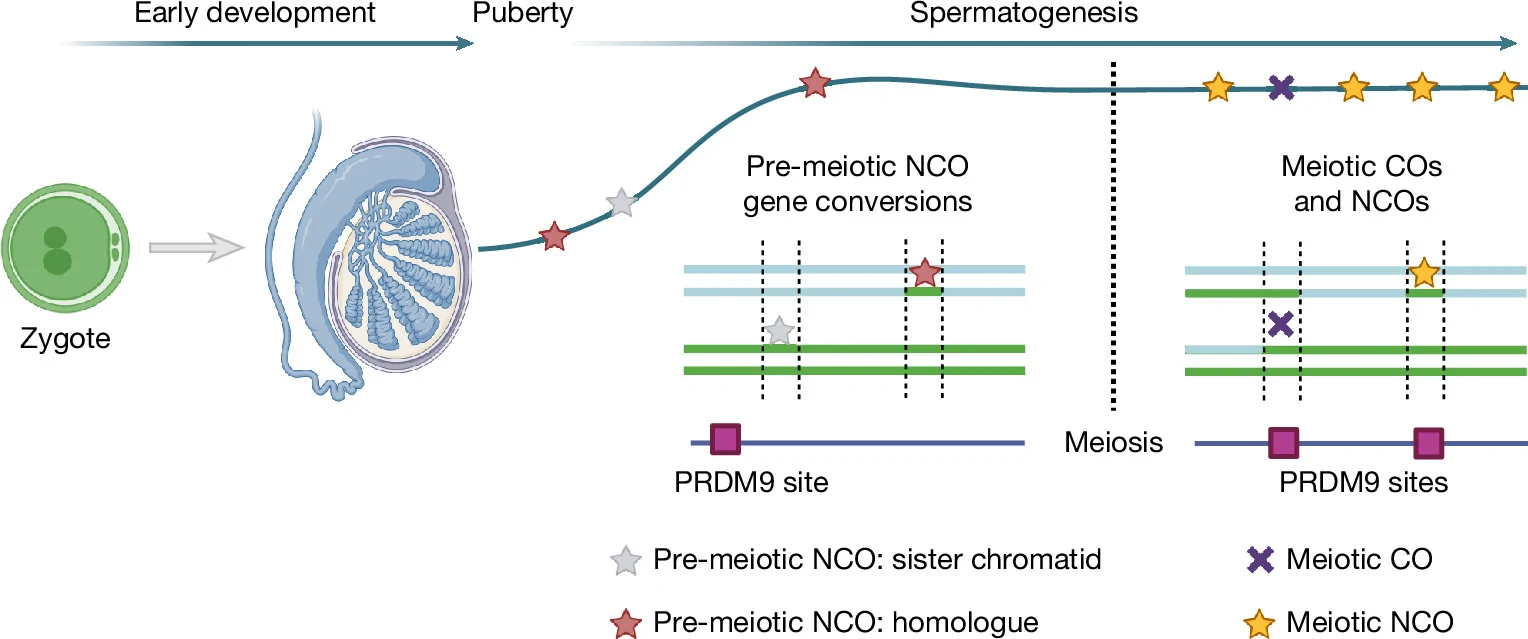
Origins of meiotic and pre-meiotic recombination events in the male germline. Pre-meiotic mitotic repair away from PRDM9 sites yields NCO gene conversions (sister or homologue template), whereas meiotic DSB repair at PRDM9 sites produces COs and NCOs; homologue-repaired pre-meiotic NCOs are detectable in sperm along with meiotic NCOs and COs, and are transmitted to the next generation. Created in BioRender; Rahbari, R. https://BioRender.com/qghn20q (2026).

Even in sperm, we expect to see the results of mitotic as well as meiotic recombination processes, arising from repair of DSBs occurring between the zygote and meiosis. Our observation that a substantial fraction of sperm NCOs are located away from meiotic CO hotspots, and that these are associated with fragile sites, as are the corresponding events seen in blood, is consistent with a significant fraction of sperm NCOs arising from pre-meiotic repair (Fig. 4). This is confirmed in paternal NCO events from pedigree data, and supported by a relative increase in this component in reads classified as germline from testis tissues^26^, which include reads from pre-meiotic cells. We do not identify what mechanistically gives rise to these events, but note that they are consistent in principle with gene conversion during recombination-mediated DSB repair, and also that if this is the source it must be from the homologous chromosome rather than the sister chromatid, which does not carry the alternate allele.

The presence of pre-meiotic NCO-type events in sperm in turn can explain, at least in part, the reduction in association of NCOs compared to COs with DSB-PRDM9 sites and the male genetic map. Our data from sperm and blood, and our analysis of the deCODE data, all suggest that the non-meiotic NCO gene conversions show no significant GC bias, explaining the lower level of gBGC observed by Palsson et al.^3^ in male individuals (61.9%) than in female individuals (65.5%, non-overlapping confidence intervals).

We can estimate the fraction of NCOs that are pre-meiotic in multiple ways. From the genetic length distributions (Fig. 2b) we estimate a pre-meiotic fraction of approximately 45%, from the DSB-PRDM9 overlap we estimate 41% and from the GC bias difference we estimate 32%, each with a margin of error (Methods). In a similar manner, we estimate approximately 23% from the deCODE data, based on both genetic length distributions and, separately, on the GC bias difference. There are different potential sources of error and/or confounding for each calculation, and differences in sample preparation and processing protocols, but overall they are consistent. For an overall estimate we would conservatively suggest a pre-meiotic NCO fraction of approximately 25–33%, with around 0.6 detectable events per sperm. This figure is consistent with an a priori range of 0.5–1.0 based on estimates of DSB rates and homologous recombination repair^31,34,44–48^ (Supplementary Note 2).

Beyond identifying this non-meiotic component, we observed individual variation in NCO properties beyond that caused by PRDM9 genotype. In particular we found evidence for differences between samples taken from monozygotic twins, suggesting that stochastic or environmental factors can have a substantial role in meiosis outcomes.

As others have reported^3,11^, our data clearly support that germline NCOs are fit much better by a mixture of two geometric distributions than by a single one. We inferred a short component of mean length under 50 bp (we estimated 31 bp) accounting for approximately 99% of events and a longer component allowing for multi-kilobase tracts that accounts for the remaining events. We do not have enough data to distinguish whether the larger component is itself a mixture. However, we note that as others have observed^3,6,7,26^, we see complex events extending over many SNPs, and suggest that the longer NCO component we detect may come from the two-switch fraction of these complex events, or indeed from longer complex events that extend beyond the end of a read, potentially resolving to a CO, that leave only two switches within the read.

The observation of a tendency for NCOs to be biased upstream of PRDM9 motifs was also reported in the deCODE dataset^3^, subsequent to our preprint^49^. As discussed there, sequencing SPO11-bound oligonucleotides from testes that are released during DSB cleavage has generated a high-resolution map of cleavage near PRDM9 sites in mouse^50^. This revealed a trimodal profile of SPO11-induced cleavage relative to the PRDM9 site when averaging across all sites, and subsequent clustering of individual sites indicated 3 separate classes of PRDM9 hotspots: class 1 cleaving about 50 bp upstream of the PRDM9 site, class 2 cleaving over the site, and class 3 showing more diffuse cleavage with a component downstream. The profile that we observe (Extended Data Fig. 7b) is bimodal, with a relatively stronger peak upstream than in mouse, consistent with a relatively higher fraction of class 1 sites in human than mouse. The mechanism for creating this bias is unknown, but previous studies have established that, as well as trimethylating lysines on the histone H3 tail, PRDM9 remains bound to DNA through subsequent steps in meiosis, interacting with other recombination proteins through its KRAB domain^51^, which would provide the possibility for a directional bias.

The experimental simplicity of using a single sperm sample allows this approach to be used on other species for which sperm, or where that is possible very large quantities of eggs, can be obtained. It could also be applied to study the effects on recombination outcomes of mutations in genes that are known to be involved in recombination, by sequencing sperm from male individuals carrying the mutations. In humans and most other species, a limitation is that the approach can only be used to study male meiosis. Other limitations are that long-range interactions such as CO interference cannot be studied, and the upper length limit of Pacific Biosciences reads means that the full extent of complex events, including whether they resolve to COs or NCOs, cannot be reliably determined.

Finally, we can consider the possibility of much higher sequencing depths of perhaps thousands of fold coverage on just one or a few samples. In principle, this would enable direct study of repeated recombination events in the same sample at individual hotspots and the study of rare events such as non-allelic homologous recombination and recombination-associated mutagenesis^38^. This advance would enable further insights into the processes driving meiotic and non-meiotic recombination and their implications for genetic diversity and evolution.

## Methods

### Ethics

The work reported on TwinsUK samples was carried out with informed consent under TwinsUK BioBank ethics, approved by the North West–Liverpool Central Research Ethics Committee (REC reference 19/NW/0187), IRAS ID 258513 and earlier approvals granted to TwinsUK by the St Thomas’ Hospital Research Ethics Committee, later the London–Westminster Research Ethics Committee (REC reference EC04/015). SL samples were obtained from Discovery Life Sciences as discarded medical waste in accordance with IRB protocol DLS-BB050. These were collected as part of standard-of-care testing ordered by the patient’s physician and excess material released for research.

### Sperm sample extraction and sequencing

We obtained 15 sperm samples from 13 donors of European ancestry. Eleven individuals donated one sample, and two individuals donated two samples at different ages (AA1-s1 and AA1-s2; AN-s1 and AN-s2), with ages at donation varying between 24 and 74 (Supplementary Table 1). Samples AA2-t1 and AA2-t2 are monozygotic twins. The TwinsUK cohort consists of more than 14,000 volunteers, mostly middle-aged female individuals, who have participated over the past 30 years in a longitudinal cohort study. This has included lifestyle and health questionnaires, biomedical measurements, biological sample collection and the generation of multi-omics profiles (such as genetics, metagenomics and metabolomics), over multiple visits.

For the TwinsUK samples, we obtained high molecular weight (HMW) DNA from bulk sperm samples using the Circulomics Nanobind Tissue Kit (102-302-100) and NEB Monarch HMW DNA Extraction Kit for Cells & Blood (T3050) UHMW with some modifications to account for tighter packing of the sperm genome. We supplemented the lysis solution with 150 mM 1,4-dithiothreitol to disrupt protamine disulfide bonds and extended proteinase K incubation from 30 min to 2 h. The rest of the Circulomics HMW DNA extraction protocol remains unchanged. HMW DNA was sheared to 10–14 kb DNA fragments using Megaruptor 3 system (B06010003) with speed setting 30. CCS sequencing libraries were constructed according to the standard CCS library preparation protocol 1.0 (100-222−300), and the libraries were sequenced using a Sequel IIe and Revio instrument at the Wellcome Sanger Institute with full-resolution base quality scores.

For the SL data, samples were thawed on ice and cells were pelleted in an isotonic sperm wash solution to remove debris and reduce somatic cell contamination. DNA was extracted using the NEB Monarch HMW DNA Extraction Kit for Cells & Blood (T3050) UHMW protocol, with some modifications to the cell lysis step. Specifically, cells were digested at 56 °C for 1 h at 300 rpm with 100 µl Nuclei Prep Buffer, 100 µl Nuclei Lysis Buffer, 10 µl Proteinase K (NEB P8107, 20 mg ml^−1^), and 10 µl 1 M 1,4-dithiothreitol (GoldBio in dH_2_O, final concentration ~50 mM). An additional 20-minute digestion was then performed with 5 µl RNase A (NEB T3018, 20 mg ml^−1^). The rest of the protocol remained unchanged. HudsonAlpha performed HMW DNA quality control, CCS library preparation, and sequencing on the PacBio Revio sequencing instrument.

### Blood sample sequencing data analysis

We obtained raw PacBio CCS sequencing data and assemblies from the Platinum Pedigree dataset. Flow cells containing multiple samples were excluded due to cross-sample contamination caused by demultiplexing errors. Additionally, we excluded the four first-generation samples, as they were derived from cell lines. This resulted in a final dataset of 12 samples.

### De novo haplotype-resolved assembly

We used hifiasm^30^ (v0.19.5-r592, default parameters, HiFi only mode) to construct a haplotype-resolved de novo assembly for each sample. The hifiasm assembler outputs a partially phased assembly graph for the two haplotypes, which we converted into two fasta sequence files per sample.

We scaffolded each haplotype onto the T2T-CHM13 reference genome^52^ with RagTag^53^ (v2.1.0, with arguments “-u -w --aligner minimap2”). RagTag orients and places contigs along the T2T reference with gaps; we manually expanded the gaps between adjacent contigs to 30,000N to avoid reads mapping across a gap to two contigs, as this may include a phase switch error which will further be falsely interpreted as a CO.

### PRDM9 genotyping

We obtained the zinc-finger array sequences from 74 PRDM9 alleles reported previously^27^ and mapped them to each assembled haplotype of each donor. For each haplotype, we called the PRDM9 allele as the one with the smallest amount of mismatches, insertions or deletions between the allele sequence and the assembly. All the A alleles, B allele and the D allele were a perfect match, whereas we did not find a perfect match to the new PRDM9 allele of AN-s1/2. In addition, we verified that the flanking regions on the two sides of the sequence match the expected sequences as reported^27^ (up to 2 bp mismatches), and confirmed that PRDM9 allele variations are not due to assembly errors by visually inspecting the assembly around the region.

### Read alignment

CCS reads with residual adapter sequences were identified with HiFiAdapterFilt^54^ and were excluded from downstream sequence analysis. We used minimap2^55,56^ (v2.26-r1175, with arguments “-ax map-hifi --cs=short --eqx --MD”) to map the reads to each haplotype separately. We then filtered each bam file to contain only primary alignments (using the “-F 0×900” flag in samtools view^57^) and with mapping quality MAPQ = 60. We further discarded reads that map to only one of the haplotypes, or that map to different chromosomes. Finally, we discarded reads that map to different strands of the two haplotypes.

### Creating candidate recombinant reads

#### Comparing haplotype alignments

Each alignment of a read to a haplotype effectively partitions the read sequence into segments, where each segment corresponds to an alignment operation: either (1) ‘match’—aligns and matches perfectly to the reference haplotype; (2) ‘mismatch’—aligns but does not match to the reference; (3) an ‘insertion’; (4) a ‘deletion’ with respect to the reference (in a deletion, this read segment is of length 0); or (5) a ‘soft clipping’.

We used the two alignments of the same read to the two haplotypes, and created a refined partition of the read according to both alignments, defined as the partition which is the intersection of the two partitions. In this refined partitioning, each read segment corresponds to two alignment operations. For example, the read segment [0, 40) is both ‘match’ to haplotype 1 and ‘match’ to haplotype 2; the read segment [40, 41) is a ‘match’ to haplotype 1 but ‘mismatch’ to haplotype 2; the read segment [41, 41) is a ‘deletion’ with respect to haplotype 1, but is a ‘match’ (in an empty sense) to haplotype 2; the read segment [41, 50) is a ‘match’ to haplotype 1 but an ‘insertion’ with respect to haplotype 2, and so forth. This refined joint partition is the basis for our follow-up analysis.

We discarded reads with more than 10 bp of soft clipping to either haplotype, as these were observed to often be chimeric reads. Moreover, we discarded reads with more than 100 sequence errors, defined as segments (usually 1 bp) mismatching both haplotypes.

#### SNP calling and filtering

Along each read, we find SNP candidates using the partitioning described above to find single nucleotide positions which match to one haplotype but do not match to the other haplotype. We observed that many such one nucleotide mismatches occur in regions suspicious of assembly errors, such as regions of low complexity, tandem repeats, near read ends and in haplotype regions with low coverage. Therefore, we further filtered SNP candidates according to several criteria.

First, we include only those surrounded by at least 10 bp of matched alignment on both sides of the SNP. Second, we discarded SNPs that were within 1,500 bp of either read ends for the Sequel II data; or 400 bp for the Revio data (Supplementary Methods and Supplementary Figs. 1 and 2). Third, we filtered SNPs with a base quality (BQ) score of less than 60 for the Sequel II data; or 40 or 50 for the Revio data, depending on the largest BQ bin (Supplementary Methods and Supplementary Figs. 3 and 4). Fourth, we ran sdust^58^ (v0.1-r2, with default arguments), an implementation of the dustmasker algorithm^59^, to identify low complexity regions in both haplotypes. We filtered SNPs that were mapped to a low complexity region in either haplotype. Fifth, we ran Tandem Repeat Finder^60^ (v4.09.1, with arguments “2 6 6 80 10 50 500 -ngs -h -l 10”) to find tandem repeats in both haplotypes. We filtered SNPs that mapped to a tandem repeat in either haplotype. SNPs that passed these criteria are considered high-confidence SNPs. We discarded reads that have no such high-confidence SNPs from further analysis.

For the Sequel II data, we called a further set of candidate SNPs satisfying the same criteria, except for a minimal BQ of 30 (instead of 60 in high-confidence SNPs) and a distance of more than 500 bp from read ends (versus 1,500 bp in high-confidence SNPs) as a less strict subset of ‘classification SNPs’ we used for event classification and analysis, but not for event detection. For the Revio data, we used a minimal BQ of 30 (versus 40) and distance of 200 bp (versus 400 bp).

#### Haplotype assignment

We scanned each read for high-confidence SNPs and kept track of which of the two haplotypes each SNP matches to. The large majority of reads (>99.9%) have SNPs that are fully consistent with at least one of the haplotypes, and therefore are not candidates to be recombinant reads. However, their existence is helpful in filtering assembly errors, as we describe below. Therefore, we recorded, for each of the two haplotypes and for each read, the percentage of SNPs in the read consistent with the haplotype.

#### Further SNP filtering measures

We observed that regions in an assembled haplotype that did not have enough consistent reads overlapping them were prone to errors and false detection. We therefore further require that each SNP have at least 3 reads overlapping the focal SNP, which have >95% SNPs consistent with haplotype 1; and similarly for haplotype 2.

#### Recombinant SNP candidates

To create a list of candidate recombinant reads, we calculated again for every read the percentage of high-quality SNPs (after read coverage filtering) consistent with each one of the haplotypes. A read is therefore a candidate if its SNPs are not fully consistent with either haplotype.

### Classifying candidate recombinant reads

#### Further read filtering

Based on the list of classified reads and their annotations, we apply further filtering to reduce false positives. We define a transition pair as a pair of adjacent SNPs on the read which map to different haplotypes. Each candidate read will have at least one transition pair. Two transition pairs (across different reads) that map to the same haplotype coordinates are suspected of being a result of a phasing or assembly error, since we assume every recombination event happens exactly in one read. We therefore discarded reads if they include transition pairs recurring in other reads. Given the sequencing depth of our dataset (~20×–160×; mean 78×), the probability of observing the same recombination molecule more than once by chance is low (see ‘Probability of observing the same NCO event more than once’; ranging from 4.4 × 10^−7^ to 2 × 10^−6^ for CO and 1.6 × 10^−6^ to 7.8 × 10^−4^ for NCO), suggesting that such events are more likely to be the result of technical artefacts.

In addition, if the genomic region between a transition pair is not covered by three or more reads (see ‘Further SNP filtering measures’) in either haplotype, then we deem the assembly in this region as uncertain. In particular, such low coverage regions may indicate a phasing error in haplotype assembly, which may result in false CO calls. We therefore also discard reads with such low coverage transition pairs.

Finally, we discarded reads suspected of cross-sample contamination. A read was classified as a contaminant if it contained a single-base mismatch to both haplotypes at a site of known population variation, indicating it originated from a different genome. To find such reads, we first aligned all reads to the GRCh38 reference genome, and compared the location of 1 bp mismatches to known locations of population variation from the 1000 Genomes Project^61^. Second, we aligned all high-quality SNPs as defined previously to GRCh38 to create a callset of genetic variation specific to the dataset analysed, and discarded reads with mismatches at these dataset-specific variable sites.

#### Event classification

We classify each candidate read as CO if there is only one transition pair, flanked by at least one SNP on either side (for example, ‘1122’—that is, two SNPs from haplotype 1 followed by two SNPs from haplotype 2); as NCO if there are two transitions (for example, ‘121’); as ‘ambiguous’, indicating that we cannot distinguish CO and NCO, if there is one transition but at least one side without flanking SNPs (for example, ‘12’, ‘112’ or ‘122’); or ‘complex’ if there are more than two switches.

For CO reads, to compare to the background CO recombination rate, we also calculate the average CO recombination rate (cM Mb^−1^) across the interval spanned by the two SNPs between which the CO occurred (between ‘12’ or ‘21’). For the comparison of CO and NCO genetic-length distributions, see Supplementary Methods and Supplementary Fig. 5.

### Probability of observing the same NCO event more than once

We modelled recombinant events as a Poisson process to estimate the probability of observing the same gene conversion more than once. Given that approximately, 30 to 50 CO events and 200 to 1,000 NCO events occur per meiosis and that there are 25,000 to 50,000 meiotic recombination hotspots across the genome, the expected rate of CO and NCO events per hotspot per meiosis (*λ*) is between 0.0006 and 0.002, and 0.004 and 0.04, respectively. Assuming a Poisson distribution, the corresponding ranges of probabilities of observing two or more COs and NCOs within the same hotspot are given by *P*(Poisson *X* ≥ 2) = 1 − e^−*λ*^ (1 + *λ*).

### Mapping to reference genomes

For genetic distances, we used the European male-specific refined genetic map^32^, which is provided in GRCh37 coordinates. To analyse CO rate information, we mapped all reads to the GRCh37 reference genome^62^ using minimap2^55,56^ (v2.26-r1175, with arguments “-ax map-hifi --cs=short --eqx --MD”). For DSB hotspots, the DSB map^37,38^ is provided in GRCh38 coordinates. To analyse DSB data, we similarly used minimap2 to map all reads to the GRCh38 reference genome^63^. To analyse the distance to telomeres, we also mapped all reads to the T2T reference genome^52^.

### Positional bias of converted markers relative to DSB-PRDM9 motif

To test for a positional bias of converted markers relative to the DSB-PRDM9 motif, we calculate the distance between converted markers in NCO reads that overlap a DSB-PRDM9 motif and the centre of the motif, while accounting for the strand on which the motif appears. To test for asymmetry in these distances, we perform a permutation test: we first calculate the fraction of reads where the distance is negative; then, we randomly flip the signs of the distances (equivalent to randomizing the motif strands) and recalculate the fraction of negative randomized distances 10,000 times. The permutation test *P* value is then the fraction of randomizations where the fraction of negative distances was equal to or larger than that of the non-randomized distances.

### PRDM9 motif detection in recombinant reads

PRDM9 motif detection was performed using FIMO^64^ with the PRDM9 A/A position weight matrix provided by Hinch et al.^38^. FIMO was applied to both recombinant reads and a control set of 100,000 randomly sampled CCS reads. Only motif matches with a *q* value < 0.01 were retained for downstream analysis.

### Calculation of gBGC

To calculate gBGC of converted SNPs in a set of NCO reads, we consider only SNPs where one allele is G or C, and the other is A or T. The gBGC is then calculated as the fraction of converted SNPs with G or C out of this subset.

### Tract length distribution inference

We inferred the NCO tract length distribution using an approximate Bayesian computation-like approach. We modelled tracts either as a single-geometric distribution with mean length *L*, or as a mixture of two geometric distributions with proportions *m*:*1 *−* m* and mean lengths *L*_1_ and *L*_2_.

Our fitting procedure was based on three statistics from detected NCOs: (1) the number of converted SNVs; (2) the distance between the first and last converted SNVs (lower bound); and (3) the distance between the flanking SNVs immediately upstream and downstream of the conversion (upper bound).

Given a parameter proposal—either *L* (for the single-geometric model) or *m*, *L*_1_*, L*_2_ (for the mixture model)—we simulated the expected distributions of these statistics. We randomly selected 10.7 million reads, and for each read, we simulated a NCO tract by: (1) drawing a random start position along the read; (2) selecting an geometric component with probability *m* or *1* − *m*; and (3) sampling a tract length from the chosen geometric distribution. Based on the simulated tract, we determined which SNVs would be converted, assessed whether the event would be detectable, and recorded the corresponding number of converted SNVs, lower bound, and upper bound. Reads where the NCO would not be detectable were excluded.

To compare the simulated and observed distributions, we calculated the Kolmogorov–Smirnov two-sample statistic for each of the three statistics and summed these values. We then searched for parameters minimizing this sum. For the mixture model, we first performed a coarse grid search over 11 points for *m* (0.97–1), 11 points for *L*_1_ (10–100 bp) and 21 points for *L*_2_ (200–500 bp). We then refined the search over narrower ranges: 7 points for *m* (0.988–0.994), 31 points for *L*_1_ (20–50 bp) and 31 points for *L*_2_ (650–1,550 bp). For the single-geometric model, we fixed *m* = 1 and searched across 500 values of *L* between 10 and 1,000 bp. To estimate confidence intervals, we generated 100 nonparametric bootstrap samples and repeated the entire fitting procedure for each. The confidence interval for each parameter was defined as the 2.5th to 97.5th percentile of the bootstrap estimates.

### Testing for equality of distributions

We first describe how we test for equality of distribution between two samples. Consider two sets of continuous measurements (for example, recombination rates) for two samples. We use the Anderson–Darling statistic^65^ for two-sample equality of distribution testing, which is based on the integral of the square differences between the empirical cumulative distribution functions of the two measurement sets. To calculate the statistical significance of the observed Anderson–Darling statistic, we perform permutation testing by randomly assigning each measurement to one of the samples, while retaining sample sizes. We permute the data 10,000 times, calculate the Anderson–Darling statistic for each permutation, and record the fraction of permutations for which the Anderson–Darling statistic was larger than the observed Anderson–Darling statistic as our *P* value.

To test for equality of distribution between a set of measurements from one sample, and several sets of measurements from other samples, we first calculate the Anderson–Darling statistic between the focal sample and each other sample, and take their sum as our statistic. To calculate statistical significance, for 10,000 times we perform the permuted assignment as above separately to the focal sample and each of the other samples, and calculate the sum of Anderson–Darling statistics across the randomized sets. Our *P* value is the fraction of (pairwise sequences of) permutations resulting in a statistic larger than the observed one.

### Age effect in blood samples

To test for an association between recombination event rate and sample age, we modelled the number of events per sample as Poisson-distributed with an expected value proportional to sequencing coverage. We compared two models: a null model where the event rate *λ* is constant across samples (expected count = *λ* × coverage), and an alternative model where the rate scales linearly with age (expected count = *λ* × coverage × sample_age). For each model, we optimized the Poisson log likelihood. To evaluate whether age significantly improves model fit, we computed the likelihood ratio statistic comparing the two models. To assess statistical significance, we performed a permutation test. Specifically, we randomly permuted the sample ages across individuals and re-fit the age-dependent Poisson model described above, recording the resulting slope each time. The permutation *P* value was calculated as the fraction of permutations in which the fitted slope exceeded that observed in the original (unpermuted) data.

### Estimating the fraction of non-meiotic NCOs

We estimated the fraction of non-meiotic NCOs using three approaches. First, we approximated the distribution of genetic lengths of NCOs (Fig. 2b) as a mixture of the genetic length distributions of all reads and of COs. Specifically, we maximized the likelihood of the observed NCO lengths (in logarithmic scale) under a *p*:(1 − *p*) mixture of these two distributions, each smoothed by kernel density estimation to enable continuous probability density evaluation. The maximum-likelihood estimate indicated that 45% (95% confidence interval 39.4–51.2%) of NCOs follow the non-meiotic length distribution. We note however that the fit was imperfect, possibly due to model misspecification even if all NCOs originated in meiosis, which may reflect variable preferential resolution of DSBs into COs versus NCOs. Second, since 45% of NCO reads overlap a DSB-PRDM9 motif versus 67% of COs and 13.5% of all reads, we can estimate the fraction of non-meiotic reads of ~41%. Third, similarly we can estimate this fraction as ~32% using the fact that the GC bias of NCOs is 60.7% versus a higher rate of 65.7% at NCO reads overlapping a PRDM9 motif, representing meiotic NCOs, and a background of 50%. We note however that with this method NCOs may not be accurately classified as meiotic, biasing inference.

### Reporting summary

Further information on research design is available in the Nature Portfolio Reporting Summary linked to this article.

## Online content

Any methods, additional references, Nature Portfolio reporting summaries, source data, extended data, supplementary information, acknowledgements, peer review information; details of author contributions and competing interests; and statements of data and code availability are available at https://doi.org/10.1038/s41586-026-10901-0.

## Supplementary information

Supplementary Information Supplementary Methods, Supplementary Tables 1 and 2, Supplementary Figs. 1–6 and Supplementary Notes 1 and 2.

Reporting Summary

Supplementary Table 3 Catalogue of recombination events identified by long-read sequencing. Each row corresponds to a single recombination event and includes the sample and dataset identifiers, event classification (CO, NCO, ambiguous or complex), genomic coordinates, supporting SNP information, gene conversion tract information where applicable, and overlap with PRDM9 motifs and recombination hotspots. A detailed description of all columns is provided in the Legend worksheet.

Peer Review File

## Figures and Tables

**Extended Data Table 1:**
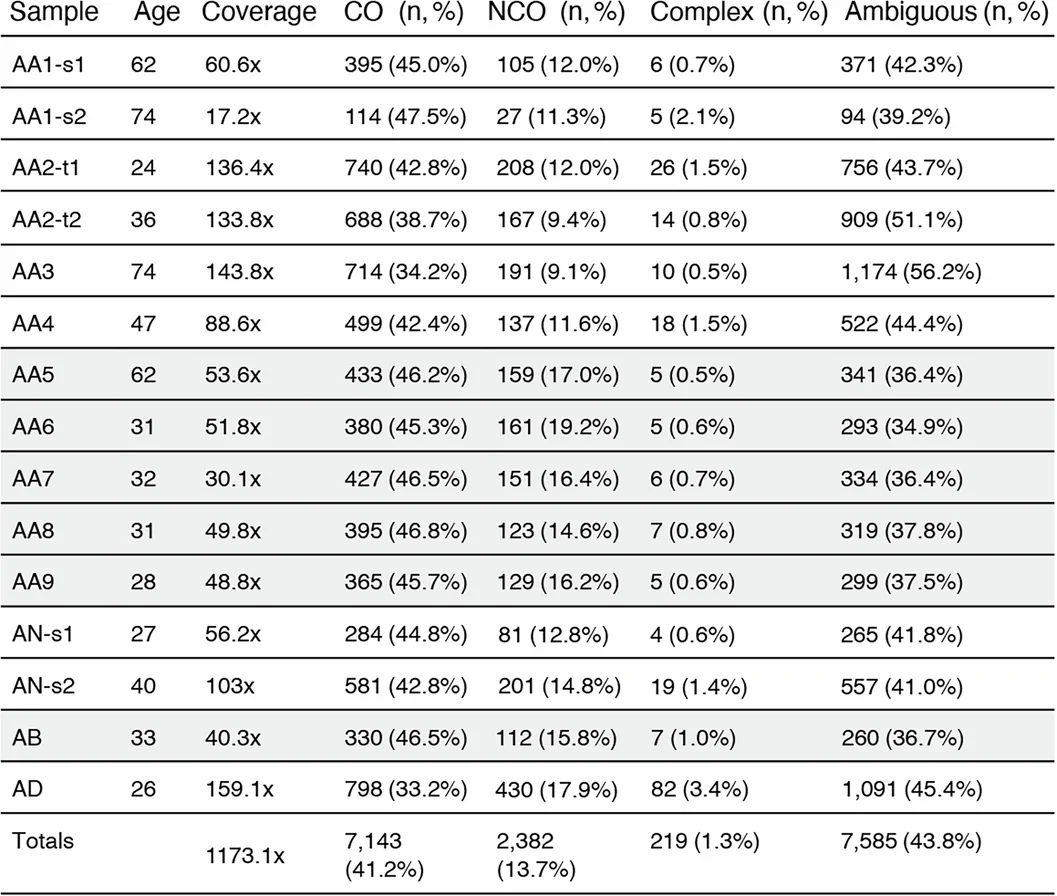
Sperm sample coverage and recombinant event counts

The number of classified recombinant reads with age, sequencing coverage, CO, NCO, ambiguous or complex recombination events, per sample. Sample pairs AA1-s1/AA1-s2 and AN-s1/AN-s2 are from the same donor at different ages. Samples AA2-t1 and AA2-t2 are monozygotic twins. Rows shaded in grey indicate the SL samples sequenced on Revio machines whereas unshaded rows contain TwinsUK samples sequenced on both Sequel II and Revio machines.

**Extended Data Table 2:**
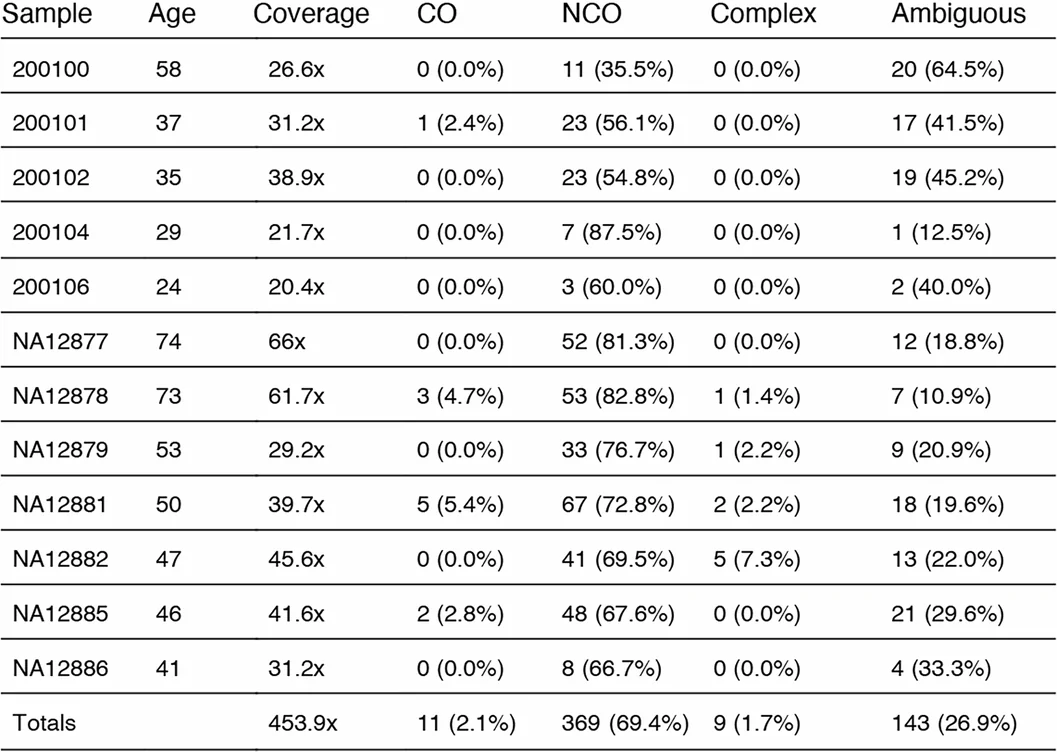
Blood sample coverage and recombinant event counts

The number of classified recombinant reads with age, sequencing coverage, CO, NCO, ambiguous or complex recombination events, per CEPH blood sample. Sequence data requiring demultiplexing was excluded from the analysis, which accounts for the differences in sequencing statistics compared to the original dataset.

## Data Availability

All data relating to TwinsUK samples have been deposited to the TwinsUK BioResource data management team and are available by application to the Twin Research Executive Access committee (TREC) at King’s College London. Researchers can apply for access to individual-level data via the TwinsUK Data Access portal at https://twinsuk.ac.uk/researchers/access-data-and-samples/request-access/. TwinsUK data can be requested for projects related to health, well-being or disease. Median time to decision for data access submissions is three weeks. Please contact V. Vazquez (victoria.vazquez@kcl.ac.uk) for enquiries regarding data access submission. Sequencing data are available via EGA under accession number EGAD00001015736. Sequencing data for SL samples are not permitted to be released for reasons of identifiability. Those wishing to request access to these data should contact P. Sudmant (psudmant@berkeley.edu), who will respond within two weeks. The DeCODE pedigree data used are available at Zenodo (https://doi.org/10.5281/zenodo.14025564 (ref. ^66^)). The Platinum Pedigree data used are available on Amazon Open Data as documented at https://github.com/Platinum-Pedigree-Consortium/Platinum-Pedigree-Datasets or at dbGaP study accession phs003793.v1.p1.

## Code availability

The code used to carry out all analyses and make all plots is available under an MIT licence at https://github.com/regevs/recombination and https://doi.org/10.5281/zenodo.21970165 (ref. ^67^). We used snakemake^68^ v7.32.4 for pipeline management and execution and polars v0.20.13 for large-scale data analysis.
