# Author Correction: Long-read sequencing reveals pre-meiotic gene conversion in sperm

**DOI:** 10.1038/s41586-026-11290-0

**Published:** 2026-10-19

**Authors:** Regev Schweiger, Sangjin Lee, Chenxi Zhou, Tsun-Po Yang, Stacy Li, Rashesh Sanghvi, Matthew Neville, Katie Smith, Kirsty Roberts, Ayrun Nessa, Sam Wadge, Kerrin S. Small, Peter J. Campbell, Kristian Almstrup, Peter H. Sudmant, Raheleh Rahbari, Richard Durbin

**Affiliations:** 1 Department of Human Genetics and Computational Medicine, Gray School of Medical Sciences, Gray Faculty of Medical and Health Sciences, Tel Aviv University, Tel Aviv, Israel; 2 Blavatnik School of Computer Science and AI, Sackler Faculty of Exact Sciences, Tel Aviv University, Tel Aviv, Israel; 3 Department of Genetics, University of Cambridge, Cambridge, UK; 4 Cancer Ageing and Somatic Mutation, Wellcome Sanger Institute, Hinxton, UK; 5 Department of Integrative Biology, University of California, Berkeley, Berkeley, CA USA; 6 Department of Twin Research and Genetic Epidemiology, King’s College London, London, UK; 7 Wellcome–MRC Cambridge Stem Cell Institute, Cambridge Biomedical Campus, Cambridge, UK; 8 Department of Growth and Reproduction, Copenhagen University Hospital–Rigshospitalet, Copenhagen, Denmark; 9 Department of Cellular and Molecular Medicine, Faculty of Health and Medical Sciences, University of Copenhagen, Copenhagen, Denmark; 10 International Center for Research and Research Training in Endocrine Disruption of Male Reproduction and Child Health, University of Copenhagen, Copenhagen, Denmark; 11 Center for Computational Biology, University of California, Berkeley, Berkeley, CA USA

**Keywords:** Genomics, Haplotypes, DNA recombination

Correction to: *Nature* https://doi.org/10.1038/s41586-026-10901-0 Published online 26 August 2026

In the version of the article initially published, several parts of Fig. 4 were missing or incorrect. Figure 4 has now been corrected in the HTML and PDF versions of the article, as seen in Fig. 1 below.

**Fig. 1:**
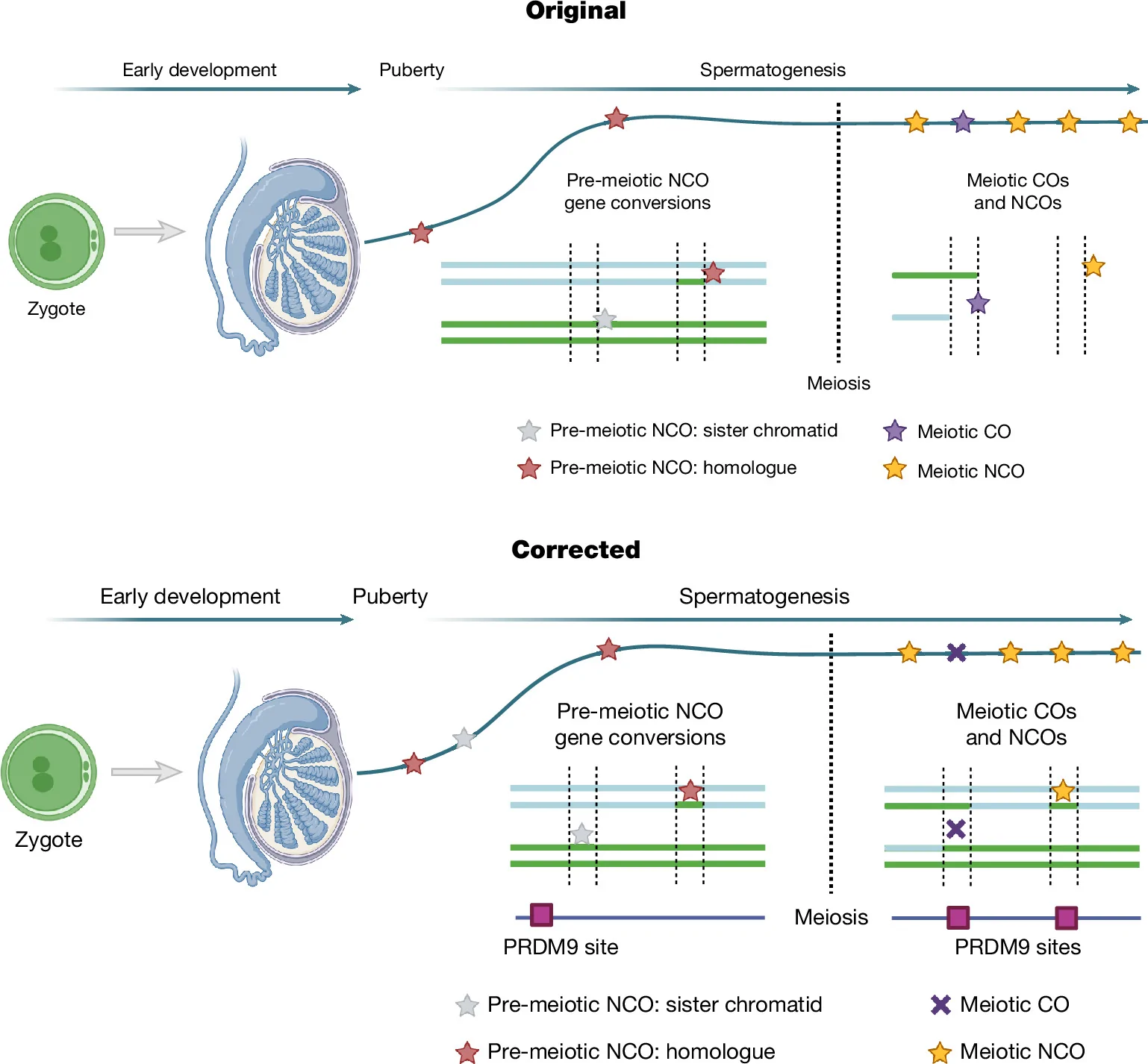
Original and corrected Fig. 4 .

