# Supplementary material for: Long-read sequencing reveals pre-meiotic gene conversion in sperm
Source: Nature. 2026 Aug 26;657(8133):981–7. doi: 10.1038/s41586-026-10901-0 (PMC13600919; doi:10.1038/s41586-026-10901-0)
Supplement: Supplementary file 1 [file 41586_2026_10901_MOESM1_ESM.pdf]

nature portfolio

https://doi.org/10.1038/s41586-026-10901-0

Supplementary information

Long-read sequencing reveals pre-meiotic gene conversion in sperm

In the format provided by the authors and unedited

Supplementary Information for

"*Long-read sequencing reveals pre-meiotic gene conversion in sperm*"

Regev Schweiger, Sangjin Lee, Chenxi Zhou, Tsun-Po Yang, Stacy Li, Rashesh Sanghvi, Matthew Neville, Katie Smith, Kirsty Roberts, Ayrun Nessa, Sam Wadge, Kerrin S Small, Peter J Campbell, Kristian Almstrup, Peter H Sudmant, Raheleh Rahbari* and Richard Durbin*

Correspondence: rd109@cam.ac.uk or rr11@sanger.ac.uk

Supplementary Methods **1**
Calibration of read trimming 1
Calibration of base quality scores 2
Testing for differences in CO and NCO event distributions 3
Supplementary Tables **6**
Supplementary Table 1. Sperm samples. 6
Supplementary Table 2: Blood samples. 7
Supplementary Figures **8**
Supplementary Figure 1. 8
Supplementary Figure 2. 9
Supplementary Figure 3. 10
Supplementary Figure 4. 11
Supplementary Figure 5. 12
Supplementary Figure 6. 13
Supplementary Notes **14**
Supplementary Note 1: Sequence of the new PRDM9 allele 14
Supplementary Note 2: Estimates of pre-meiotic gene conversions in sperm 15

Supplementary Methods

Calibration of read trimming

For the subset of reads of minimal length 5kbp, we counted the number of 1bp segments which are mismatches to both haplotypes, as a function of the distance to read end (<5kbp, bins of 10bp). Since some of these mismatches are a result of assembly errors, we further limited our analysis to high confidence 1bp mismatches with; at least 10bp of matched alignment on both sides of the segment; do not overlap a low complexity or a repeat (Methods); and with a BQ of 30 or more (we need only choose a reasonable BQ level, since for this analysis we only care about relative error rates between read end distances).

For the TwinsUK samples, sequenced by the Sequel II instrument, we observed a slow decay of error rates until a distance of 2-3kbp from read end (**Supplementary Fig. 1**). We selected a threshold of 1,500bp for detection SNPs (that is, SNPs within 1,500bp of read ends would not be used to detect recombination events), and a less stringent threshold of 500bp for classification SNPs (used to classify events, once detected).

For the SL samples, sequenced by the Revio instrument, we observed a substantially lower baseline error rate, as well as a faster drop from read ends to the baseline (**Supplementary Fig. 2**). We therefore used a threshold of 400bp for detection SNPs and 200 for classification SNPs.

Calibration of base quality scores

To calibrate the base quality (BQ) scores, we make use of the high coverage of reads to estimate whether a mismatch to a haplotype is a gene conversion or a sequencing error, and therefore get an estimate of false positive NCO calls. In more detail, we analyse SNPs - 1bp segments that match one haplotype but mismatch the other; that are not within 1,500bp of the read end; have at least 10bp of matched alignment on both sides of the segment; do not overlap a low complexity region or a repeat (see Methods). We further limit our analysis to reads where the 1bp segment mismatch is surrounded by at least 3 SNPs which do match the haplotype (i.e. a pattern of "...1112111...").

These 1bp SNPs can be caused either by NCO leading to gene conversion; by a sequencing error, or by de-novo mutations. Of these, the latter is rare (a rate of 10^{-8}/bp), so these SNPs are likely to originate from either an NCO or a sequencing error. To distinguish between these two scenarios, we compare the SNP to the other haplotype. If this is a sequencing error, there is no particular reason that it should flip to the other allele vs. any other nucleotide, apart from the particular sequencing error patterns. Conversely, if this is a true GC, then it should always flip to the other allele. Therefore, the ratio between the number of SNPs that flip to a nucleotide different than the

two haplotypes, and the number of SNPs that flip to the allele of the other haplotype, is a proxy of the false positive call rate we expect for NCOs.

For Sequel II data (**Supplementary Fig. 3**), we set the BQ threshold for detection SNPs to be 60, and 30 for classification SNPs. For Revio data (**Supplementary Fig. 4**), the BQ threshold for detection SNPs was 40 and 30 for classification SNPs.

Testing for differences in CO and NCO event distributions

Problem setup

We would like to test if the spatial distribution of CO rates along the genome is different from that of NCO events. The direct way to do this would be to observe a large number of both CO and NCO events everywhere along the genome and compare them at each location. However, this would require unrealistically high coverage, many thousands if not millions of times higher than we have. Therefore, instead we group together reads that are expected to behave similarly in terms of recombination and detection probabilities, and then test for differences between groups of CO reads and NCO reads, as we describe below. The CO recombination map is a natural proxy for such grouping, as CO rates are expected to follow it closely.

We first limit ourselves to reads (in addition to QC described above) with at least 5 SNPs, and we define the genomic interval between the second SNP and the second-to-last SNP as the *detectable interval*. We will only be interested in CO reads where the recombination occurs in the detectable interval, i.e. the first two (or more) SNPs belong to one haplotype and the last two (or more) SNPs to other ("11...22"). Similarly, we will only be interested in NCO reads where the converted SNP/s are in the detectable interval, i.e. the first and last two SNPs are not converted ("11...2...11"). This increases the confidence of correct classification while dealing with the same set of SNPs for both types of events.

Our null model assumes that (a) both CO and NCO events are initiated by a DSB, which is initiated according to some spatial distribution along the genome; (b) each DSB site is resolved to a CO at probability q and to an NCO at probability $1-q$, independent and uniformly along the genome; (c) COs occurring in the detectable interval are always detected, while NCOs will only be detected if a marker within the detectable interval is converted; (d) NCO tract lengths are geometrically distributed with a known mean length, and therefore, given the pattern of markers on a read, we can calculate the probability of detection. Note that for this analysis we assume a single geometric distribution for tract lengths and not a mixture, for simplicity.

Formally, we use the following graphical model (illustrated in **Supplementary Fig. 5**) as our null model:

1. The genetic length L_i of a detectable interval is sampled from a random variable L.
2. A recombination event occurs in the detectable interval ($R_i=1$) according to a probability $u(L_i)$ determined by the genetic length L_i.
3. The type $T_i \in \{CO, NCO, None\}$ of the recombination event is determined independently for each read conditional on R_i; if $R_i=1$ then it resolves to a CO with probability q, and to a NCO with probability $1\text{-}q$; and if $R_i=0$ it is always *None*. The SNP pattern P_i, which consists of the number of SNPs and their distances along the detectable interval, is sampled from a random variable P, which in general is not independent of L.
4. The random variable $D \in \{CO, NCO, None\}$ determines if and which type of recombination event was detected. If no event occurred ($R_i = 0$ and $T_i = None$), then also $D_i = None$. If a CO occurred ($T_i = CO$) at the detectable interval, then it is always detected and $D_i = CO$. If a NCO occurs ($T_i = NCO$), then it is detected in a probability f_i determined only by the SNP pattern $f_i = f(P_i)$.
5. If the NCO tract lengths are geometrically distributed with a known mean tract length, we can calculate f_i explicitly according to the likelihood model described in the main text.
6. *L, P* and *D* are observed, the rest of the variables are not.

In terms of this graphical model, the assumption that the decision to resolve into CO or NCO is independent of the genetic coordinates translates into $T \perp L \mid R$ (i.e. *T* is independent of *L*, conditional on *R*; indicated in the diagram as T just depending on R). However, we do not observe *T* directly, only via *D*, which is affected by *L* via *P*.

Test: Assuming independence between cM rates and SNP patterns

The simplest assumption to make is that the probability of recombination in a candidate interval $u(l)$ is independent of the chance of an NCO being detected in that interval if it happened, $\sum_p f(p)\, Pr\,(p)$. In terms of the graphical model, we assume here $P \perp L$. In this case, we can decompose:

$$Pr(D = CO \mid L = l) = \sum_{r,t} Pr(D = CO \mid T = t) \cdot Pr(T = t \mid R = r) \cdot Pr(R = r \mid L = l)$$

Since a CO will only be detected if has occurred, this is:

$$= Pr(D = CO|T = CO) \cdot Pr(T = CO \mid R = 1) \cdot Pr(R = 1|L = l) = 1 \cdot q \cdot u(l)$$

Similarly for NCOs,

$$Pr(D = NCO \mid L = l) = \sum_{r,t,p} Pr(D = NCO \mid t, p) \cdot Pr(p) \cdot Pr(t \mid r) \cdot Pr(r \mid l)$$

$$= (1 - q)u(l) \cdot \sum_{p} f(p)\, Pr(p)$$

Using Bayes' law,

$$Pr(L = l|D = CO) = Pr(D = CO \mid L = l) \cdot Pr(L = l)/Pr(D = CO) \propto u(l)Pr(L = l)$$
$$Pr(L = l|D = NCO) = Pr(D = NCO \mid L = l) \cdot Pr(L = l)/Pr(D = NCO) \propto u(l)Pr(L = l)$$

It follows that these two conditional distributions are identical, and can therefore be tested with a two-sample test for equality of 1D distributions, e.g Kolmogorov-Smirnov or Anderson-Darling.

Because we observe L and P directly, and f is a function of P, we can examine whether the assumption of L and f being independent is reasonable. They are indeed significantly correlated, but very weakly (Pearson's r=0.02, p<1e-10). Furthermore, the correlation is positive, which would make NCOs more detectable for higher values of L, whereas we see in **Fig. 2** that NCOs are relatively less detected at higher values of L. The slight positive correlation may be due to the mutagenic effect of recombination which may lead to a positive correlation between cM rates and SNP density.

Supplementary Tables

Sample	Source ID	Dataset	Age	# of CCS reads	Read length ± std	Read depth	# of Sequel II/Revio flow cells
AA1-s1	PD50508b	TwinsUK	62	14,366,105	12,647 ± 4,536	60.58	2/2
AA1-s2	PD50508f	TwinsUK	74	3,040,994	17,011 ± 3,308	17.25	2/0
AA2-t1	PD46180c	TwinsUK	24	29,375,470	13,929 ± 5,568	136.38	3/4
AA2-t2	PD50519d	TwinsUK	36	33,329,411	12,047 ± 5,315	133.84	3/4
AA3	PD50477f	TwinsUK	74	38,422,720	11,232 ± 4,354	143.85	3/4
AA4	PD50489e	TwinsUK	47	25,190,427	10,548 ± 5,358	88.58	4/3
AA5	1894	SL	62	9,181,559	17,527 ± 4,909	53.64	0/2
AA6	3898	SL	31	8,634,738	17,985 ± 4,982	51.76	0/2
AA7	7899	SL	32	5,367,893	16,846 ± 4,671	30.14	0/1
AA8	95619	SL	31	8,876,456	16,835 ± 5,001	49.81	0/2
AA9	115980	SL	28	9,037,595	16,197 ± 4,788	48.79	0/2
AN-s1	PD50521b	TwinsUK	27	11,618,202	14,522 ± 4,111	56.24	4/1
AN-s2	PD50521e	TwinsUK	40	28,445,892	10,863 ± 4,401	103.02	4/3
AB	2895	SL	33	6,982,155	17,318 ± 4,406	40.30	0/2
AD	PD50523b	TwinsUK	26	35,591,943	13,413 ± 4,821	23.69	4/5

Supplementary Table 1. Sperm samples.

The Source ID is used in database submissions. Sample pairs AA1-s1/AA1-s2 and AN-s1/AN-s2 are from the same donor at different ages. Samples AA2-t1 and AA2-t2 are monozygotic twins.

Sample ID	Age	# of CCS reads	Read length ± std	Read depth	# of Sequel II flow cells	# of Revio flow cells
200100	58	6,619,579	12,059 ± 4,021	26.61	0	1
200101	37	9,188,613	10,183 ± 4,541	31.20	0	2
200102	35	9,387,139	12,443 ± 5,279	38.94	0	2
200104	29	5,045,933	12,890 ± 5,513	21.68	0	1
200106	24	4,635,172	13,215 ± 4,344	20.42	0	1
NA12877	74	11,128,682	17,804 ± 3,246	66.04	9	0
NA12878	73	11,480,931	16,119 ± 3,558	61.69	8	0
NA12879	53	4,933,624	17,756 ± 4,595	29.21	5	1
NA12881	50	5,187,297	22,961 ± 4,870	39.71	0	4
NA12882	47	9,123,387	14,996 ± 5,110	45.61	0	2
NA12885	46	11,006,516	11,331 ± 4,179	41.57	0	2
NA12886	41	6,306,752	14,866 ± 5,469	31.25	0	2

Supplementary Table 2: Blood samples.

Supplementary Figures

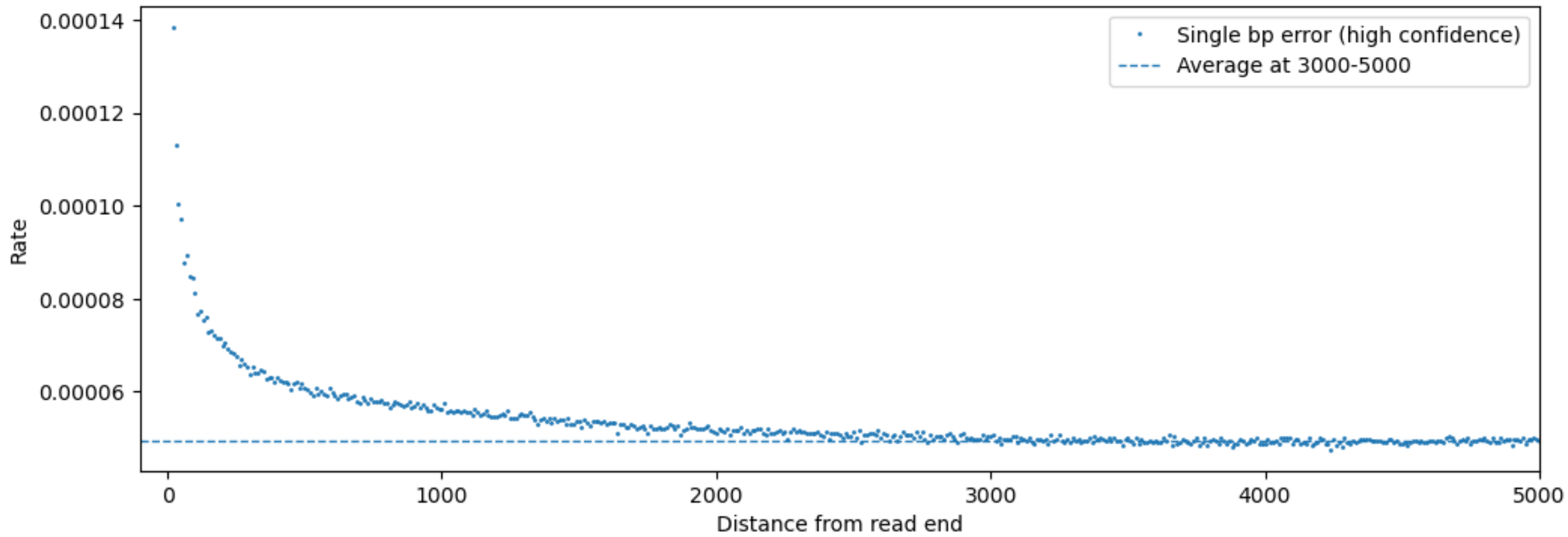

Supplementary Figure 1.

Relative rates of high confidence single-bp mismatches, as a function of distance to read ends, for the Sequel II instrument.

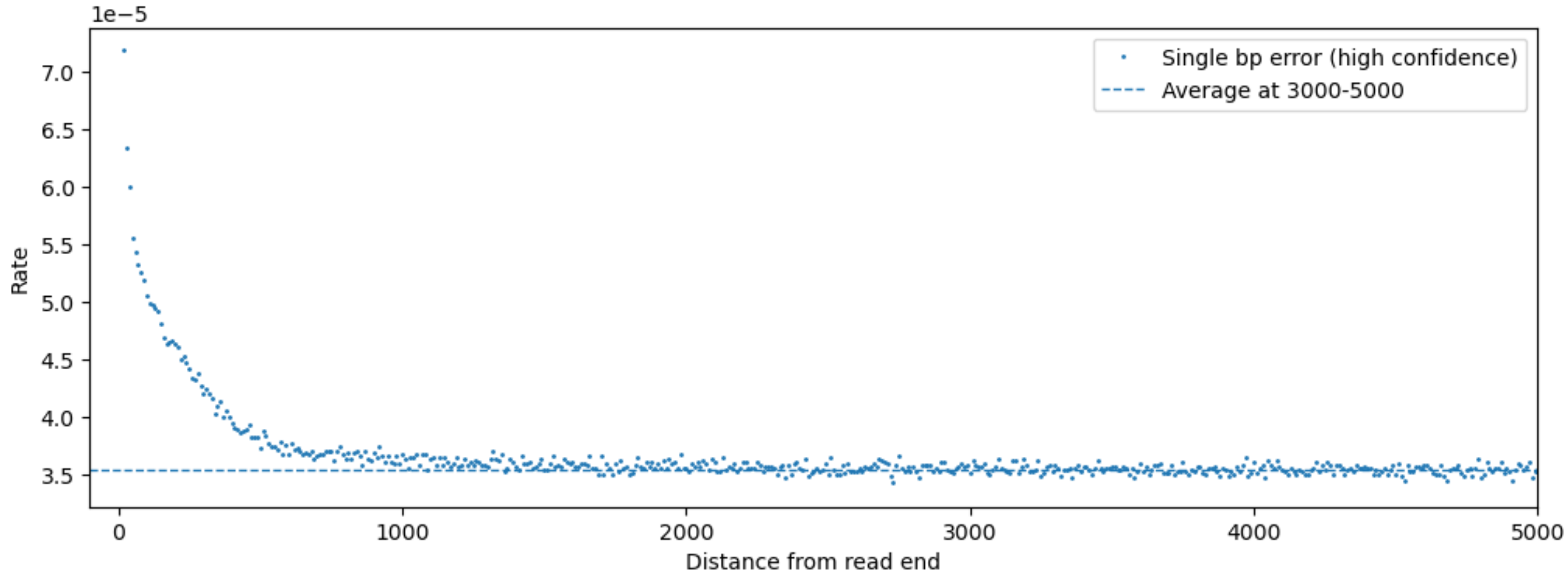

Supplementary Figure 2.

Relative rates of high confidence single-bp mismatches, as a function of distance to read ends, for the Revio instrument.

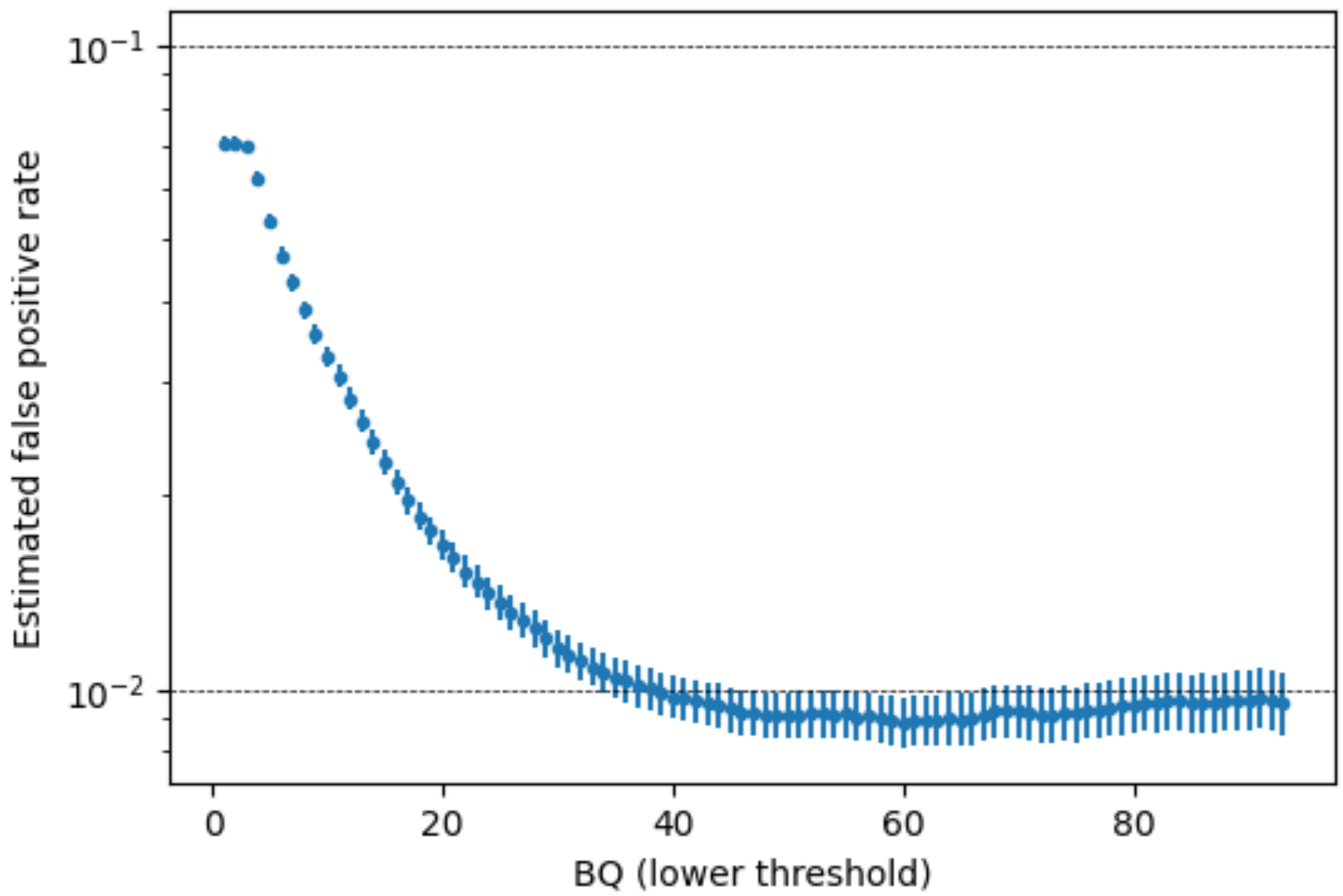

Supplementary Figure 3.

An estimate of the rate of false positive NCO calls, as a function of base quality (BQ) minimal threshold, for Sequel II data.

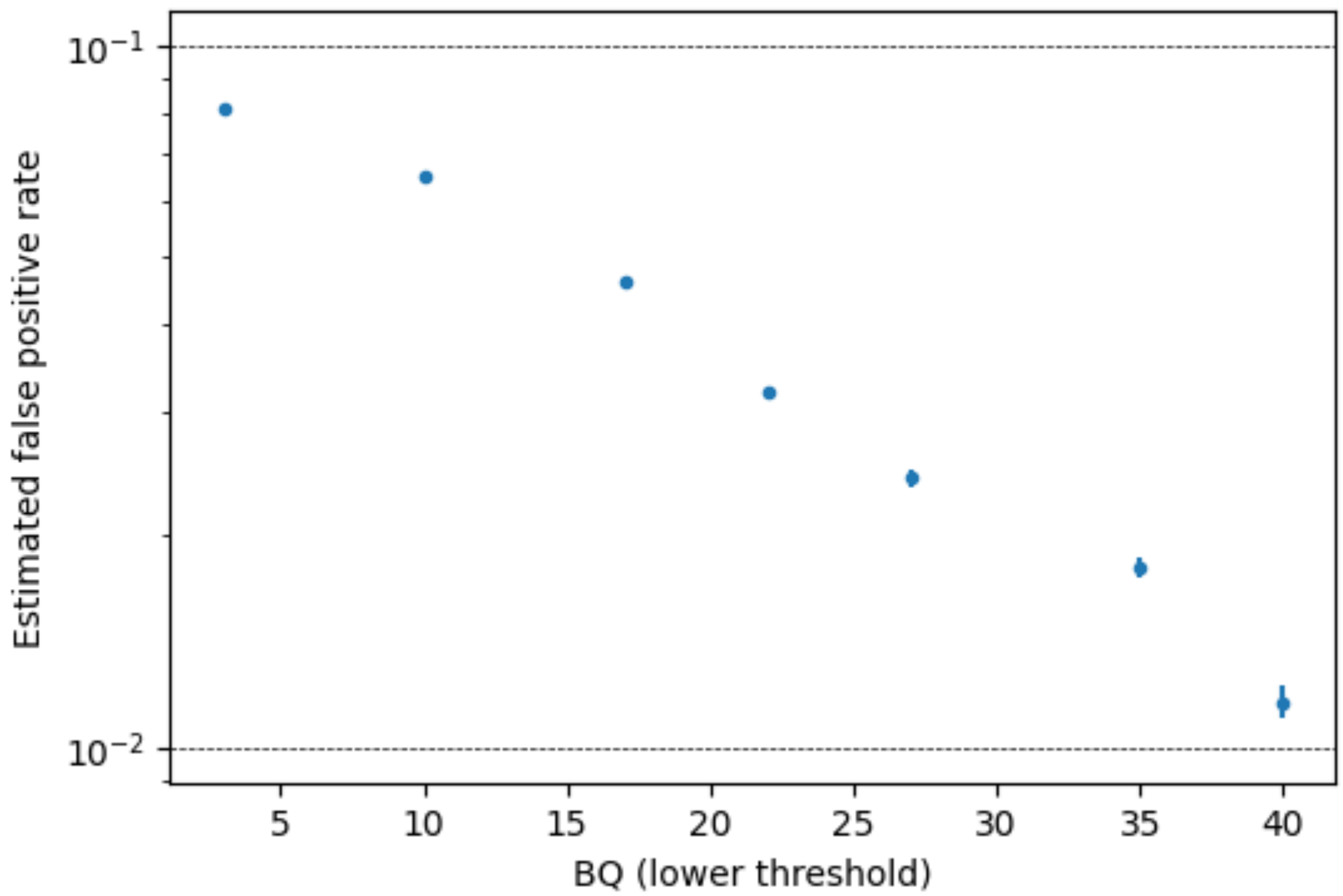

Supplementary Figure 4.

An estimate of the rate of false positive NCO calls, as a function of base quality (BQ) minimal threshold, for Revio data.

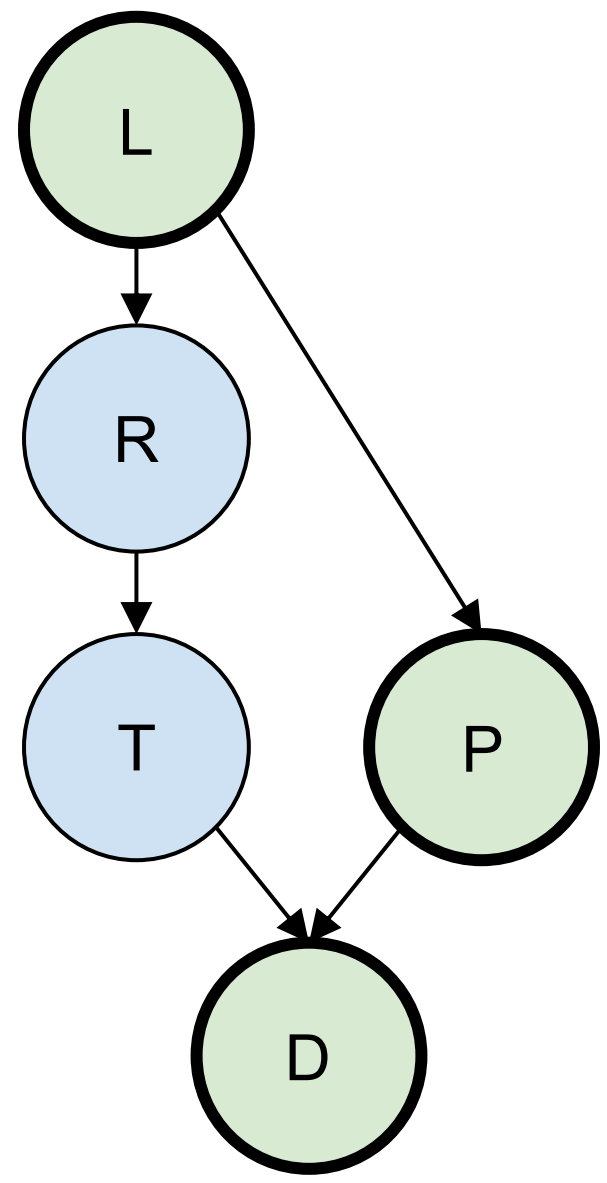

Supplementary Figure 5.

The graphical model underlying the test for differences between COs and NCOs. The genetic length (*L*) is sampled from a random variable; a recombination event occurs (*R*) in probability determined by *L*; the type (*T*) of recombination event is determined; then this event is detected (*D*) in probability determined by its type *T* and the SNP patterns on the read (*P*).

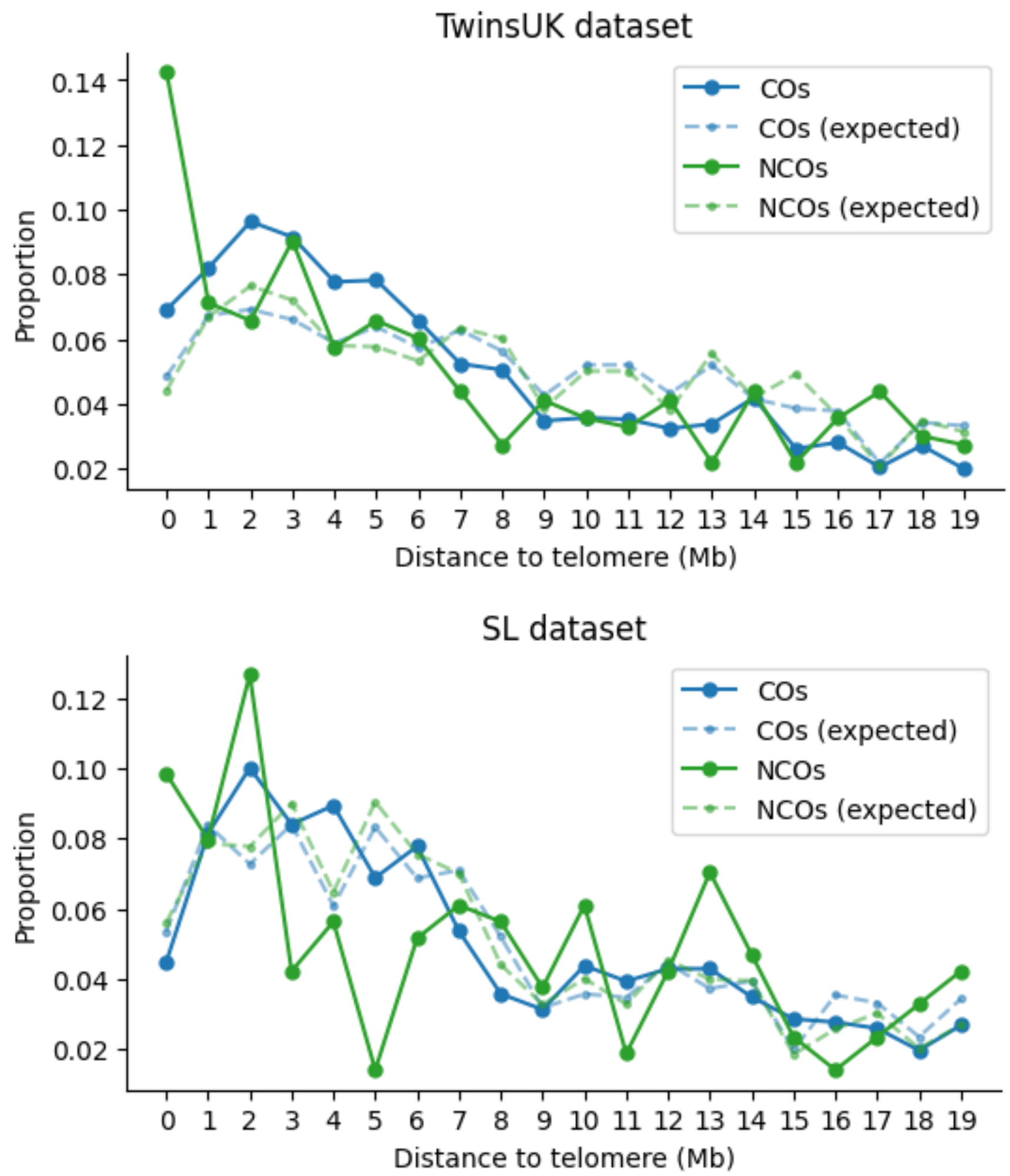

Supplementary Figure 6.

The distribution of distances of CO and NCO events from telomeres, stratified by dataset (TwinsUK and SL).

Supplementary Notes

Supplementary Note 1: Sequence of the new PRDM9 allele

TGTGGACAAGGTTTCAGTGTTAAATCAGATGTTATTACACACCAAAGGACACATACAGGGGAGAAGCTCT
ACGTCTGCAGGGAGTGTGGGCGGGGCTTTAGCTGGAAGTCACACCTCCTCATTCACCAGAGGATACACAC
AGGGGAGAAGCCCTATGTCTGCAGGGAGTGTGGGCGGGGCTTTAGCTGGCAGTCAGTCCTCCTCACTCAC
CAGAGGACACACACAGGGGAGAAGCCCTATGTCTGCAGGGAGTGTGGGCGGGGCTTTAGCTGGCAGTCAG
TCCTCCTCACTCACCAGAGGACACACACAGGGGAGAAGCCCTATGTCTGCAGGGAGTGTGGGCGGGGCTT
TAGCCGGCAGTCAGTCCTCCTCACTCACCAGAGGAGACACACAGGGGAGAAGCCCTATGTCTGCAGGGAG
TGTGGGCGGGGCTTTAGCCGGCAGTCAGTCCTCCTCACTCACCAGAGGAGACACACAGGGGAGAAGCCCT
ATGTCTGCAGGGAGTGTGGGCGGGGCTTTAGCTGGCAGTCAGTCCTCCTCAGTCACCAGAGGACACACAC
AGGGGAGAAGCCCTATGTCTGCAGGGAGTGTGGGCGGGGCTTTAGCTGGCAGTCAGTCCTCCTCACTCAC
CAGAGGACACACACAGGGGAGAAGCCCTATGTCTGCAGGGAGTGTGGGCGGGGCTTTAGCAATAAGTCAC
ACCTCCTCAGACACCAGAGGACACACACAGGGGAGAAGCCCTATGTCTGCAGGGAGTGTGGGCGGGGCTT
TCGCGATAAGTCACACCTCCTCAGACACCAGAGGACACACACAGGGGAGAAGCCCTATGTCTGCAGGGAG
TGTGGGCGGGGCTTTAGAGATAAGTCAAACCTCCTCAGTCACCAGAGGACACACACAGGGGAGAAGCCCT
ATGTCTGCAGGGAGTGTGGGCGGGGCTTTAGCAATAAGTCACACCTCCTCAGACACCAGAGGACACACAC
AGGGGAGAAGCCCTATGTCTGCAGGGAGTGTGGGCGGGGCTTTCGCAATAAGTCACACCTCCTCAGACAC
CAGAGGACACACACAGGGGAGAAGCCCTACGTCTGCAGGGAGTGTGGGCGGGGCTTTAGCGATAGGTCAA
GCCTCTGCTATCACCAGAGGACACACACAGGGGAGAAGCCCTACGTCTGCAGGGAG

Supplementary Note 2: Estimates of pre-meiotic gene conversions in sperm

We provide here an explicit calculation, based on published estimates of germline mitotic divisions, DSB rates, and inter-homologue repair frequencies, to evaluate whether the observed numbers of pre-meiotic GCs in sperm sequencing are theoretically achievable.

First, a review of the origins and patterns of human mutation by Crow[44] (Nat Rev Genet, 2000) estimates approximately 30 mitotic cell divisions in the male germline by age 15, followed by roughly 23 additional divisions per year thereafter, corresponding to approximately one stem-cell division every 16 days. Given that the mean age of donors in our dataset is ~42 years, this implies an expected value in the range of ~600–700 divisions. (Across the observed donor age span of 26 to 74 years, the corresponding number of pre-meiotic divisions ranges more broadly from roughly 300 to 1,400.)

A review by Bouwman and Crosetto[45] (Genes, 2018) states that "the frequency of DSBs is estimated to be 10–50 events per cell per day." Similarly, Vilenchik and Knudson[46] (PNAS, 2003) write: "We estimate that in normal human cells ≈1% of single-strand lesions are converted to ≈50 endogenous DSBs per cell per cell cycle." Although the precise number depends on cell type and state, these estimates consistently indicate the same order of magnitude. A conservative working range of 10–50 DSBs per cell cycle is therefore well supported.

Stinson and Loparo[47] (Annu Rev Biochem, 2021) state that "NHEJ is the predominant pathway in human cells and repairs up to ~80% of all DSBs," and Shibata et al.[48] (EMBO J, 2011) report that "NHEJ represents the major DSB repair pathway in G2 as in G1 with ∼75–85% of IR-induced DSBs undergoing repair by NHEJ." These place NHEJ in the ~75–85% range in mitotic human cells, with homologous recombination accounting for the remaining ~15–25% of DSB repair events. A working estimate of 15–25% HR is therefore supported.

Moynahan and Jasin[34] (Nat Rev Mol Cell Biol, 2010) note the "low overall frequency of inter-homologue HR (1 in 100 HR events)" in mitotic cells, indicating that only ~1% of homologous recombination events use the homologous chromosome as a repair template rather than the sister chromatid.

It is well established (e.g. reviewed in Sung, P., Klein, H.[31] Nat Rev Mol Cell Biol, 2006) that in mitotically dividing cells, HR overwhelmingly resolves DSBs as non-crossover gene conversion. This strong bias reflects active crossover-suppression mechanisms during mitotic repair, including dissolution of recombination intermediates by the BLM helicase complex. Consistent with this,

our blood-based analyses similarly detect nearly zero crossover events, supporting the conclusion that >99% of mitotic recombination outcomes are non-crossover gene conversions.

Finally, based on our own calculations (as described in the manuscript) and explicitly accounting for the short tract lengths and marker density, we estimate that only ~1–2% of gene conversions would convert at least one SNP and thus be detectable.

Combining these ranges yields a straightforward quantitative bound: For a mean donor age of ~42 years, we expect ~600–700 pre-meiotic mitotic divisions per lineage (broader range ~300–1,400). Assuming 10–50 DSBs per division gives ~6,000–35,000 total DSBs per lineage (up to ~70,000 at the extreme). With ~15–25% repaired by HR, this corresponds to ~900–8,750 HR events, of which ~1% involve the homologous chromosome, yielding ~9–88 inter-homologue gene conversions per lineage. Accounting for our estimated 1–2% detectability given short tract lengths and marker density, this translates to an expected ~0.1–1.8 detectable inter-homologue GCs per sperm lineage, with central parameter choices near ~0.5–1 event. In our data, at a total coverage of 1173.1× we observe 2,382 non-crossovers; if ~30% of these are pre-meiotic ("mitotic"), this corresponds to ~0.6 detected mitotic non-crossovers per haploid genome, well within the predicted range and definitely within the correct order of magnitude.
