# Supplementary material for: Long-read sequencing reveals pre-meiotic gene conversion in sperm
Source: Nature. 2026 Aug 26;657(8133):981–7. doi: 10.1038/s41586-026-10901-0 (PMC13600919; doi:10.1038/s41586-026-10901-0)
Supplement: Supplementary file 2 [file 41586_2026_10901_MOESM2_ESM.pdf]

Corresponding author(s): Richard Durbin, Raheleh Rahbari

Last updated by author(s): 6/03/2026

Reporting Summary

Nature Portfolio wishes to improve the reproducibility of the work that we publish. This form provides structure for consistency and transparency in reporting. For further information on Nature Portfolio policies, see our Editorial Policies and the Editorial Policy Checklist.

Statistics

For all statistical analyses, confirm that the following items are present in the figure legend, table legend, main text, or Methods section.

n/a	Confirmed	
☐	☒	The exact sample size (*n*) for each experimental group/condition, given as a discrete number and unit of measurement
☐	☒	A statement on whether measurements were taken from distinct samples or whether the same sample was measured repeatedly
☐	☒	The statistical test(s) used AND whether they are one- or two-sided<br>*Only common tests should be described solely by name; describe more complex techniques in the Methods section.*
☐	☒	A description of all covariates tested
☐	☒	A description of any assumptions or corrections, such as tests of normality and adjustment for multiple comparisons
☐	☒	A full description of the statistical parameters including central tendency (e.g. means) or other basic estimates (e.g. regression coefficient) AND variation (e.g. standard deviation) or associated estimates of uncertainty (e.g. confidence intervals)
☐	☒	For null hypothesis testing, the test statistic (e.g. *F*, *t*, *r*) with confidence intervals, effect sizes, degrees of freedom and *P* value noted<br>*Give P values as exact values whenever suitable.*
☒	☐	For Bayesian analysis, information on the choice of priors and Markov chain Monte Carlo settings
☐	☒	For hierarchical and complex designs, identification of the appropriate level for tests and full reporting of outcomes
☐	☒	Estimates of effect sizes (e.g. Cohen's *d*, Pearson's *r*), indicating how they were calculated

Our web collection on statistics for biologists contains articles on many of the points above.

Software and code

Policy information about availability of computer code

Data collection	No code was used to collect data in this study - code was only used to analyse data.
Data analysis	All code used to analyse the data is available at https://github.com/ regevs/recombination and DOI 10.5281/zenodo.20533554.<br>Additional publicly available code was used:<br>snakemake - version 7.32.4<br>hifiasm - version 0.20.0-r639<br>minimap2 - version 2.26-r1175<br>bcftools - version 1.18<br>sdust - version 0.1-r2<br>pandas - version 2.2.3<br>scipy - version 1.14.1<br>scikit-learn - version 1.6.1<br>matplotlib - version 3.9.2<br>pysam - version 0.23.0<br>pyBigWig - version 0.3.23<br>pyliftover - version 0.4.1<br>msprime - version 1.3.3<br>igv-notebook - version 0.5.2<br>Python - version 3.11.10<br>RagTag - version 2.1.0<br>samtools - version 1.19

bedtools - version 2.31.0
Tandem Repeat Finder - version 4.09.1
numpy - version 2.0.2
statsmodels - version 0.14.4
polars - version 1.14.0
seaborn - version 0.13.2
pybedtools - version 0.10.0
pyreadr - version 0.5.3
Biopython - version 1.85
numba - version 0.60.0

For manuscripts utilizing custom algorithms or software that are central to the research but not yet described in published literature, software must be made available to editors and reviewers. We strongly encourage code deposition in a community repository (e.g. GitHub). See the Nature Portfolio guidelines for submitting code & software for further information.

Data

Policy information about availability of data

All manuscripts must include a data availability statement. This statement should provide the following information, where applicable:

- Accession codes, unique identifiers, or web links for publicly available datasets
- A description of any restrictions on data availability
- For clinical datasets or third party data, please ensure that the statement adheres to our policy

All data relating to TwinsUK samples have been deposited to the TwinsUK BioResource data management team and are available by application to the Twin Research Executive Access committee (TREC) at King's College London. Researchers can apply for access to individual level data via the TwinsUK Data Access portal at https://twinsuk.ac.uk/researchers/access-data-and-samples/request-access/. TwinsUK data can be requested for projects related to health, well-being or disease. Median time to decision for data access submissions is three weeks. Please contact Victoria Vazquez (victoria.vazquez@kcl.ac.uk) for enquiries regarding data access submission. Sequencing data are available via EGA under accession number EGAD00001015736. The DeCODE pedigree data used are available at Zenodo (https://doi.org/10.5281/zenodo.14025564). The Platinum Pedigree data used are available on Amazon Open Data as documented at https://github.com/Platinum-Pedigree-Consortium/Platinum-Pedigree-Datasets or at dbGaP Study Accession: phs003793.v1.p1.

Research involving human participants, their data, or biological material

Policy information about studies with human participants or human data. See also policy information about sex, gender (identity/presentation), and sexual orientation and race, ethnicity and racism.

Reporting on sex and gender	Most analyses was done on sperm samples and hence those findings apply to males. Blood samples from the open access Platinum Genomes pedigree were reported using published identifiers linked to sex. Reanalysis of data from deCODE uses their published results aggregated by them by sex.
Reporting on race, ethnicity, or other socially relevant groupings	We have used self reported ancestry in this manuscript. All donors in the study self-reported European ancestry.
Population characteristics	The sperm samples were obtained from 13 donors, with ages at donation varying between 24 and 74. Two of these individuals were monozygotic twins.
Recruitment	The TwinsUK cohort comprises research volunteers aged 18 to 100, with approximately equal numbers of identical (MZ) and non-identical (DZ) twins. Recruitment is carried out by the Department of Twin Research at King's College London.
Ethics oversight	The work reported on TwinsUK samples was carried out with informed consent under TwinsUK BioBank ethics, approved by the North West–Liverpool Central Research Ethics Committee (REC reference 19/NW/0187), IRAS ID 258513 and earlier approvals granted to TwinsUK by the St Thomas' Hospital Research Ethics Committee, later the London–Westminster Research Ethics Committee (REC reference EC04/015). SL samples were obtained from Discovery Life Sciences as discarded medical waste in accordance with IRB protocol DLS-BB050. These were collected as part of standard-of-care testing ordered by the patient's physician and excess material released for research.

Note that full information on the approval of the study protocol must also be provided in the manuscript.

Field-specific reporting

Please select the one below that is the best fit for your research. If you are not sure, read the appropriate sections before making your selection.

☒ Life sciences ☐ Behavioural & social sciences ☐ Ecological, evolutionary & environmental sciences

For a reference copy of the document with all sections, see nature.com/documents/nr-reporting-summary-flat.pdf

Life sciences study design

All studies must disclose on these points even when the disclosure is negative.

Sample size	The samples were selected based on availability from the TwinsUK cohort.
Data exclusions	2 TwinsUK samples were excluded because preliminary QC indicated likely sample mixture, and 3 SL 3 samples were excluded because of problematic assembly or phasing, possible contamination, or unexpectedly low coverage - these criteria were not pre-established. 4 samples from the CEPH dataset were excluded because they were from immortalized cell-lines rather than primary blood - this was pre-established. We also excluded Platinum Pedigree read sets that had been demultiplexed, which showed signs of cross-contamination - this was not pre-established.
Replication	Our manuscript describes the landscape of crossover and non-crossover events in the sperm samples. It was replicated across three separate sperm data sets, two sequenced by us and one published by Porsborg et al. Nature Comms (2025), and also in pedigree data from deCODE.
Randomization	Our manuscript describes the landscape of crossover and non-crossover events. It does not test a treatment, and so randomization does not apply.
Blinding	Our manuscript describes the landscape of crossover and non-crossover events. It does not test a treatment, and so blinding does not apply.

Reporting for specific materials, systems and methods

We require information from authors about some types of materials, experimental systems and methods used in many studies. Here, indicate whether each material, system or method listed is relevant to your study. If you are not sure if a list item applies to your research, read the appropriate section before selecting a response.

Materials & experimental systems

n/a	Involved in the study	
☒	☐	Antibodies
☒	☐	Eukaryotic cell lines
☒	☐	Palaeontology and archaeology
☒	☐	Animals and other organisms
☒	☐	Clinical data
☒	☐	Dual use research of concern
☒	☐	Plants

Methods

n/a	Involved in the study	
☒	☐	ChIP-seq
☒	☐	Flow cytometry
☒	☐	MRI-based neuroimaging

Plants

Seed stocks	*Report on the source of all seed stocks or other plant material used. If applicable, state the seed stock centre and catalogue number. If plant specimens were collected from the field, describe the collection location, date and sampling procedures.*
Novel plant genotypes	*Describe the methods by which all novel plant genotypes were produced. This includes those generated by transgenic approaches, gene editing, chemical/radiation-based mutagenesis and hybridization. For transgenic lines, describe the transformation method, the number of independent lines analyzed and the generation upon which experiments were performed. For gene-edited lines, describe the editor used, the endogenous sequence targeted for editing, the targeting guide RNA sequence (if applicable) and how the editor was applied.*
Authentication	*Describe any authentication procedures for each seed stock used or novel genotype generated. Describe any experiments used to assess the effect of a mutation and, where applicable, how potential secondary effects (e.g. second site T-DNA insertions, mosiacism, off-target gene editing) were examined.*
