# Supplementary material for: Long-read sequencing reveals pre-meiotic gene conversion in sperm
Source: Nature. 2026 Aug 26;657(8133):981–7. doi: 10.1038/s41586-026-10901-0 (PMC13600919; doi:10.1038/s41586-026-10901-0)
Supplement: Supplementary file 3 [file 41586_2026_10901_MOESM4_ESM.pdf]

nature portfolio

Peer Review File

Long-read sequencing reveals pre-meiotic gene conversion in sperm

Corresponding Author: Dr Richard Durbin

This file contains all reviewer reports in order by version, followed by all author rebuttals in order by version.

Version 0:

Reviewer comments:

Referee #1

(Remarks to the Author)
Schweiger and Lee, et al. use accurate long-read sequencing of human sperm DNA to identify 4460 crossovers (COs) and 1759 non-crossovers (NCOs) across 13 donors. The main contribution of this work is in the demonstration of the method, which can be used for future studies of recombination from individuals rather than pedigrees or populations. On its face, the idea of sequencing sperm with long reads to build recombination maps is not novel, but this paper goes beyond previous methods in establishing a pipeline for identifying non-crossover gene conversion events, which are very difficult to detect and relatively understudied compared to crossovers. To do this, the authors established careful filters to distinguish sequencing errors from true NCO gene conversions. While the discoveries in this paper do not go far beyond what was already achieved by previous human pedigree-based maps (Williams et al. 2015, Halldorsson et al. 2016), this work does help to corroborate and refine previous estimates of NCO properties such as their tract length and magnitude of GC-biased gene conversion (gBGC). They also shed some new light on the rate and potential origins of complex NCO events in males.

Major points:
1) One of the major findings of the paper is that gBGC does not appear to depend on the number of converted SNPs in a tract, as previously described in mice and computed from reanalysis of a small number of published human pedigree-derived NCO events (Li et al. 2019). However, the methods section of this manuscript does not adequately describe how GC-bias was computed. It is worth exploring this phenomenon in detail. Do multi-SNP tracts tend to be much longer, given the low SNP density in humans? Does gBGC change when the analysis is restricted only to co-converted SNPs within 50 bp, or any SNP within 50 bp of another SNP (as in Li et al. Fig 4b)? Does the GC richness of the PRDM9 motif, and its erosion, affect the ascertainment of NCOs or the estimate of gBGC in this study? Can you break down conversions further by all possible base changes (as in Li et al. Fig 4c)?
2) Another major finding is that NCO tracts are very short (estimate of 34.6 bp mean), and the discussion section would benefit from further elaboration on how this estimate compares to other estimates in the literature. Why might this estimate differ from pedigree-based estimates? How does it compare to estimates in mice and other species?
3) Fig 3 and related section of text: It's unclear whether correction for multiple testing was performed when reporting the statistical significance of all of the results presented in this figure. It may also help to simplify this figure and this section of text. It reads as a list of observations, many of which lack clear interpretability. It's nice to see the expected changes in individuals with different PRDM9 alleles. However, it's hard to interpret many of the reported significant changes in other individuals, such as the meaning of the low fraction of NCO reads containing DSB-PRDM9 motifs for individual AA5. I would suggest simplifying this section to highlight the most biologically meaningful results.
4) It would be more useful to the community if the supplemental excel spreadsheet contained more information about each event. Did it overlap a DSB-PRDM9 motif? For NCOs, which SNPs converted? Which allele converted to which allele?

Minor points:
1) It would help in the genome-wide maps of event positions (Fig 2A) to see the positions of regions filtered out by the Tandem Repeat Finder filter that was applied (or any similar filters/gaps, e.g. where there are haplotype assembly gaps). This will help to visualize where centromeric and pericentromeric heterochromatin are.
2) It appears in the genome-wide maps of event positions (Fig 2A) that there is an enrichment of q-arm events near the likely positions of the centromeres of the acrocentric chromosomes. Is this a statistically significant pattern? How does it contrast with peri-centromeric rates on non-acrocentric chromosomes?
3) Please clarify exactly how "Distance to PRDM9 motif" is computed in the legends for Figure 2C-D? Is this the distance

from the first base of the oriented motif? How often do converted SNVs overlap the PRDM9 motif itself (i.e. do you see any instances of hotspot erosion in action)?
4) Line 161 “the chance of two independent recombination events between any pair of SNVs is extremely low, even in hotspots”: Can you estimate this?
5) Line 251: Is there anything in common for reads with non-complex events that do not overlap DSB-PRDM9 motifs? Do they contain PRDM9 motif matches but fail to overlap the DSB peak set? Are they generally GC-rich like the PRDM9 motif?
6) Lines 224, 253, others “P<1e-30”, “P<1e-100”: I would suggest reporting any P-value smaller than 1e-16 as “P<1e-16”, which is the typical limit of machine precision and commonly used to represent p-values that are essentially 0.
7) Line 294 “This is long compared to recent estimates”: provide a citation
8) Lines 309-312: Suggest shortening. This is too much detail about model comparison.
9) Line 323 and 326: State n, the number of tracts, when providing the confidence interval.
10) Line 326: Was this for single or multi-SNP tracts or both combined?
11) Line 424 “34 bp”: on line 307 you report 34.6, which should round to 35.
12) Lines 430-434: It’s important to emphasize how the present study corroborates or extends the findings of Halldorsson et al 2016, who showed that complex NCOs were “not overrepresented in male DSB regions”.

(Remarks on code availability)
This is a beautiful example of reproducible and well-documented code, including full snakemake pipelines

Referee #2

(Remarks to the Author)
The authors of this manuscript present an analysis of crossover (CO) and non-crossover (NCO) gene conversion events in human sperm samples. Using the long-read Pacific Biosciences platform, they generate haplotype-resolved assemblies for a number of sperm donors and infer likely recombination events by re-aligning reads to those assemblies. Their approach enables the identification of CO and NCO events in individual sperm cells, and at fine-grain resolution throughout the genome. The authors find that COs and NCOs are enriched near PRDM9 binding motifs, though these enrichments are biased down- and up-stream of binding motifs, respectively. Finally, the authors develop a simple statistical model and estimate that around 98% of NCOs are very short (~50bp), while 2% are much longer (on the order of kilobases).

Overall, I found this manuscript to be compelling and clearly-written. The authors' approach is easy to understand, and their conclusions follow logically from the results. While I am slightly underwhelmed by the novelty of the results, I believe that they new details to the growing body of knowledge about patterns of meiotic recombination in the human germline.

Comments and suggestions:

L166-170: The size distribution of NCO events is a critical feature of this manuscript. Since NCO events are often defined by the presence of a single haplotype "switch," I would be interested in seeing additional data to rule out the possibility that these switches are not errors. In particular, I am curious to know whether the base qualities of converted SNPs within NCO tracts are lower, on average, than the base qualities of flanking SNPs. For example, the authors might plot the average base qualities of converted SNPs as well as the average base qualities of flanking SNPs 5 base pairs up- and down-stream. The authors discuss their overall base quality filtering criteria in the Methods; if their current criteria already address this potential issue, they should mention it in the text.

L192: The authors speculate that the COs and NCOs observed in control (blood) data might reflect either somatic recombination events or a lower bound on the rate of erroneous CO/NCO calls. Although the number of CO/NCOs in control samples is small, did the authors analyze any other characteristics of those calls? For example, are the control CO/NCOs intervals larger, on average, than the CO/NCOs observed in sperm? I'm doubtful that this is the case, but one could imagine that large (>1kbp) NCO events are more likely to be errors; if all of the control NCOs are large, it might be suspicious.

L245: The authors examine the overlap between inferred CO/NCOs and previously-published DMC1 ChIP-seq peaks. Since the DMC1 peak data are derived from the testis of a PRDM9 A/A individual, did the authors exclude CO/NCO data from the AB, AD, and AN sperm donors in this analysis? Presumably, the AB, AD, and AN donors will have different patterns of DMC1 hotspots than the homozygous AA donors given their heterozygous PRDM9 alleles. If this is the case, it could be made clearer in the text; if not, I think that the authors should provide some justification in the text for the heterozygous donors' inclusion.

Table 1: Minor, but it would be helpful to include the fraction of calls attributed to each category (CO, NCO, ambiguous, etc.) in each row. That way, it would be easier to see e.g., the differences in CO vs. NCO abundance in the Sequel II and Revio callsets.

L262: The authors restrict their PRDM9 analysis to COs with inter-SNP distances < 500bp. Perhaps I'm misunderstanding, but shouldn't the X-axes in Fig. 2C and Fig. 2D be bounded by (-500, 500)? If the crossover interval is always less than 500bp, and the crossover interval contains the PRDM9 motif (L261-262), how can a position in the crossover interval be >500bp away from the center of the PRDM9 motif? It seems as though these figures might show the distance to the closest PRDM9 binding motif, even if that motif doesn't overlap the CO interval.

L268: Similarly, I may have missed it, but how was the permutation test performed? If details are not already provided in the text, they should be included.

L324: The authors state that when they limit to NCOs that overlap DSB-PRDM9 motifs, they observe a higher GC bias. Presumably, the Fisher's p-value is derived from a contingency table comparing the count of G/C and non-G/C conversions at NCOs that do or do not overlap DSB-PRDM9 motifs; it would be helpful to spell this out in the text or Methods. Also, is this comparison only including the single-SNP conversions?

L359-360; Fig. 3A: For each donor, the authors calculate the fraction of reads overlapping a PRDM9 motif that coincides with a DSB hotspot from the Hinch et al. study. Did the authors only calculate the fraction of reads overlapping the 31bp DSB-PRDM9 A/A motif described earlier? If so, is it surprising that the donors with only one copy of the PRDM9 A allele have fractions that are very similar to those of donors with two copies of the A allele?

L441-442: The authors speculate about some reasons for the up- and down-stream biases of CO and NCO enrichment near DSB-PRDM9 motifs. While these biases are statistically significant, could the authors elaborate on how surprising they are? For example, what is known about the "precision" of DSBs initiated by PRDM9/DMC1, and subsequent crossover resolution? Are DSBs always generated near the exact center of the PRDM9 binding motif? Is it possible that heterozygosity (i.e., SNP density) in the sperm donors could affect the up- and down-stream "bounds" of CO/NCO intervals, producing the biases seen in Fig. 2C-D? This is a non-essential suggestion, but in general, I think it would be helpful to speculate about alternative processes that may have generated the spatial CO/NCO biases.

Referee #3

(Remarks to the Author)
The manuscript focuses on meiotic recombination which is a topic of general interest because it is crucial for evolution, fertility and genome integrity in the germline (including the human germline). Meiotic recombination results in either reciprocal (called crossover) or non-reciprocal (called non-crossover) genetic information transfer. A long-standing ambition of the meiosis field has been to describe the genomic locations and properties of meiotic crossovers and non-crossovers with ever increasing detail. This is because properties of crossovers and non-crossovers provide indirect hints about the mechanisms of meiotic recombination.
Whereas past efforts have comprehensively mapped sites of recombination initiation and crossovers, non-crossover maps have been somewhat lagging in coverage due to the difficulty in detecting non-crossovers (detection relies on recognizing gene conversions at small polymorphisms). Nonetheless, general properties of crossovers and non-crossovers have been described, including GC-bias in recombination associated gene conversions and shorter gene-conversion tracts in non-crossovers than in crossovers.

The manuscript describes a long-read-sequencing method that is useful for mapping both crossover and non-crossover recombination outcomes in meiosis at a higher coverage as compared to earlier methods. Use of the method is shown in pools of human sperms. The manuscript is a descriptive study whose primary merit is the reconfirmation of prior results with an improved method. Specifically, it broadly reconfirms the conclusions of previous studies about the tract lengths and GC-bias of gene conversions in crossovers and non-crossovers. The presented data suggest a previously unreported difference in the estimated center positions of crossovers and non-crossovers relative to DSB hotspot sites, which are specified by PRDM9 binding. This is potentially interesting, but it only incrementally extends conclusions of prior studies that showed an influence of PRDM9 occupancy on recombination outcomes at hotspots. It is difficult to reliably assess the size of the difference as the position of crossovers is uncertain due to the spread of flanking markers. Furthermore, the biological significance of this observation is uncertain, diminishing its impact.
All in all, the manuscript reports on technology improvements without providing major new concepts or new mechanistic insights. The described methodological improvement will mainly interest researchers whose work relies on mapping recombination sites in gametes, and the results will have only limited impact in the broader meiosis/genetic fields or beyond. Therefore, in my opinion, the manuscript would only fit journals specialized in the relevant field/methods.

Additional comments
The manuscript is well written presenting a balanced discussion of the results in relation to the knowledge in the field. Thus, no major improvement is needed in this regard, though including a reference to manuscript DOI: 10.1093/genetics/iyaa013 could be considered.
The manuscript emphasises the detection of variation in the properties of crossovers and non-crossovers (Fig. 3 and lines 327-410) between individuals with the same genotype, and between repeat samples from the same individual at distinct ages or between monozygotic brothers. It is not clear what is the biological significance of these results, and to what extent these observations reflect stochasticity in sampling from potential crossover and non-crossover sites in each sample. It would be informative to split at least some of the biological samples and sequence them in replicates to test the effect of sampling stochasticity and the reproducibility of the method.
Although the statistical tests are described in the methods, the description of the statistical test is missing from Fig. 3. And related extended figures 5-8. The presentation of P values should be also considered in the figures or figure legends.

Version 1:

Reviewer comments:

Referee #1

(Remarks to the Author)
All of my concerns have been sufficiently addressed in the revised manuscript, which provides excellent tools and resources for the recombination community. I have no further comments or recommendations. - Nicolas Altemose

Referee #2

(Remarks to the Author)
The authors did a very thorough job addressing the core concerns of all reviewers, especially in ruling our sources of contamination and expanding the dataset to add confidence. The manuscript is indeed strengthened.

Referee #3

(Remarks to the Author)
The manuscript describes a long-read sequencing method for mapping both crossover (CO) and non-crossover (NCO) recombination outcomes in meiosis at higher coverage compared to earlier methods. In the first version of the manuscript (from last summer), the method was applied to pools of human sperm. The revised version presents deeper sequencing of the same sperm samples and includes additional data from somatic blood cells.
Despite the additional sequencing data, my main concerns remain unaddressed. The manuscript is a descriptive study whose primary contribution lies in reconfirming prior findings using an improved method. Specifically, it broadly reiterates established conclusions from previous studies regarding (i) the tract lengths and (ii) GC-bias of gene conversions in NCOs, as well as (iii) an upstream shift of NCO sites relative to PRDM9-marked DNA double-strand break (DSB) sites—consistent with a previously suggested role for PRDM9 in DSB repair. The manuscript also comments on inter-individual variation in recombination and on small nucleotide variants (SNVs) or mutations in somatic cells that may indicate somatic NCOs. However, these observations are unsurprising and/or insufficiently analyzed (see detailed comments below) and therefore do not yield significant new insights.
In summary, I maintain my original assessment that the manuscript presents technological improvements without delivering major conceptual or mechanistic advances. The described methodological refinements will mainly interest researchers focused on recombination site mapping in gametes. The broader impact on the fields of meiosis or genetics remains limited. Accordingly, I believe the manuscript would be more suitable for a specialized journal focused on the relevant field or method development.

Detailed Comments
Confidence in NCO Calling:
The manuscript identifies short/simple NCOs based on two nearby switches between haplotypes in PacBio long reads. These putative NCO events are enriched at PRDM9-associated DSB sites, indicating that many may represent bona fide NCOs. Notably, this enrichment is lower than that observed for COs at DSB sites. This difference may originate from potential sample contamination (noted in the methods) or sequencing or technical artefacts, as these errors generate false positive NCOs with considerably higher likelihood than false COs.
Notably, the authors discard approximately 75% of candidate recombinant reads using filters designed to detect contamination, assembly errors, or technical artefacts. It would be informative to report the frequency of different error types. In particular, the number of SNVs that do not match either haplotype would shed light on the potential contributions of sequencing errors or contamination to the inferred NCO count. Such errors could result in reads filtered out due to mismatches with both haplotypes, or in reads that erroneously appear as genuine NCOs leading to their erroneous inclusion into NCO counts in the current manuscript version.
Approximately 30% of the putative NCOs in the dataset are not associated with PRDM9 sites. It is important to determine whether these could be explained by contamination or sequencing artefacts. Additional analyses should test whether these PRDM9-independent NCOs (or those inconsistent with the genetic map) exhibit a GC-bias. A lack of such bias would further suggest that they are false positives. The manuscript also proposes that NCOs/mutations originating from mitotic germ cells may underlie NCOs that do not match the genetic map, and that these mitotic NCOs in germ cells resemble putative NCOs that accumulate with age in somatic cells. If so, an age-dependent increase in the frequency of such PRDM9-independent NCOs in sperm should be shown and analyzed explicitly.

Somatic NCOs:
Similar concerns apply to putative NCOs detected in somatic cells. There is a modest enrichment of these events in fragile sites (26% vs. 22% background), potentially suggesting an association with somatic DNA breaks. However, the analysis is incomplete, and it remains unclear whether the observed SNV alterations (termed NCOs in the manuscript, though “gene conversions” would be more accurate, as NCO refers to the process) are instead artefacts arising from sequencing errors, contamination, or DNA repair mechanisms unrelated to homologous recombination.
It is therefore essential to present data on the frequency and distribution of (i) small deletions, (ii) insertions, and (iii)

mismatches to the haplotypes—since none of these can arise from NCOs. Their prevalence would help assess whether the observed SNV alterations truly result from gene conversions during NCO-type recombination in somatic cells. Moreover, an analysis of GC-bias in the putative somatic gene conversions and a detailed analysis of their genome-wide distribution would be informative.
The manuscript rightly states that most somatic recombination takes place between sister chromatids, rather than homologous chromosomes. This widely accepted knowledge is invoked to provide an explanation for the abundant (putative) NCOs but rare COs in somatic cells. However, this interpretation is incorrect: recombination between sister chromatids neither generate observable NCOs (gene conversions) nor observable COs because sister sequences are identical preventing the detection of recombination outcomes by sequencing. Both observable COs and observable NCOs only result from homologous recombination between homologs, which is considered rare in somatic cells. Therefore, the data must convincingly exclude alternative repair processes that could yield repair products erroneously identified as NCOs. This is essential before drawing any conclusions regarding somatic NCO recombination.

Variation in Recombination Sites:
The manuscript emphasizes variation in CO and NCO properties between individuals with the same PRDM9 alleles, between repeated samples from the same individual at different ages, and between monozygotic siblings. In my initial review, I noted that the biological significance of these observations was unclear, and that some may simply reflect stochastic sampling of potential recombination sites. I recommended performing replicate sequencing on split biological samples to test the contribution of sampling noise and to evaluate the method's reproducibility.
The authors addressed this by including additional sequencing of some sperm samples and pooling old and new sequencing data from each sample. However, instead of pooling old and new datasets, the manuscript should present the new data side-by-side with the original data as technical replicates to allow the reader to assess variability arising from sampling or technical factors. Cross-referencing the newly generated pooled data (Fig. 3) with the original dataset suggests considerable variation between technical replicates. For instance, in the current data, NCOs in the AA2-t2 sample overlap PRDM9-DSB hotspots significantly more than in the AA2-t1 sample, whereas the previous data showed the opposite trend. A significant shift is also observed in the NCO characteristics in the AA4 sample following deeper sequencing. These discrepancies suggest that much of the reported inter-individual variation may stem from sampling effects or technical variation rather than true biological differences. This should be clearly acknowledged and discussed to explicitly warn readers that the observed variations have questionable biological meaning and significance.

Minor Comments:
• When referring to enrichment of COs or NCOs in various genomic regions (e.g., PRDM9 sites), it is unclear what is meant by "all reads." Please clarify whether this refers to all recombinant reads, all candidate recombinant reads, or all high-quality SNV-containing reads.
• There may be a typo in the sentence: "In contrast to both COs and NCOs, only 21% of complex event reads overlap a DSB-PRDM9 motif (not significantly different from the background 25%)." Based on an earlier statement—"We found that 67% of CO reads and 45% of NCO reads overlapped a DSB-PRDM9 motif (Fisher's exact test, P<1e-10), compared with 13.5% of all reads"—should "background 25%" instead read "background 13.5%"?

Version 2:

Reviewer comments:

Referee #2

(Remarks to the Author)
After reading the revised manuscript and the detailed rebuttal, my view is that the authors have addressed the core technical concerns raised earlier, including many of Reviewer #3's points regarding contamination, sequencing artefacts, and sampling noise. The expanded analyses, in particular the quantification of haplotype-inconsistent bases, the fragile-site enrichments, the GC-bias stratification, and the side-by-side comparison of replicate sequencing batches, strengthen the evidence that a large fraction of the identified non-crossover events represent genuine biology rather than technical noise.

The dataset now makes a strong case for a significant PRDM9-independent class of sperm NCOs with properties that differ from canonical meiotic recombination. This class shows:
(1) attenuated or absent GC-bias
(2) clear enrichment at fragile genomic regions
(3) lack of association with PRDM9-directed DSB maps
(4) close similarity to gene-conversion–like events observed in matched somatic tissue.
These signals appear consistently across individuals and across independently generated sequencing batches, and they sharpen under stricter filtering. For these reasons, I view the existence of a substantial non-meiotic component of sperm NCOs as well supported.

The authors interpret this PRDM9-independent class as arising from pre-meiotic, mitotic-like templated repair, and this is a plausible and well-reasoned explanation. However, I would emphasize, as the authors themselves now state more explicitly, that the assignment to a specific repair pathway or developmental stage is inferential. The data show that these events are not typical meiotic NCOs, but they cannot by themselves distinguish among several possible non-meiotic mechanisms. Therefore, while the evidence for a genuine non-meiotic class is strong, in my view the mechanistic conclusion should be

framed as consistent with, rather than definitively demonstrating, mitotic homologous recombination in germline precursors.

Referee #4

(Remarks to the Author)

In this manuscript the authors use Pacbio HiFi long read sequencing to identify non-crossover DSB repair products in sperm and characterize their features. The major claim is that a large fraction of NCOs appears to originate from DSBs arising during mitotic growth rather than from meiotic DSBs. The authors also find variation in recombination events between donors, including donors who share the same PRDM9 alleles, as well as between identical twins.

The presence of "mitotic" NCOs in sperm is not surprising in itself, but their frequency (30-40% of all observable NCOs) appears high. In non-meiotic cells, the majority of DSBs are repaired by non-homologous end joining, and the vast majority of those repaired by homologous recombination use the sister chromatid. Consequently, observable somatic recombination events are extremely rare. Unfortunately, the authors do not comment on this fact and do not attempt to estimate whether the high number of putative "mitotic" NCOs in sperm is even theoretically achievable through somatic inter-homologue recombinational repair. Without appropriate calculations and/or simulations, it is difficult to assess whether the "mitotic" NCOs identified in this study could indeed arise pre-meiotically. The fact that the authors observe NCOs with similar characteristics in blood samples could indicate that "mitotic" NCOs are indeed what they propose. However, it could just as well suggest that such NCOs represent a common artifact or a misinterpretation.

My other concern is the lack of GC bias in the conversion tracks of the “mitotic” NCOs. The authors use this as one of the criteria that supports their non-meiotic origin, stating that gBGC is specific to meiosis. As far as I know, GC bias is observed regardless of whether repair occurs in meiotic or somatic tissues. If the authors have evidence to the contrary, they need to provide appropriate citations.

Another criterion is that observed NCOs overlap PRDM9 binding motifs, known DSB sites or the sites of COs less frequently than observed COs do. Although this could indicate that a fraction of NCOs is of non-meiotic origin, this could also mean that these are misidentified NCOs, consistent with the fact that NCOs are much more difficult to detect.

The correlation with fragile sites is rather small (a 5% increase over a 21% background). It would be interesting to know whether “mitotic” NCOs share some other genomic features – e.g., do they show any correlation with promoters, enhancers, CGIs, etc.?

As for the other main findings, inter-individual variation in recombination hotspots among individuals with identical PRDM9 alleles has been previously reported. The authors show the variation in additional recombination features, but they are looking at only ~180 NCO events per individual, whereas there are ~40K hotspots in humans. These hotspots vary in strength and genomic context, and the observed inter-individual differences in NCO features may simply result from sampling different subsets of hotspots in different individuals.

The authors may ultimately be correct that a substantial fraction of NCOs are indeed produced pre-meiotically. However, the data presented and/or perhaps insufficient discussion leave some doubts regarding the validity of this conclusion. It would be very helpful if the authors could examine NCOs identified by a different approach to determine whether the “mitotic” NCOs can also be detected in that data.

Specific comments:

Abstract, line 40: "... we see non-crossover gene conversions with similar properties, increasing with age" - this is misleading, because, according to the main text, this correlation does not reach statistical significance.

Line 65: "... the presence of events weakly linked to PRDM9 hotspots" - it is not clear what the authors mean by that, and the reference is missing

Line 97: "... single sperm..." this could be interpreted as "single cell", suggest rephrasing

Line 101: "... previously unreported variation between individuals in patterns of recombination..." - this is an overstatement. E.g., hotspot variation in individuals carrying the same PRDM9 alleles has been previously reported.

Line 150. SNVs within tandem repeats have been removed. How does this affect reads within fragile sites?

Line 171: "... we detected 11 COs, 369 NCOs..." (in blood samples). The authors detected 7,143 COs and 2,382 NCOs in sperm samples. How do they explain that the fraction of NCOs (compared to COs) in blood is so much higher than in sperm? If I remember this correctly, around 8% of DSBs repaired through homologous recombination in somatic tissues result in COs rather than NCOs - a similar ratio (5-10%) as in meiosis. I did not quite understand the explanation provided in lines 173-175.

Lines 184-186. I found this sentence confusing, and I did not find the description in the methods section.

Fig. 2B and lines 197-198. I have some difficulty understanding what is what here; there are three color shades in the legend, but five on the figure. I realize that some color shades are created by overlap, but I can't figure out which overlaps produce which colors. It doesn't make it easier that the legend refers to grey color reads as "all reads", but the main text refers to them as "all remaining reads" – i.e., those that are left after CO and NCO reads are removed. I basically have to believe the authors' word that the distribution of NCO genetic length is bimodal.

Line 208 and citation 37. In the cited work, somatic gene conversion was investigated in cancer cells and could hardly be applied to regular tissues. It's got to be a better citation.

Line 211, citation 38. There is nothing about NCO enrichment at fragile sites in Blooms in the cited paper.

Lines 211-213: "Finally, they may lack the GC-biased allele transmission that is a hallmark of meiotic mismatch repair-mediated gene conversion". This is oddly put, because it sounds as if gBGC only occurs during meiosis. As far as I know, GC bias is present in both meiotic and somatic tissues, but because somatic DSBs are mostly randomly distributed, it is meiotic DSBs that create observable gBGC signatures (evolutionary changes in genomic sequence) at recombination hotspots.

Line 233: "previously published meiotic DSB map of 28,286 human recombination hotspots" – one of the listed references reports more than 34K DSB hotspots for an AA individual, what was the reason not to use all of them?

Line 270 and the paragraph below, GC-bias in NCO tracts. The fact that "somatic" NCOs in sperm and blood do not show GC bias may suggest that these are erroneous NCO calls. This will also explain why they are not associated with PRDM9 mo

Version 3:

Reviewer comments:

Referee #4

(Remarks to the Author)
I am happy with the thorough and thoughtful response provided by the authors, as well as the care taken in revising the manuscript. The analysis of additional datasets—particularly the pedigree studies—and the clever distinction between male and female data leave little doubt about the validity and importance of the findings.

I had some reservations about using germline-assigned reads from testicular biopsies as a proxy for pre-meiotic cells, given that the majority of testicular cells are at the pachytene spermatocyte stage or later, when meiotic COs and NCOs have already been established. I wonder whether the methylation data from Siebert-Kuss et al. could be further stratified to distinguish between pre- and post-meiotic stages. Regardless, the data as presented appear robust and show a trend in the expected direction, so this remains a minor issue.

Overall, this is an excellent and carefully executed study addressing a novel and important question, and I believe it will be of broad interest. I am happy to support its publication in Nature.

Open Access This Peer Review File is licensed under a Creative Commons Attribution 4.0 International License, which permits use, sharing, adaptation, distribution and reproduction in any medium or format, as long as you give appropriate credit to the original author(s) and the source, provide a link to the Creative Commons license, and indicate if changes were made.
In cases where reviewers are anonymous, credit should be given to 'Anonymous Referee' and the source.
The images or other third party material in this Peer Review File are included in the article's Creative Commons license, unless indicated otherwise in a credit line to the material. If material is not included in the article's Creative Commons license and your intended use is not permitted by statutory regulation or exceeds the permitted use, you will need to obtain permission directly from the copyright holder.
To view a copy of this license, visit https://creativecommons.org/licenses/by/4.0/

We appreciate the reviewers' thoughtful and constructive feedback, which has helped us strengthen our manuscript. In response to their suggestions, we have conducted a comprehensive reanalysis of our data and made several key improvements. In particular, we have doubled the amount of sperm sequencing data, significantly enhancing the robustness of our findings. Furthermore, we have analysed a substantial amount of comparable quality long read PacBio sequencing data from blood samples in the Platinum Pedigree dataset, providing an important complementary perspective on non-meiotic recombination. During this process, we also identified and corrected a subtle contamination signal. These refinements have not only improved the technical rigor of our study but have also led to a clearer and more biologically meaningful interpretation of our findings. We believe the revised manuscript now presents a stronger and more impactful story.

Referees' comments:

Referee #1 (Remarks to the Author):

Schweiger and Lee, et al. use accurate long-read sequencing of human sperm DNA to identify 4460 crossovers (COs) and 1759 non-crossovers (NCOs) across 13 donors. The main contribution of this work is in the demonstration of the method, which can be used for future studies of recombination from individuals rather than pedigrees or populations. On its face, the idea of sequencing sperm with long reads to build recombination maps is not novel, but this paper goes beyond previous methods in establishing a pipeline for identifying non-crossover gene conversion events, which are very difficult to detect and relatively understudied compared to crossovers. To do this, the authors established careful filters to distinguish sequencing errors from true NCO gene conversions. While the discoveries in this paper do not go far beyond what was already achieved by previous human pedigree-based maps (Williams et al. 2015, Halldorsson et al. 2016), this work does help to corroborate and refine previous estimates of NCO properties such as their tract length and magnitude of GC-biased gene conversion (gBGC). They also shed some new light on the rate and potential origins of complex NCO events in males.

Major points:
1) One of the major findings of the paper is that gBGC does not appear to depend on the number of converted SNPs in a tract, as previously described in mice and computed from reanalysis of a small number of published human pedigree-derived NCO events (Li et al. 2019).

However, the methods section of this manuscript does not adequately describe how GC-bias was computed.

We added a full description of the GC-bias computation to the methods section:

> To calculate gBGC of converted SNPs in a set of NCO reads, we consider only SNPs where one allele is G or C, and the other is A or T. The gBGC is then calculated as the fraction of converted SNPs with G or C out of this subset.

It is worth exploring this phenomenon in detail. Do multi-SNP tracts tend to be much longer, given the low SNP density in humans? Does gBGC change when the analysis is restricted only to co-converted SNPs within 50 bp, or any SNP within 50 bp of another SNP (as in Li et al. Fig 4b)?

We measured the flanking interval between the non-converted SNPs adjacent to the converted SNPs as an upper bound on the tract length. Under this definition, multi-SNP tracts are typically longer (~3,412bp on average) than single-SNP tracts (~2,265bp on average), as expected:

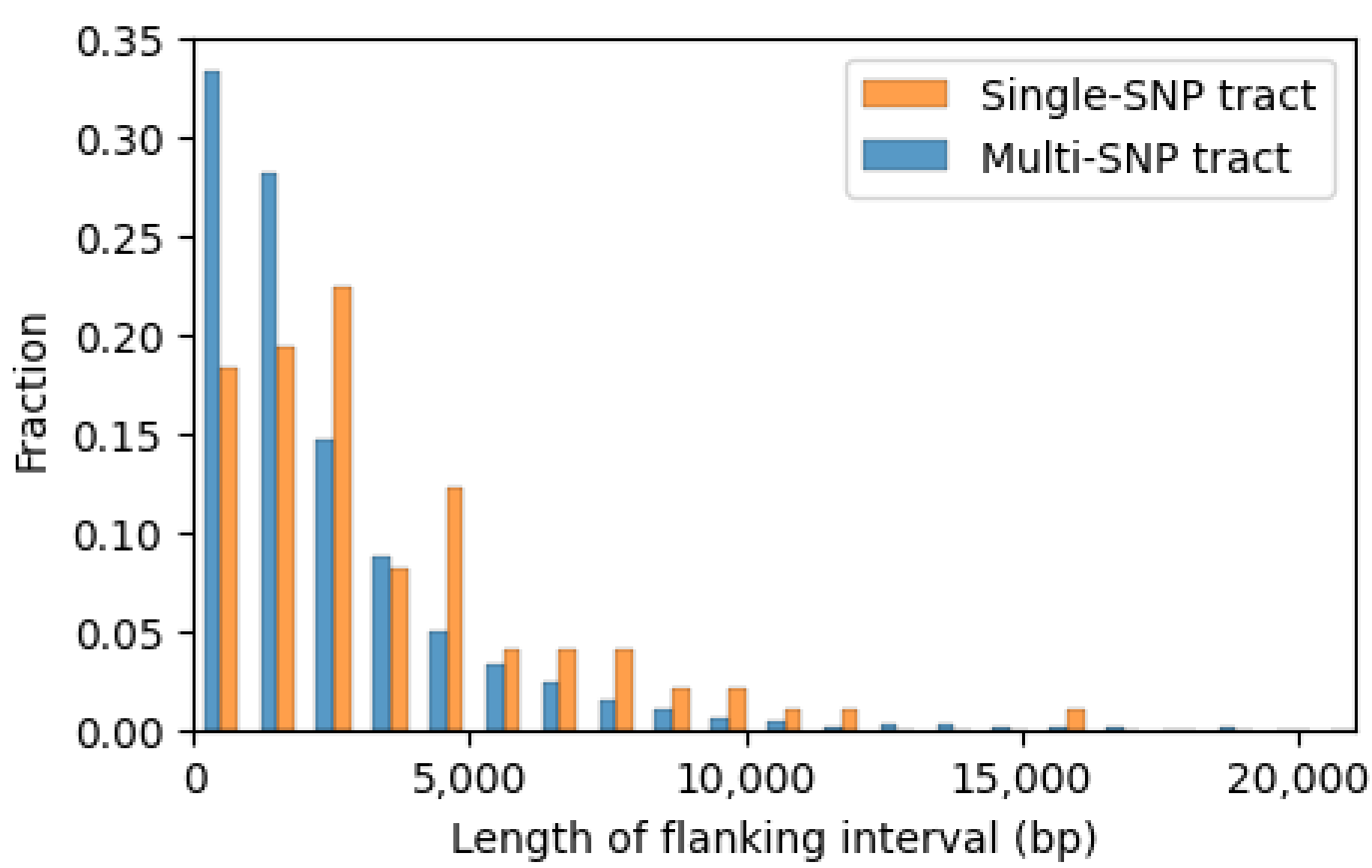

When restricting only to converted SNPs, which are within 50bp of another SNP, we observe a gBGC of 57.7% (CI 51.2%-64%), slightly lower but not statistically significantly different from the overall observed gBGC of 60.7%. Extending the analysis to various distances, as in Li et al. 2019, we again observe no statistically significant difference.

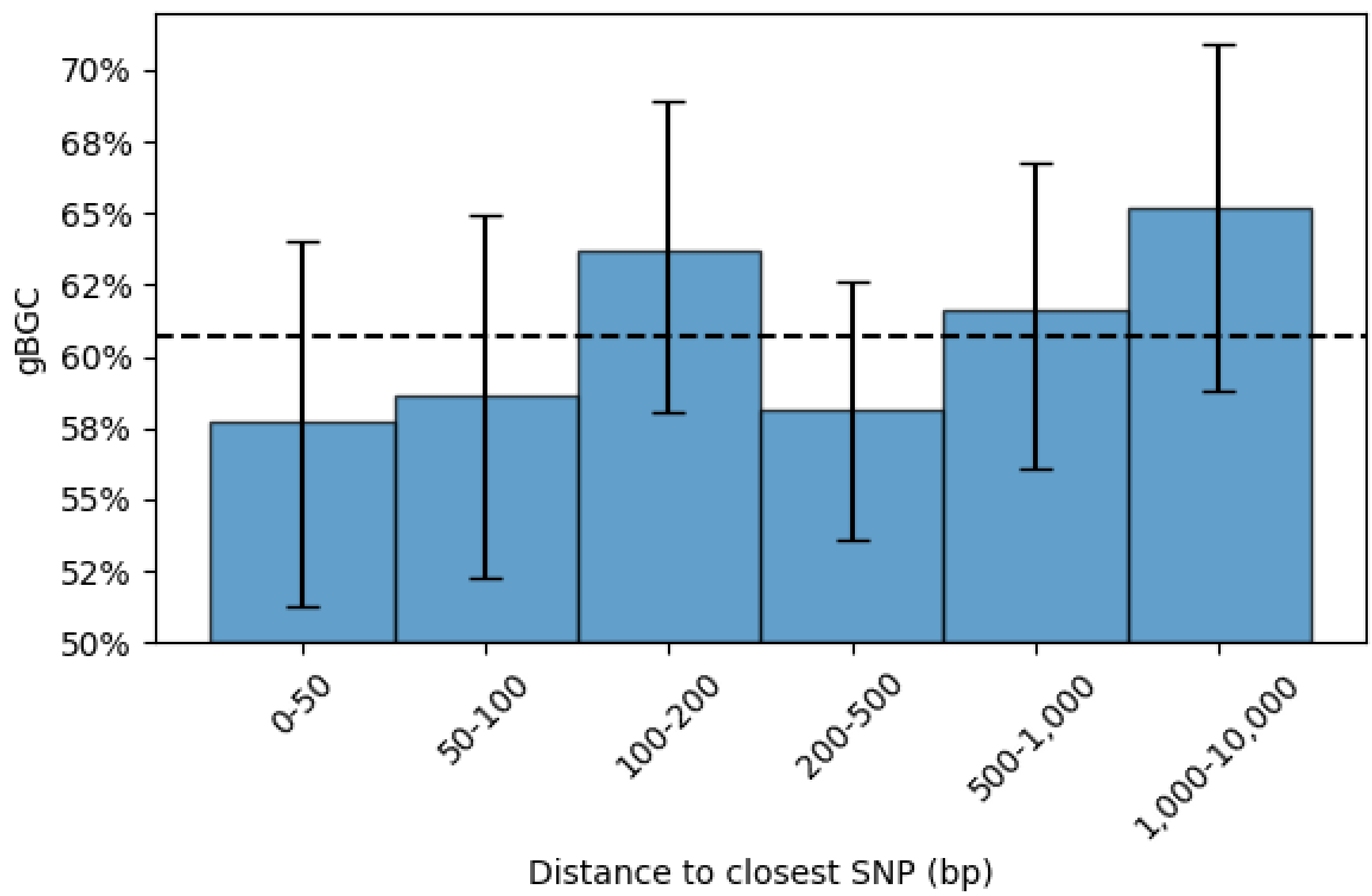

Further limiting to converted SNPs with co-converted SNPs within 50bp, we observed a gBGC of 67.7% (CI 56.9%-76.1%), higher than the 57.7% although again not statistically significant, perhaps due to the small sample size. We have updated the text to refer to Li et al. and added this last plot as a Extended Data Figure 7.

Does the GC richness of the PRDM9 motif, and its erosion, affect the ascertainment of NCOs or the estimate of gBGC in this study?

We see no evidence of such an effect. Only 18 of the GC-susceptible (A or T versus C or G) converted SNPs we detect fall within the 13bp core PRDM9 motif, and only 38 lie inside the full extended motif. Within the 38 gBGC was 63% (CI 46-77%), clearly not significantly different from the overall level. There is no general coverage variation as a function of GC content for PacBio reads, and we do not see any significant coverage difference at PRDM9 sites compared to elsewhere in the genome.

Can you break down conversions further by all possible base changes (as in Li et al. Fig 4c)?

We have done this, and now include it as Extended Data Figure 6:

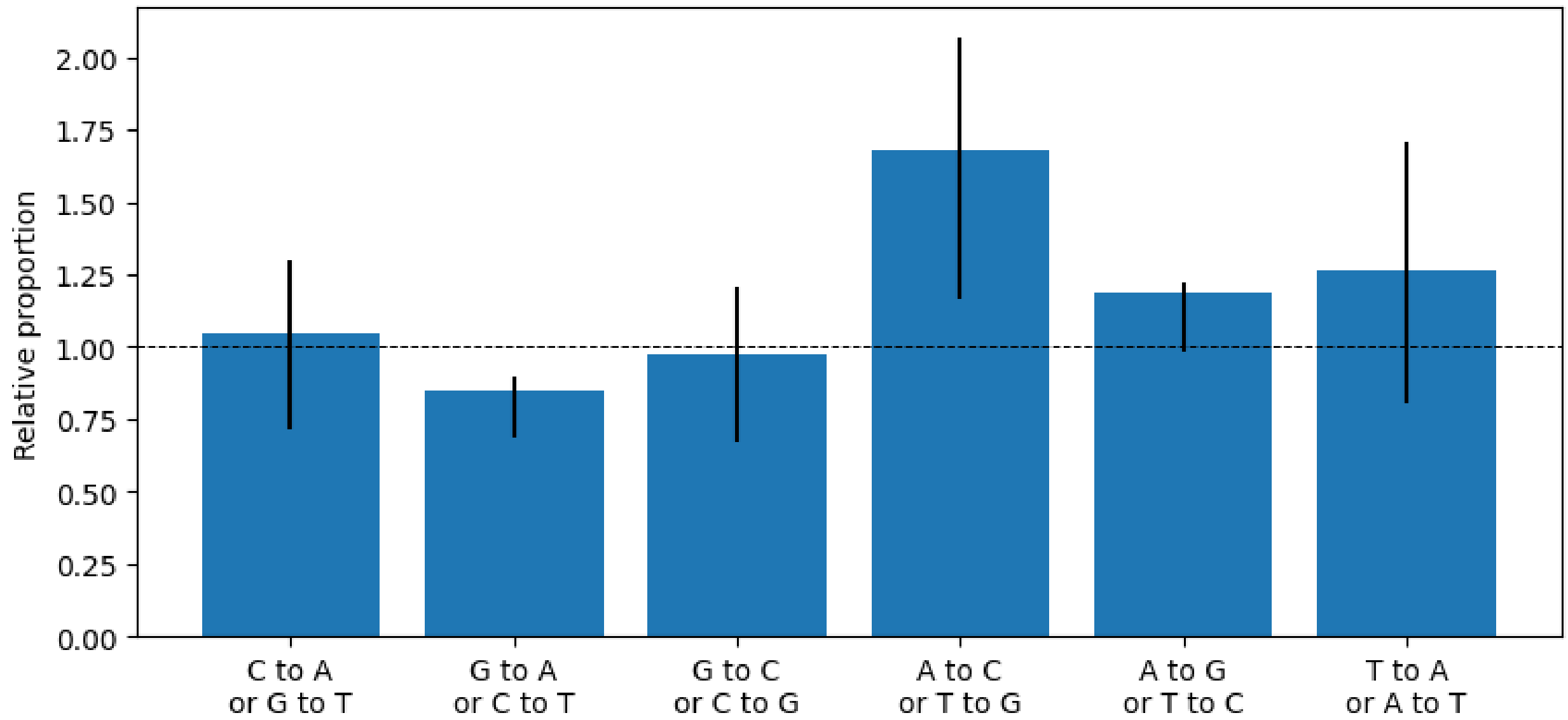

2) Another major finding is that NCO tracts are very short (estimate of 34.6 bp mean), and the discussion section would benefit from further elaboration on how this estimate compares to other estimates in the literature. Why might this estimate differ from pedigree-based estimates? How does it compare to estimates in mice and other species?

We added text to the Discussion section to facilitate comparison of NCO characteristics across different species and studies.

3) Fig 3 and related section of text: It's unclear whether correction for multiple testing was performed when reporting the statistical significance of all of the results presented in this figure.

It may also help to simplify this figure and this section of text. It reads as a list of observations, many of which lack clear interpretability.

It's nice to see the expected changes in individuals with different PRDM9 alleles. However, it's hard to interpret many of the reported significant changes in other individuals, such as the meaning of the low fraction of NCO reads containing DSB-PRDM9 motifs for individual AA5. I would suggest simplifying this section to highlight the most biologically meaningful results.

We have simplified the figure and the text to include only results that replicated robustly using the new sperm data, that are statistically significant including a strict Bonferroni

correction for multiple testing where that is appropriate. The comparisons between the monozygotic twin pair were not corrected for multiple testing because there was only one twin pair. We agree that the changes in individuals with different PRDM9 alleles are expected, but we also feel it is important to report and comment on significant differences between samples with the same PRDM9 alleles, and indeed between monozygotic twins who share the same genotype genome-wide (except for very few new mutations), suggesting that there may be non-genetic influences on the male recombination map.

4) It would be more useful to the community if the supplemental excel spreadsheet contained more information about each event. Did it overlap a DSB-PRDM9 motif? For NCOs, which SNPs converted? Which allele converted to which allele?

We have considerably extended the information contained in the spreadsheet, now including whether and where the DSB-PRDM9 motif overlapped the read; the associated peak; the motif strand; the number of converted SNPs and which alleles; lower and upper bounds on NCO tracts (where relevant), as well as the physical (bp) and genetic (cM) length of the reads.

Minor points:
1) It would help in the genome-wide maps of event positions (Fig 2A) to see the positions of regions filtered out by the Tandem Repeat Finder filter that was applied (or any similar filters/gaps, e.g. where there are haplotype assembly gaps). This will help to visualize where centromeric and pericentromeric heterochromatin are.

We modified Fig. 2A to include annotation for the fraction of filtered positions (in windows of 1Mbp), as well as the location of centromeres. We note that there are separate haplotype assemblies per individual; however we applied the *TRF* and *sdust* filters on the T2T reference genome, which is expected to closely follow the filters per assembly.

2) It appears in the genome-wide maps of event positions (Fig 2A) that there is an enrichment of q-arm events near the likely positions of the centromeres of the acrocentric chromosomes. Is this a statistically significant pattern? How does it contrast with peri-centromeric rates on non-acrocentric chromosomes?

It is already established that there is an increase in male recombination rate near the acrocentric centromeres on chromosomes 13, 14 and 15, but not 21 and 22 - our results reflect this. See the image below from Bhérer et al. (2017) Supp Fig 6.

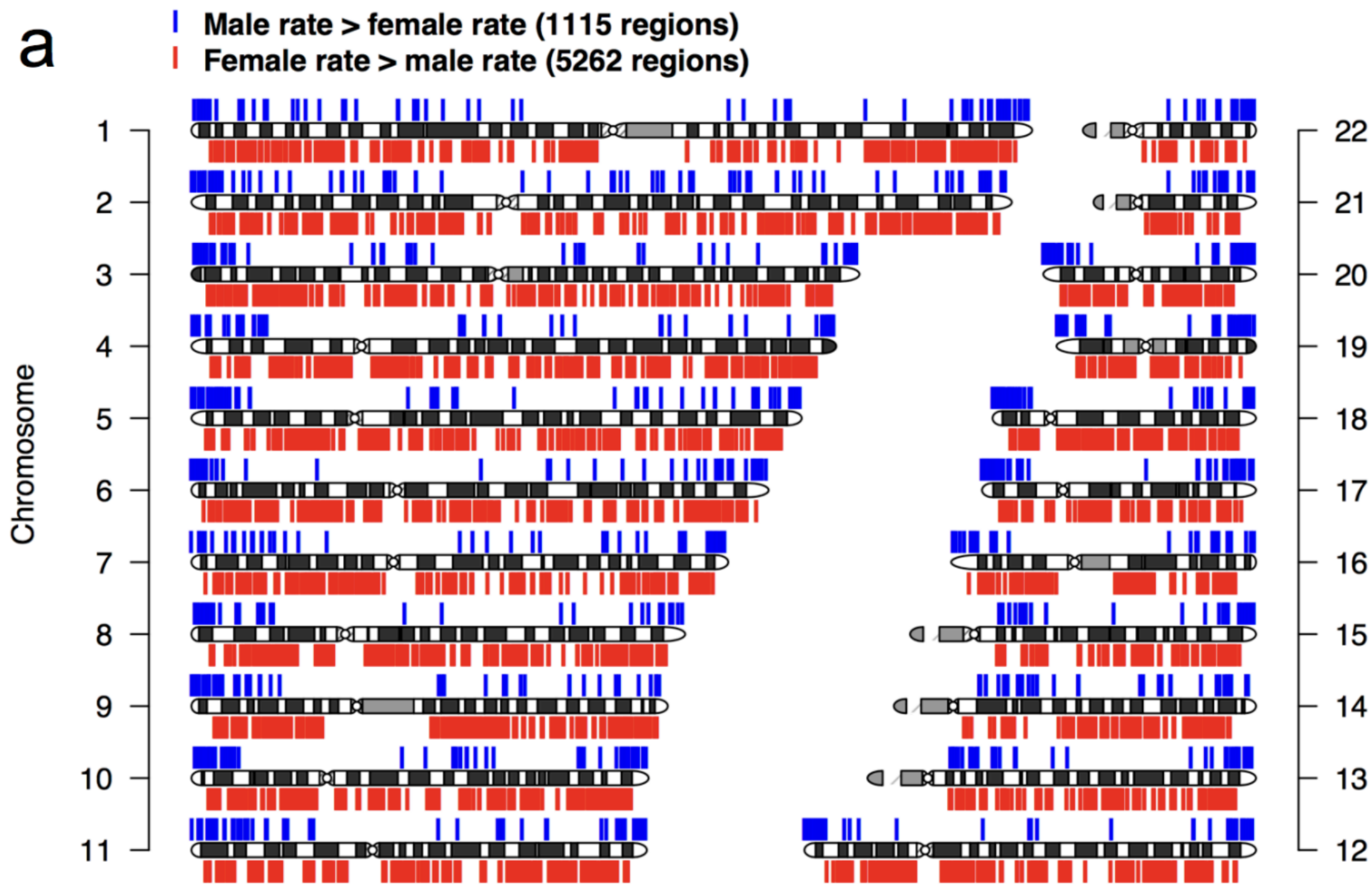

We counted the number of events within 0-10Mb of each centromere and within 10-20Mb, and tested whether the means for all acrocentric chromosomes are different than those for non-acrocentric chromosomes (Fisher's exact test). There was no significant difference, but this may be because we were combining 21 and 22 with 13-15. In any case we do not believe that we have a new observation to report here.

3) Please clarify exactly how "Distance to PRDM9 motif" is computed in the legends for Figure 2C-D? Is this the distance from the first base of the oriented motif? How often do converted SNVs overlap the PRDM9 motif itself (i.e. do you see any instances of hotspot erosion in action)?

The distance to the PRDM9 motif is computed to be relative to the center of the 31bp motif inferred and provided by the Hinch et al. data. We only see 42 converted SNPs that lie inside PRDM9 31bp motifs; and only 21 if we are limiting to the more informative, C-rich, 13bp motif. For each of these 42 motifs containing converted SNPs, we scored the motif with both the converted and the original alleles, using the PRDM9 position-specific scoring matrix. Of these, 22 decrease the motif score and 20 increase it, which is not statistically significant.

4) Line 161 "the chance of two independent recombination events between any pair of SNVs is extremely low, even in hotspots": Can you estimate this?

We have revised the text as below to estimate the probability that two independent meiotic recombination events would generate the same pattern of SNPs.

> Given the sequencing depth of our dataset (~20X – 160X), the probability of observing the same recombination molecule more than once by chance is low (Methods, 4.4×10^{-7} - 2×10^{-6} for CO and 1.6×10^{-6} - 7.8×10^{-4} for NCO), suggesting that such events are more likely the result of technical artefacts.

Methods

> We modelled recombinant events as a Poisson process to estimate the probability of observing the same gene conversion more than once. Given that approximately, 30 to 50 CO events and 200 to 1,000 NCO events occur per meiosis and that there are 25,000 to 50,000 meiotic recombination hotspots across the genome, the expected rate of CO and NCO events per hotspot per meiosis (λ) is between 0.0006 - 0.002 and 0.004-0.04, respectively. Assuming a Poisson distribution, the corresponding ranges of probabilities of observing two or more COs and NCOs within the same hotspot are given by $P(\text{Poisson } X \geq 2) = 1 - e^{-\lambda}(1+\lambda)$.

5) Line 251: Is there anything in common for reads with non-complex events that do not overlap DSB-PRDM9 motifs? Do they contain PRDM9 motif matches but fail to overlap the DSB peak set? Are they generally GC-rich like the PRDM9 motif?

We used FIMO from the MEME suite (using q-value < 0.01) to search for instances of the $PRDM9_A$ motif PWM provided by Hinch et al. in all recombinant reads, as well as in a random subset of 100,000 reads.

We find that with these settings 1317/2263 (58.2%) of CO reads that do not overlap a DSB-PRDM9 motif do include a PRDM9 motif, and similarly 565/1308 (43.2%) of NCO reads. This is significantly larger than the fraction of random reads with a PRDM9 motif that do not overlap a DSB-PRDM9 motif (25.32%).

Furthermore, we checked whether such reads are GC-rich. We found that recombinant reads exhibit a 42.2% GC content compared to 40.6% randomly sampled reads, a small but statistically significant increase, possibly due to undetected motifs or due to PRDM9 not binding to its motif.

6) Lines 224, 253, others “P<1e-30”, “P<1e-100”: I would suggest reporting any P-value

smaller than 1e-16 as “P<1e-16”, which is the typical limit of machine precision and commonly used to represent p-values that are essentially 0.

We have converted all sufficiently small P-values to P<1e-10.

7) Line 294 “This is long compared to recent estimates”: provide a citation

We removed this statement. Our revised estimated mean is a little shorter than before (though not inconsistent), but more relevantly, the recent literature is quite complex with respect to tract length, giving a mixture of broad ranges and multi-component estimates.

8) Lines 309-312: Suggest shortening. This is too much detail about model comparison.

We have removed these lines and condensed the text.

9) Line 323 and 326: State n, the number of tracts, when providing the confidence interval.

We added the number of tracts for both multi-SNV tracts and NCO reads overlapping a DSB-PRDM9 motif.

10) Line 326: Was this for single or multi-SNP tracts or both combined?

This is for both combined. We edited the text to clarify.

11) Line 424 “34 bp”: on line 307 you report 34.6, which should round to 35.

Done (with the new numbers).

12) Lines 430-434: It’s important to emphasize how the present study corroborates or extends the findings of Halldorsson et al 2016, who showed that complex NCOs were “not overrepresented in male DSB regions”.

Halldorsson et al. 2016 write “[Table 1 shows that] short NCO events were highly over-represented in male DSB regions and crossover recombination hotspots, while long NCO events were not over-represented in male DSB regions but were over-represented in crossover recombination hotspots.” They “hypothesize that all long NCO events might be complex”.

Like Halldorsson et al., we observe that short NCO events are over-represented in male DSB regions and reads with high genetic map length, i.e. hotspots, though to a lesser extent than CO events. We see no statistically significant difference between short and longer NCO events (those converting multiple SNPs separated by at least 100bp) in terms of read genetic map length, supporting their observation that long NCO events are also over-represented in crossover recombination hotspots. When we looked specifically at whether longer NCO events differ in their overlap with the DSB map this also was not significant in our data, contrasting with their results. Halldorsson et al. use 1000bp as a threshold rather than 100bp. When we do that, we only have 8 remaining events, and the tests remain non-significant. We note that Haldorsson et al. only have 12 “long NCO” events longer with converted SNPs over 1000bp apart in their male data set. Finally we discuss that at least some of the long NCO events may be complex.

Referee #1 (Remarks on code availability):

This is a beautiful example of reproducible and well-documented code, including full snakemake pipelines

Thank you.

Referee #2 (Remarks to the Author):

The authors of this manuscript present an analysis of crossover (CO) and non-crossover (NCO) gene conversion events in human sperm samples. Using the long-read Pacific Biosciences platform, they generate haplotype-resolved assemblies for a number of sperm donors and infer likely recombination events by re-aligning reads to those assemblies. Their approach enables the identification of CO and NCO events in individual sperm cells, and at fine-grain resolution throughout the genome. The authors find that COs and NCOs are enriched near PRDM9 binding motifs, though these enrichments are biased down- and up-stream of binding motifs, respectively. Finally, the authors develop a simple statistical model and estimate that around 98% of NCOs are very short (~50bp), while 2% are much longer (on the order of kilobases).

Overall, I found this manuscript to be compelling and clearly-written. The authors' approach is easy to understand, and their conclusions follow logically from the results. While I am slightly underwhelmed by the novelty of the results, I believe that they provide new details to the growing body of knowledge about patterns of meiotic recombination in the human germline.

We thank the reviewer for their thoughtful and positive comments. We have revised the manuscript to include additional data and clarifications in the text, and we hope that these changes help make the novelty of our findings clearer - particularly the evidence for a non-meiotic as well as a meiotic source of NCOs.

Comments and suggestions:

L166-170: The size distribution of NCO events is a critical feature of this manuscript. Since NCO events are often defined by the presence of a single haplotype "switch," I would be interested in seeing additional data to rule out the possibility that these switches are not errors. In particular, I am curious to know whether the base qualities of converted SNPs within NCO tracts are lower, on average, than the base qualities of flanking SNPs. For example, the authors might plot the average base qualities of converted SNPs as well as the average base qualities of flanking SNPs 5 base pairs up- and down-stream. The authors discuss their overall base quality filtering criteria in the Methods; if their current criteria already address this potential issue, they should mention it in the text.

Following the reviewer’s suggestion, we compared the base qualities (BQ) of converted SNPs to those of the immediately flanking SNPs (one SNP upstream and downstream - there are almost no SNPs within 5bp and in any case our QC criteria would remove these).

The average BQ of converted SNPs was in fact slightly higher than that of the flanking SNPs: 86.2 vs. 81.6 for Sequel II; 40 vs. 37.2 for Revio, we believe because there is a lower threshold on the BQ of the converted SNP as part of our QC process. Consistent with this, the fraction of NCO reads where the converted SNP obtained a BQ higher or equal to that of the upstream flanking SNP was 72.4% for Sequel II and 100% for Revio (for Revio only base calls in the highest BQ bin were able to pass QC).

We note that the Methods section ("SNP calling and filtering") describes base quality threshold calibration, and additional details are provided in the Supplementary Note ("Calibration of base quality scores").

L192: The authors speculate that the COs and NCOs observed in control (blood) data might reflect either somatic recombination events or a lower bound on the rate of erroneous CO/NCO calls. Although the number of CO/NCOs in control samples is small, did the authors analyze any other characteristics of those calls? For example, are the control CO/NCOs intervals larger, on average, than the CO/NCOs observed in sperm? I'm doubtful that this is the case, but one could imagine that large (>1kbp) NCO events are more likely to be errors; if all of the control NCOs are large, it might be suspicious.

We thank the reviewer for raising this point. To further investigate potential artifacts in control (blood) samples, we analyzed PacBio CCS data from the 12 samples from the Platinum Pedigree consortium for which the data were obtained from primary blood cells, using the same pipeline as for sperm data. We detected 11 COs and 369 NCOs, corresponding to 0.4% and 40% of the rates seen in sperm, after correcting for coverage. This supports the expectation that COs are rare in somatic cells, likely due to preferential DSB repair using sister chromatids rather than homologs.

We also examined the characteristics of the NCOs detected in blood. These events are not longer than those observed in sperm. In fact, they lack the long-tract component seen in sperm-derived NCOs. Moreover, they are enriched at fragile sites, similar to the subset of sperm NCOs that do not align with the expected DSB hotspot map. These observations support the suggestion that a substantial subset of NCOs seen in sperm reflect mitotic-like repair mechanisms during development as in blood rather than technical artifacts.

We added a section in the main text describing the analysis and properties of these blood-derived recombinant reads.

L245: The authors examine the overlap between inferred CO/NCOs and previously-

published DMC1 ChIP-seq peaks. Since the DMC1 peak data are derived from the testis of a PRDM9 A/A individual, did the authors exclude CO/NCO data from the AB, AD, and AN sperm donors in this analysis? Presumably, the AB, AD, and AN donors will have different patterns of DMC1 hotspots than the homozygous AA donors given their heterozygous PRDM9 alleles. If this is the case, it could be made clearer in the text; if not, I think that the authors should provide some justification in the text for the heterozygous donors' inclusion.

We analyse the data with respect to individuals indicating their PRDM9 genotype in Figure 3 and discuss the results in the text.

Table 1: Minor, but it would be helpful to include the fraction of calls attributed to each category (CO, NCO, ambiguous, etc.) in each row. That way, it would be easier to see e.g., the differences in CO vs. NCO abundance in the Sequel II and Revio callsets.

We have added the fraction of calls in each category. We note that after the addition of new sequencing data for the TwinsUK samples using a Revio machine, the differences are no longer clearly seen.

L262: The authors restrict their PRDM9 analysis to COs with inter-SNP distances < 500bp. Perhaps I'm misunderstanding, but shouldn't the X-axes in Fig. 2C and Fig. 2D be bounded by (-500, 500)? If the crossover interval is always less than 500bp, and the crossover interval contains the PRDM9 motif (L261-262), how can a position in the crossover interval be >500bp away from the center of the PRDM9 motif? It seems as though these figures might show the distance to the closest PRDM9 binding motif, even if that motif doesn't overlap the CO interval.

We have removed this analysis in the updated manuscript.

L268: Similarly, I may have missed it, but how was the permutation test performed? If details are not already provided in the text, they should be included.

We have added details about the permutation test in a new Methods section “Positional bias of converted markers relative to DSB-PRDM9 motif”.

L324: The authors state that when they limit to NCOs that overlap DSB-PRDM9 motifs, they observe a higher GC bias. Presumably, the Fisher's p-value is derived from a contingency table comparing the count of G/C and non-G/C conversions at NCOs that do or do not overlap DSB-PRDM9 motifs; it would be helpful to spell this out in the text or

Methods. Also, is this comparison only including the single-SNP conversions?

We clarified in the Methods that Fisher's exact test compares the number of A/T to G/C versus G/C to A/T conversions in NCOs that do or do not overlap a PRDM9 motif within the DSB map. This comparison includes both single- and multi-SNP NCO tracts. We also now explicitly state this in the main text.

L359-360; Fig. 3A: For each donor, the authors calculate the fraction of reads overlapping a PRDM9 motif that coincides with a DSB hotspot from the Hinch et al. study. Did the authors only calculate the fraction of reads overlapping the 31bp DSB-PRDM9 A/A motif described earlier? If so, is it surprising that the donors with only one copy of the PRDM9 A allele have fractions that are very similar to those of donors with two copies of the A allele?

As the reviewer notes, individuals with the A/B PRDM9 genotype have a similar fraction of motif-overlapping reads to those with the A/A genotype. This is consistent with findings from Pratto et al., who showed that the hotspot distribution in A/B individuals closely resembles that of A/A. The N allele is a novel variant that includes a duplication of the second zinc finger of A, so it is plausible that its binding preferences substantially overlap those of A, consistent with the similar fraction observed in the A/N individual. We are not aware of hotspot data for the AD allele, but it is known to be functionally distinct, and accordingly, we observe a different distribution. We now mention this briefly in the text.

L441-442: The authors speculate about some reasons for the up- and down-stream biases of CO and NCO enrichment near DSB-PRDM9 motifs. While these biases are statistically significant, could the authors elaborate on how surprising they are? For example, what is known about the "precision" of DSBs initiated by PRDM9/DMC1, and subsequent crossover resolution? Are DSBs always generated near the exact center of the PRDM9 binding motif? Is it possible that heterozygosity (i.e., SNP density) in the sperm donors could affect the up- and down-stream "bounds" of CO/NCO intervals, producing the biases seen in Fig. 2C-D? This is a non-essential suggestion, but in general, I think it would be helpful to speculate about alternative processes that may have generated the spatial CO/NCO biases.

We appreciate the reviewer’s suggestion for further discussion of the observed positional bias. We note that in the revised manuscript we have removed the CO result, which was only weakly significant before additional sequencing data and dropped below the significance threshold after adding it. The NCO result was replicated, strengthening the P-value. We therefore only discuss the NCO positional bias. Following further literature investigation, we have added to the paragraph in the discussion about this observation.

Referee #3 (Remarks to the Author):

The manuscript focuses on meiotic recombination which is a topic of general interest because it is crucial for evolution, fertility and genome integrity in the germline (including the human germline). Meiotic recombination results in either reciprocal (called crossover) or non-reciprocal (called non-crossover) genetic information transfer. A long-standing ambition of the meiosis field has been to describe the genomic locations and properties of meiotic crossovers and non-crossovers with ever increasing detail. This is because properties of crossovers and non-crossovers provide indirect hints about the mechanisms of meiotic recombination.
Whereas past efforts have comprehensively mapped sites of recombination initiation and crossovers, non-crossover maps have been somewhat lagging in coverage due to the difficulty in detecting non-crossovers (detection relies on recognizing gene conversions at small polymorphisms). Nonetheless, general properties of crossovers and non-crossovers have been described, including GC-bias in recombination associated with gene conversions and shorter gene-conversion tracts in non-crossovers than in crossovers.

The manuscript describes a long-read-sequencing method that is useful for mapping both crossover and non-crossover recombination outcomes in meiosis at a higher coverage as compared to earlier methods. Use of the method is shown in pools of human sperms. The manuscript is a descriptive study whose primary merit is the reconfirmation of prior results with an improved method. Specifically, it broadly reconfirms the conclusions of previous studies about the tract lengths and GC-bias of gene conversions in crossovers and non-crossovers. The presented data suggest a previously unreported difference in the estimated center positions of crossovers and non-crossovers relative to DSB hotspot sites, which are specified by PRDM9 binding. This is potentially interesting, but it only incrementally extends conclusions of prior studies that showed an influence of PRDM9 occupancy on recombination outcomes at hotspots. It is difficult to reliably assess the size of the difference as the position of crossovers is uncertain due to the spread of flanking markers. Furthermore, the biological significance of this observation is uncertain, diminishing its impact.

All in all, the manuscript reports on technology improvements without providing major new concepts or new mechanistic insights. The described methodological improvement will mainly interest researchers whose work relies on mapping recombination sites in gametes, and the results will have only limited impact in the broader meiosis/genetic fields or beyond. Therefore, in my opinion, the manuscript would only fit journals specialized in the

relevant field/methods.

Additional comments
The manuscript is well written presenting a balanced discussion of the results in relation to the knowledge in the field. Thus, no major improvement is needed in this regard, though including a reference to manuscript to (**Chromosome-wide characterization of meiotic noncrossovers (gene conversions) in mouse hybrids)** could be considered.

We have added this reference to the manuscript.

The manuscript emphasises the detection of variation in the properties of crossovers and non-crossovers (Fig. 3 and lines 327-410) between individuals ith the same genotype, and between repeat samples from the same individual at distinct ages or between monozygotic brothers. It is not clear what is the biological significance of these results, and to what extent these observations reflect stochasticity in sampling from potential crossover and non-crossover sites in each sample. It would be informative to split at least some of the biological samples and sequence them in replicates to test the effect of sampling stochasticity and the reproducibility of the method.

We have added replication data from the same samples as requested, substantially strengthening the statistical significance of the results.

Although the statistical tests are described in the methods, the description of the statistical test is missing from Fig. 3. And related extended figures 5-8. The presentation of P values should be also considered in the figures or figure legends.

We have added the required details in the figure legends.

Schweiger et al. response to second set of reviews

Below in our response the original text is in black, and our responses are in blue.

Referees 1 and 2 were satisfied with our edits and had no further comments.

Referee #1 (Remarks to the Author):

All of my concerns have been sufficiently addressed in the revised manuscript, which provides excellent tools and resources for the recombination community. I have no further comments or recommendations. - Nicolas Altemose

Referee #2 (Remarks to the Author):

The authors did a very thorough job addressing the core concerns of all reviewers, especially in ruling our sources of contamination and expanding the dataset to add confidence. The manuscript is indeed strengthened.

So all our comments are in response to Referee 3's remarks. In response to those and to a request from the editor to emphasize novel findings we have changed the title of the paper and restructured it to emphasize the key novel aspects, as outlined below following the first section of Referee 3's remarks.

Referee #3 (Remarks to the Author):

The manuscript describes a long-read sequencing method for mapping both crossover (CO) and non-crossover (NCO) recombination outcomes in meiosis at higher coverage compared to earlier methods. In the first version of the manuscript (from last summer), the method was applied to pools of human sperm. The revised version presents deeper sequencing of the same sperm samples and includes additional data from somatic blood cells.
Despite the additional sequencing data, my main concerns remain unaddressed. The manuscript is a descriptive study whose primary contribution lies in reconfirming prior findings using an improved method. Specifically, it broadly reiterates established conclusions from previous studies regarding (i) the tract lengths and (ii) GC-bias of gene conversions in NCOs, as well as (iii) an upstream shift of NCO sites relative to PRDM9-marked DNA double-strand break (DSB) sites—consistent with a previously suggested role for PRDM9 in DSB repair. The manuscript also comments on inter-individual variation in recombination and on small nucleotide variants (SNVs) or mutations in somatic cells that may indicate somatic NCOs. However, these observations are unsurprising and/or insufficiently analyzed (see detailed comments below) and therefore do not yield significant new insights.

In summary, I maintain my original assessment that the manuscript presents technological improvements without delivering major conceptual or mechanistic advances. The described methodological refinements will mainly interest researchers focused on recombination site mapping in gametes. The broader impact on the fields of meiosis or genetics remains limited. Accordingly, I believe the manuscript would be more suitable for a specialized journal focused on the relevant field or method development.

We appreciate the Reviewer's careful reading of our manuscript and acknowledge their expertise in recombination biology. However, we respectfully disagree with their conclusion that the work is primarily a methodological refinement without conceptual advance. Indeed, this summary does not reflect their detailed comments, which indicate that they recognise that we are proposing novel findings. They express systematic scepticism about these, suggesting that they are caused by errors without providing any evidence of that. They request additional information not requested in their original review, and additional tests. The information and test results support our conclusions, not the scepticism expressed by the reviewer. Below we explicitly detail the novelty

Most importantly, our study identifies a recombination process in human sperm that has not been documented in any prior genome-wide recombination map: a class of gene conversions arising **not associated with PRDM9 sites but rather with fragile sites,** which we also see in somatic tissue, suggesting that it derives from a **mitotic-like mechanism acting in the germline prior to meiosis.**

The prevailing view has been that all detectable recombination events in sperm are products of meiotic DSB repair, directed by PRDM9 (in humans). Our data challenge this assumption. We identify a substantial subset (approximately a third) of sperm NCOs with positional and sequence characteristics that: 1) lack PRDM9 association, 2) lack GC-bias, 3) show enrichment at fragile sites, similarity to somatic NCOs but incompatible with canonical meiotic programmed pathways. **These results suggest that germline genomes accrue gene conversion events across multiple mitotic cell divisions before meiosis, thereby contributing heritable changes independently of meiotic recombination**.

This observation is novel for several reasons:

1. Previous sperm-sequencing efforts lacked the combination of read length, accuracy, genome assembly quality, and somatic data needed to confidently detect and characterise these events genome-wide.
2. Although mitotic recombination has been studied in somatic cells, it has never been demonstrated as a distinct source of sequence variation in human gametes.

Contrary to the assertion that these results do not yield significant new insights or advances, we believe this work has multiple important implications:

- Medical genetics: It raises the possibility that such events influence fertility, affect gamete quality, and contribute to the heritable mutation load passed to offspring, which would have direct relevance to genetic counselling and reproductive health.
- Medical biology: It enables direct investigation of DNA repair pathways active in sperm precursor cells by comparing their molecular signatures with somatic gene conversions.
- Mutation biology: This insight could help explain specific properties of de-novo mutations such as clustering or sequence-context biases, that may originate from this newly identified source, thereby shedding light on the origins of heritable genetic variation and its contribution to human disease.
- Evolutionary genetics: It identifies a novel source of non-GC-biased gene conversion and haplotype reshaping that operates outside meiosis, altering models of sequence evolution and patterns of genomic diversity.

Beyond this central discovery, our study also reports another further advance of broad relevance. We report **reproducible inter-individual variation in NCO landscapes among individuals with identical PRDM9 genotypes**, including monozygotic twins, **persisting across repeated sampling**. This indicates that recombination outcomes are shaped by factors beyond DNA sequence, potentially pointing to developmental, environmental, or stochastic influences on recombination outcomes that have not been reported in prior genome-wide maps. This finding opens new directions for understanding inter-individual variation in genome stability and inheritance, including the potential to identify genetic or environmental modifiers of recombination fidelity, to study how early-life exposures or age influence germline genome maintenance, and to assess whether such variation contributes to differences in fertility, heritable disease risk, or evolutionary dynamics across populations.

In addition, we also reinforce recent observations about the distribution of meiotic gene conversion events made by Palsson et al. (Nature 2025) that that there is a **pronounced upstream positional bias of NCOs relative to PRDM9 motifs in humans, together with a bimodal distribution of GC-biased tract lengths**. These patterns provide a foundation for further mechanistic insight into double-strand break processing and repair pathway choice.

Together, these findings expand the conceptual framework for interpreting human recombination maps, opening lines of inquiry across genome biology, evolution, and medicine. We recognise that this reviewer does address these two points, and expresses scepticism about them. But they don't refute them. They ask for additional information and tests not asked for in the first revision and when we provide the information or carry out the tests they support our conclusions.

Detailed Comments

Confidence in NCO Calling:

The manuscript identifies short/simple NCOs based on two nearby switches between haplotypes in PacBio long reads. These putative NCO events are enriched at PRDM9-associated DSB sites, indicating that many may represent bona fide NCOs. Notably, this

enrichment is lower than that observed for COs at DSB sites. This difference may originate from potential sample contamination (noted in the methods) or sequencing or technical artefacts, as these errors generate false positive NCOs with considerably higher likelihood than false COs. Notably, the authors discard approximately 75% of candidate recombinant reads using filters designed to detect contamination, assembly errors, or technical artefacts. It would be informative to report the frequency of different error types. In particular, the number of SNVs that do not match either haplotype would shed light on the potential contributions of sequencing errors or contamination to the inferred NCO count. Such errors could result in reads filtered out due to mismatches with both haplotypes, or in reads that erroneously appear as genuine NCOs leading to their erroneous inclusion into NCO counts in the current manuscript version.

We note that in our Supplementary Note “Calibration of base quality scores” we had already done exactly what is requested here. There, we quantify the proportion of 1bp SNPs that do not match either haplotype and use this as a direct proxy for the false positive rate of NCO calls. The observed ratio is low, indicating that the vast majority of inferred NCOs reflect true gene conversion events rather than sequencing artefacts or contamination. Therefore, the enrichment of NCOs at PRDM9-associated DSB sites is unlikely to be substantially attenuated by false positives, and the lower enrichment relative to COs is best explained by **genuine biological differences rather than technical error**.

Moreover, the subset of NCOs with the weakest association to PRDM9 binding sites - the very events most susceptible to the reviewer’s suggested “regression to background” explanation - show a stronger and statistically significant enrichment at fragile sites than PRDM9-associated NCOs. This pattern is the opposite of what would be expected if these events were primarily false positives: random artefacts or contamination would dilute, not create, such a specific genomic enrichment. The fragile-site signal therefore reinforces the conclusion that this subset represents a genuine, biologically distinct class of NCOs.

Approximately 30% of the putative NCOs in the dataset are not associated with PRDM9 sites. It is important to determine whether these could be explained by contamination or sequencing artefacts. Additional analyses should test whether these PRDM9-independent NCOs (or those inconsistent with the genetic map) exhibit a GC-bias. A lack of such bias would further suggest that they are false positives.

We respectfully disagree that a lack of GC-bias would imply that PRDM9-independent NCOs are false positives. GC-bias is a hallmark of canonical meiotic gene conversion but is not necessarily expected for alternative pathways, such as the mitotic-like mechanism we propose. Indeed, the PRDM9-independent NCOs in our dataset form a coherent class: they lack GC-bias, are reproducibly enriched at fragile sites, and share positional and sequence characteristics with somatic NCOs detected in blood samples. This pattern is seen across multiple individuals and replicates, which is inconsistent with contamination or random sequencing artefacts that would dilute rather than create such specific enrichments. We therefore interpret the absence of GC-bias in these events as further evidence that they arise from a biologically distinct repair pathway as seen in somatic tissue, rather than as an indicator of technical noise.

The manuscript also proposes that NCOs/mutations originating from mitotic germ cells may underlie NCOs that do not match the genetic map, and that these mitotic NCOs in germ cells resemble putative NCOs that accumulate with age in somatic cells. If so, an age-dependent increase in the frequency of such PRDM9-independent NCOs in sperm should be shown and analyzed explicitly.

We agree age-dependence of the PRDM9-independent NCOs in sperm is an interesting prediction; however, with our small, uneven cohort (12 individuals/twins) and the minority size of the PRDM9-independent class, the power to detect a modest slope is very limited. Furthermore, a mitotic-like origin does not require a linear age trend in sperm or even one that is consistent across individuals, given spermatogonial dynamics and potential environmental causative factors.

Somatic NCOs:
Similar concerns apply to putative NCOs detected in somatic cells. There is a modest enrichment of these events in fragile sites (26% vs. 22% background), potentially suggesting an association with somatic DNA breaks. However, the analysis is incomplete, and it remains unclear whether the observed SNV alterations (termed NCOs in the manuscript, though "gene conversions" would be more accurate, as NCO refers to the process) are instead artefacts arising from sequencing errors, contamination, or DNA repair mechanisms unrelated to homologous recombination.

We respectfully disagree that the somatic events that we observe can be dismissed as artefacts. First, the enrichment at fragile sites, though modest, is reproducible and biologically meaningful, as random false positives from contamination, assembly issues, or sequencing errors would be expected to distribute uniformly across the genome. Second, our analysis already incorporates stringent contamination controls, including per-read and per-site base quality calibration, filtering of reads inconsistent with both haplotypes, and removal of contaminating reads. Any residual contamination would not selectively enrich events at fragile sites. Third, these somatic events share key features with the PRDM9-independent sperm NCOs, attenuated GC-bias, and fragile-site enrichment, which is difficult to reconcile with random technical artefacts and instead points to a shared underlying biology. Finally, while we agree that the term "NCO" strictly refers to outcomes of homologous recombination and we do not demonstrate that they arise from homologous recombination-mediated double strand break repair (although that is a consistent and plausible origin), our findings remain important regardless of the precise pathway involved. The ability to detect gene conversion-like events in somatic cells at genome-wide scale provides a new view of processes that can cause loss of heterozygosity with important implications in cancer and aging.

It is therefore essential to present data on the frequency and distribution of (i) small deletions, (ii) insertions, and (iii) mismatches to the haplotypes—since none of these can arise from NCOs. Their prevalence would help assess whether the observed SNV alterations truly result from gene conversions during NCO-type recombination in somatic cells.

We agree that the prevalence of mismatches to both haplotypes is important to examine, since these would reflect either new mutations or errors, either of which when occurring at a pre-existing heterozygous site could emulate gene conversion events and hence generate false positive gene conversions. We had already explicitly done this – our choice of filters was to ensure that the error level was sufficiently low that the vast majority of such events are somatic mutations, which themselves are at too low a level to provide a major contribution to the observed gene conversions.

With respect to small insertions and deletions, we recognise that even PacBio CCS reads have a significant rate of small indel errors. However, since we are considering gene conversion at single nucleotide heterozygous positions and removing all reads that introduce indels in the vicinity of the site, there would need to be two indel sequencing errors that cancelled each other in offset while introducing the correct single nucleotide variant and no other sequence change in order for indels to cause a false positive. Furthermore, were this to happen it would be indistinguishable from a single nucleotide substitution error, so the rate of these is already included in the single nucleotide error control.

Moreover, an analysis of GC-bias in the putative somatic gene conversions and a detailed analysis of their genome-wide distribution would be informative.

We have calculated the GC-bias metric for the putative somatic gene conversions and will include it in the revision. The results (46%) are in clear contrast to the strong positive (greater than 50%) GC-bias as seen for the PRDM9-associated GCs. Given the modest departure from 0.5 and current sample size, we do not believe the slight negative deviation from neutrality is biologically significant.

The manuscript rightly states that most somatic recombination takes place between sister chromatids, rather than homologous chromosomes. This widely accepted knowledge is invoked to provide an explanation for the abundant (putative) NCOs but rare COs in somatic cells. However, this interpretation is incorrect: recombination between sister chromatids neither generate observable NCOs (gene conversions) nor observable COs because sister sequences are identical preventing the detection of recombination outcomes by sequencing. Both observable COs and observable NCOs only result from homologous recombination between homologs, which is considered rare in somatic cells. Therefore, the data must convincingly exclude alternative repair processes that could yield repair products erroneously identified as NCOs. This is essential before drawing any conclusions regarding somatic NCO recombination.

We agree with the reviewer that recombination between identical sister chromatids cannot be detected by our sequencing approach, as such events produce no observable sequence differences. We did not intend to suggest otherwise. Our observations therefore directly indicate that the putative somatic gene conversions must arise from recombination between homologous chromosomes or from other templated repair processes capable of generating detectable conversion tracts. We now make this explicit in the revised Discussion. The enrichment of these

events at fragile sites, their shared positional and sequence characteristics with the "mitotic-like" sperm NCOs, and the extensive filtering we apply to remove potential contamination or assembly artefacts, all argue that these are genuine repair products rather than noise. We agree that the exact repair pathway remains to be established, but feel it is reasonable to suggest that they may reflect non-canonical homologous recombination or alternative templated repair mechanisms. In our view, our lack of a full explanation of the mechanisms underlying our observations does not weaken the finding. If everything had to be fully explained when first published in Nature then many important papers with important new insights would not have been published there.

Variation in Recombination Sites:
The manuscript emphasizes variation in CO and NCO properties between individuals with the same PRDM9 alleles, between repeated samples from the same individual at different ages, and between monozygotic siblings. In my initial review, I noted that the biological significance of these observations was unclear, and that some may simply reflect stochastic sampling of potential recombination sites. I recommended performing replicate sequencing on split biological samples to test the contribution of sampling noise and to evaluate the method's reproducibility.
The authors addressed this by including additional sequencing of some sperm samples and pooling old and new sequencing data from each sample. However, instead of pooling old and new datasets, the manuscript should present the new data side-by-side with the original data as technical replicates to allow the reader to assess variability arising from sampling or technical factors. Cross-referencing the newly generated pooled data (Fig. 3) with the original dataset suggests considerable variation between technical replicates. For instance, in the current data, NCOs in the AA2-t2 sample overlap PRDM9-DSB hotspots significantly more than in the AA2-t1 sample, whereas the previous data showed the opposite trend. A significant shift is also observed in the NCO characteristics in the AA4 sample following deeper sequencing. These discrepancies suggest that much of the reported inter-individual variation may stem from sampling effects or technical variation rather than true biological differences. This should be clearly acknowledged and discussed to explicitly warn readers that the observed variations have questionable biological meaning and significance.

We agree that assessing the reproducibility of observed inter-individual differences in recombination site usage is important, and we appreciate the reviewer's suggestion to evaluate sampling noise and technical variability in this fashion. We would like to highlight that our study already incorporates an implicit form of replication because the original and new TwinsUK datasets were generated independently, at different times, and from distinct sequencing runs using the same donor material. Therefore, any consistent patterns across the two TwinsUK datasets are unlikely to be artefacts of a single experimental batch. Additionally, we note that our samples already are a combination of two datasets (TwinsUK and SL) which were sequenced and processed independently at two different centres. Therefore, qualitative results such as discrepancies in fraction of reads overlapping PRDM9 motifs, also replicate across these two datasets.

In our revision, we opted to pool the datasets per donor before final analyses in order to maximise our detection power, particularly for rarer NCO subclasses, and to reduce statistical noise inherent to small sample sizes. Here are the requested explicit visualisations of the two datasets side-by-side as technical replicates. First batch (Sequel II):

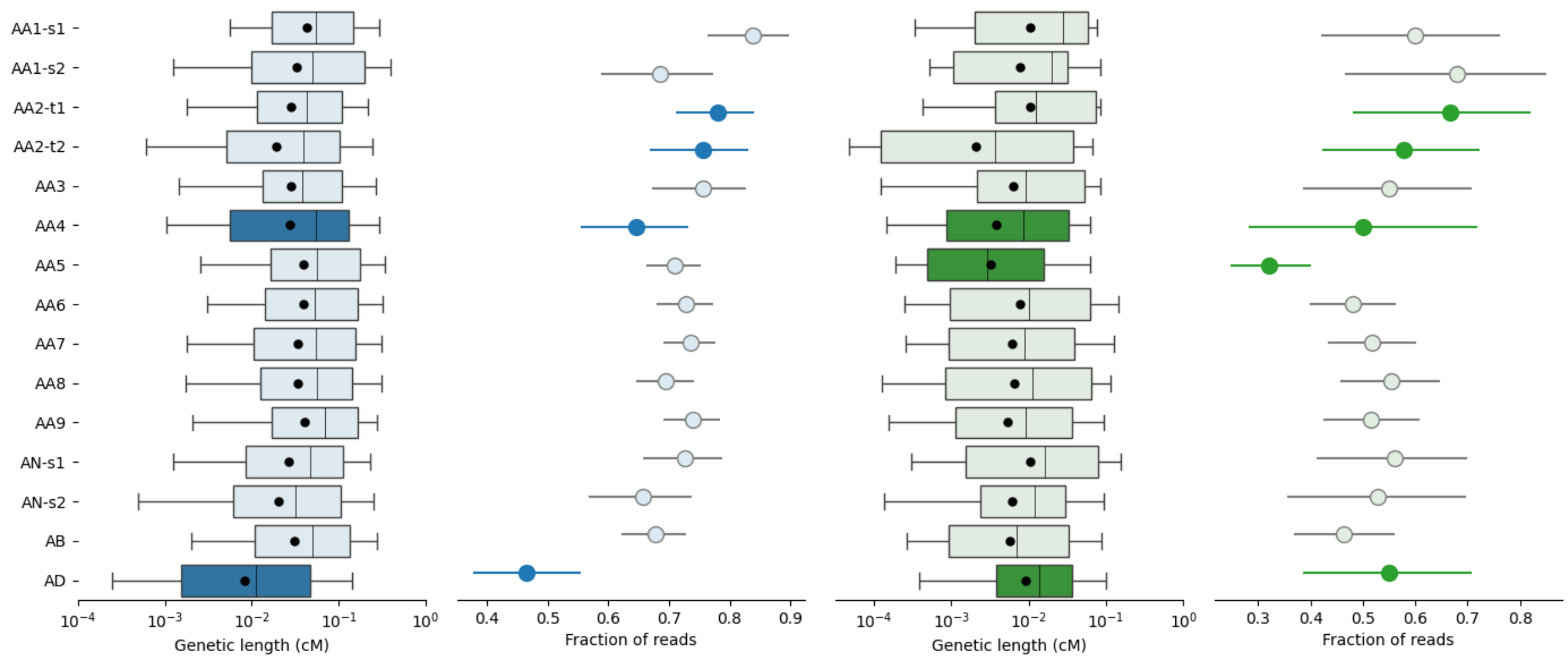

Second batch (Revio):

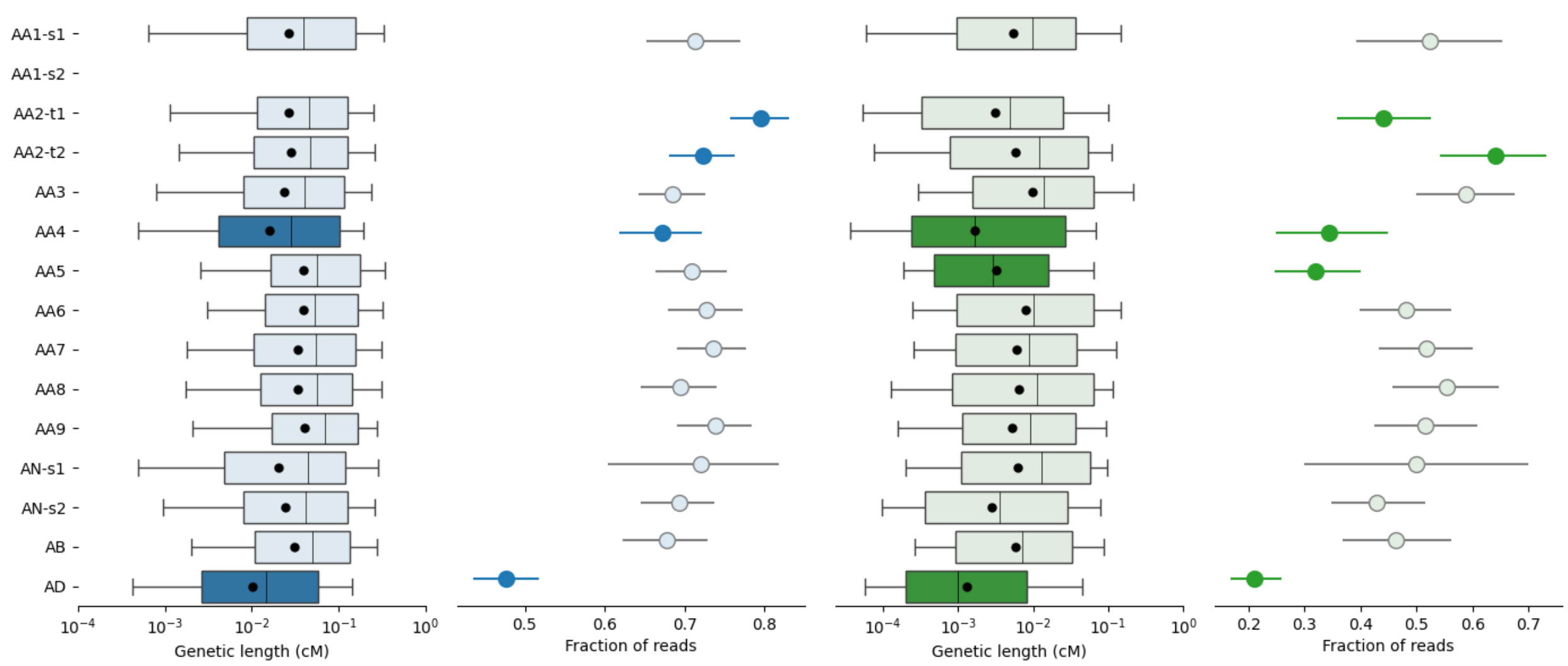

This is what we have in the paper:

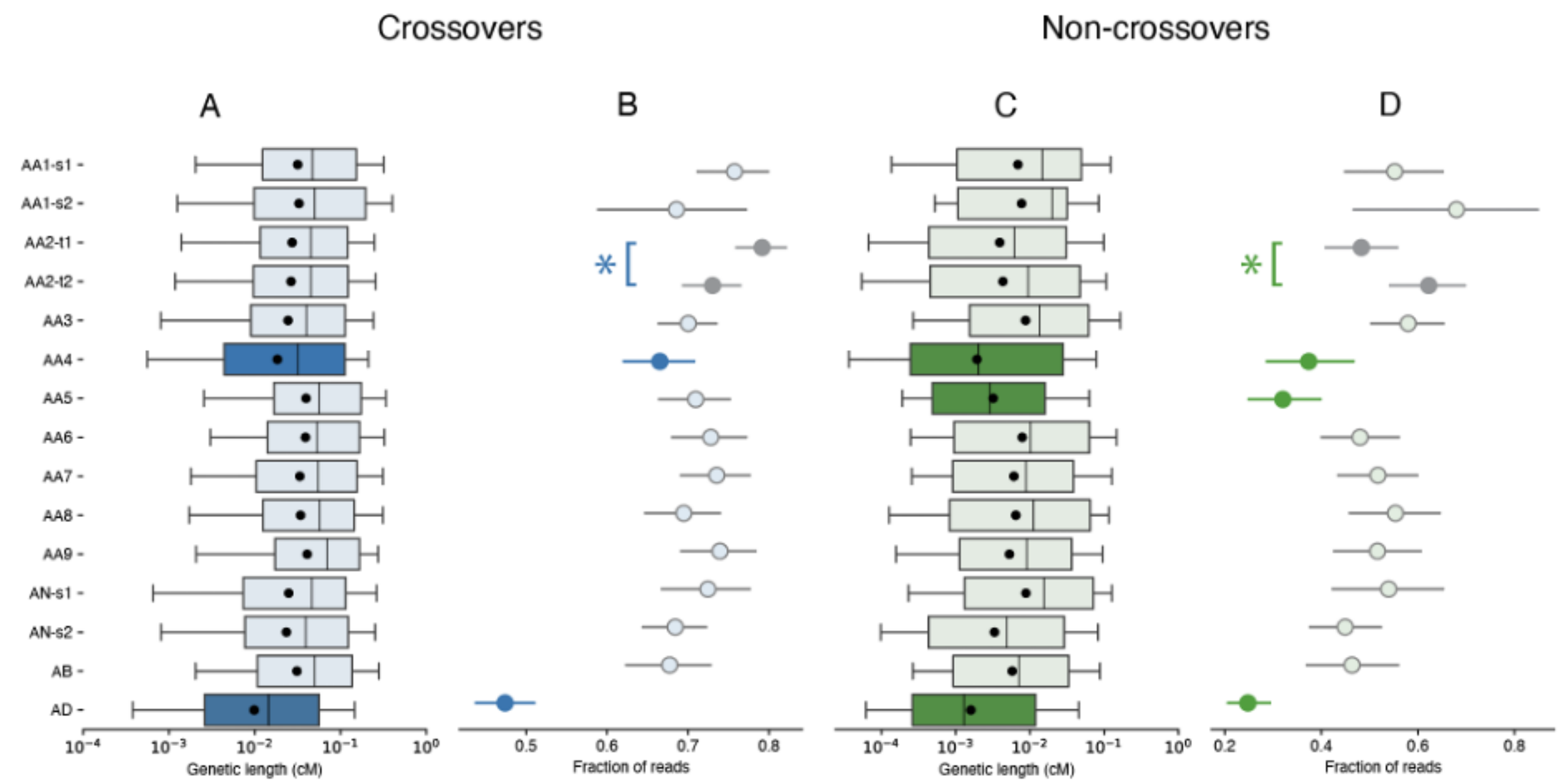

(SL samples do not change between plots since they were not re-sequenced)

When we perform such side-by-side comparisons, we find that although some quantitative metrics (e.g., precise enrichment percentages at PRDM9 hotspots) fluctuate between batches, the qualitative features of inter-individual variation remain consistent. For example: the most and least active hotspot regions per donor remain the same across batches; differences in hotspot usage between monozygotic twins are identified in both batches with similar effect directions; inter-individual variation in NCO tract length distribution and GC-bias is qualitatively consistent. We also note that some of the differences between the revisions in marking a result as statistically significant are not due to experimental stochasticity, but due to improved processing pipeline, better contamination controls, and a more stringent and streamlined threshold for significance, as required by the reviewers in their first review.

As this reviewer will appreciate the batch-to-batch differences in precise overall percentages are expected given that relatively small number of recombination events per individual in each replicate, and the inherent stochasticity of recombination site sampling. Importantly, the magnitude of variation observed between individuals with identical PRDM9 alleles exceeds the variation observed between replicates of the same individual, suggesting that at least part of the signal reflects true biological differences rather than technical or sampling noise.

We have adjusted the text to acknowledge that stochastic sampling and technical variation may contribute to some of the observed variability, but we believe the persistence of inter-individual differences across independent datasets and repeated sampling supports the conclusion.

Minor Comments:

• When referring to enrichment of COs or NCOs in various genomic regions (e.g., PRDM9 sites), it is unclear what is meant by “all reads.” Please clarify whether this refers to all recombinant reads, all candidate recombinant reads, or all high-quality SNV-containing reads.

We mean by “all reads” the “196,296,004 reads that map unambiguously to both haplotypes and satisfy other quality control filters (**Methods**)” (lines 140,141) – we now define this at that point in the text.

• There may be a typo in the sentence: “In contrast to both COs and NCOs, only 21% of complex event reads overlap a DSB-PRDM9 motif (not significantly different from the background 25%).” Based on an earlier statement—“We found that 67% of CO reads and 45% of NCO reads overlapped a DSB-PRDM9 motif (Fisher’s exact test, P<1e-10), compared with 13.5% of all reads”—should “background 25%” instead read “background 13.5%”?

The typo was in the text not the number. The sentence in question should read “In contrast to both COs and NCOs, only 21% of complex event reads contain a PRDM9 motif (MEME motif finder, not significantly different from background 25%)”. We have made that change. For this analysis we just looked at whether any PRDM9 motif was present, rather than the earlier analysis in which we looked for overlap with a DSB-PRDM9 motif. We apologise for the error in the statement.

Schweiger et al. “Long-read sperm sequencing reveals a pre-meiotic component in non-crossover recombination” response to referees

We thank the referees and in particular the new fourth referee for their comments. We are pleased to resubmit a final version in which we believe we have addressed all the issues that were raised. In particular, as outlined at the start of our response to referee #4, we have added analyses of two new orthogonal data sets, which both support our conclusions.

Below we give the referees’ comments in black and our responses in blue.

Referee #2 (Remarks to the Author):

After reading the revised manuscript and the detailed rebuttal, my view is that the authors have addressed the core technical concerns raised earlier, including many of Reviewer #3’s points regarding contamination, sequencing artefacts, and sampling noise. The expanded analyses, in particular the quantification of haplotype-inconsistent bases, the fragile-site enrichments, the GC-bias stratification, and the side-by-side comparison of replicate sequencing batches, strengthen the evidence that a large fraction of the identified non-crossover events represent genuine biology rather than technical noise.

The dataset now makes a strong case for a significant PRDM9-independent class of sperm NCOs with properties that differ from canonical meiotic recombination. This class shows:
(1) attenuated or absent GC-bias
(2) clear enrichment at fragile genomic regions
(3) lack of association with PRDM9-directed DSB maps
(4) close similarity to gene-conversion–like events observed in matched somatic tissue.
These signals appear consistently across individuals and across independently generated sequencing batches, and they sharpen under stricter filtering. For these reasons, I view the existence of a substantial non-meiotic component of sperm NCOs as well supported.

We thank the referee for acknowledging our contribution and the robustness of our findings. We note that our further data analyses performed for this revision, and in particular of germline cells from testis biopsies (see below) provides further strong support for this claim.

The authors interpret this PRDM9-independent class as arising from pre-meiotic, mitotic-like templated repair, and this is a plausible and well-reasoned explanation. However, I would emphasize, as the authors themselves now state more explicitly, that the assignment to a specific repair pathway or developmental stage is inferential. The data show that these events are not typical meiotic NCOs, but they cannot by themselves distinguish among several possible non-meiotic mechanisms. Therefore, while the evidence for a genuine non-meiotic class is

strong, in my view the mechanistic conclusion should be framed as consistent with, rather than definitively demonstrating, mitotic homologous recombination in germline precursors.

We agree with the referee that our data is not definitive about the mechanistic origin of these mitotic-like NCOs. We have modified key sentences in the Summary and Discussion.

Referee #4 (Remarks to the Author):

We thank the referee for their careful and thorough reading of our manuscript, and for the detailed and constructive comments. We greatly appreciate the time and effort invested in evaluating the work, which we believe now have substantially strengthened the manuscript. We have responded point-by-point to every comment raised below. Beyond clarifications and revisions to the existing analyses, the main substantive change is the inclusion of two additional, orthogonal datasets that allow us to test our central claims under independent experimental and technical conditions.

First, we reanalysed the deCODE high-coverage pedigree sequencing dataset from Palsson et al. Nature 2025. This dataset is orthogonal in both experimental design and sequencing technology, relying on short-read, high-coverage whole-genome sequencing that identifies events consistently inherited across pedigrees rather than single-molecule long-read sequencing. As such, it is not subject to the potential technical artefacts specific to PacBio HiFi reads nor to potential issues in our pipeline. Strikingly, the paternal non-crossovers in the deCODE data recapitulate the same molecular signatures we observe in our PacBio sperm data, including reduced hotspot association and GC-bias patterns. In contrast, maternal non-crossovers display markedly different properties. This natural separation between paternal and maternal germlines provides an internal control: only the paternal events show the pre-meiotic-like signature, consistent with the biology of ongoing mitotic divisions in the male germline. Together, these independent analyses substantially strengthen the evidence for a genuine pre-meiotic component of human sperm non-crossover recombination.

Second, we analysed additional PacBio HiFi long-read data from the recent Porsborg et al. Nat Comm 2025 study, which includes both human sperm samples and, crucially, human testis biopsies. The testis biopsy data provide direct access to germline cells at pre-meiotic stages, matching the cellular context we propose as the source of the non-meiotic component. Importantly, in the testis germline read set we observe clear evidence of an enrichment of non-crossover events with the same properties identified in our manuscript as pre-meiotic. The presence of an enrichment of such events in germline cells, reflecting their pre-meiotic component, provides direct biological support for our interpretation and reduces reliance on inference from sperm and inherited events.

In this manuscript the authors use Pacbio HiFi long read sequencing to identify non-crossover DSB repair products in sperm and characterize their features. The major claim is that a large fraction of NCOs appears to originate from DSBs arising during mitotic growth rather than from meiotic DSBs. The authors also find variation in recombination events between donors, including donors who share the same PRDM9 alleles, as well as between identical twins.

The presence of "mitotic" NCOs in sperm is not surprising in itself, but their frequency (30-40% of all observable NCOs) appears high. In non-meiotic cells, the majority of DSBs are repaired by non-homologous end joining, and the vast majority of those repaired by homologous recombination use the sister chromatid. Consequently, observable somatic recombination events are extremely rare. Unfortunately, the authors do not comment on this fact and do not

attempt to estimate whether the high number of putative "mitotic" NCOs in sperm is even theoretically achievable through somatic inter-homologue recombinational repair. Without appropriate calculations and/or simulations, it is difficult to assess whether the "mitotic" NCOs identified in this study could indeed arise pre-meiotically. The fact that the authors observe NCOs with similar characteristics in blood samples could indicate that "mitotic" NCOs are indeed what they propose. However, it could just as well suggest that such NCOs represent a common artifact or a misinterpretation.

We thank the referee for highlighting the need for a quantitative assessment of whether the observed fraction of putative pre-meiotic NCOs is compatible with known properties of mitotic double-strand break repair. In response, we provide here an explicit calculation, based on published estimates of germline mitotic divisions, DSB rates, and inter-homologue repair frequencies, to evaluate whether the observed numbers are theoretically achievable.

First, a review of the origins and patterns of human mutation by Crow (*Nat Rev Genet*, 2000) estimates approximately 30 mitotic cell divisions in the male germline by age 15, followed by roughly 23 additional divisions per year thereafter, corresponding to approximately one stem-cell division every 16 days. Given that the mean age of donors in our dataset is ~42 years, this implies an expected value in the range of ~600–700 divisions. (Across the observed donor age span of 26 to 74 years, the corresponding number of pre-meiotic divisions ranges more broadly from roughly 300 to 1,400.)

A review by Bouwman and Crosetto (*Genes*, 2018) states that “the frequency of DSBs is estimated to be 10–50 events per cell per day.” Similarly, Vilenchik and Knudson (*PNAS,* 2003) write: “We estimate that in normal human cells ≈1% of single-strand lesions are converted to ≈50 endogenous DSBs per cell per cell cycle.” Although the precise number depends on cell type and state, these estimates consistently indicate the same order of magnitude. A conservative working range of 10–50 DSBs per cell cycle is therefore supported.

Stinson and Loparo (*Annu Rev Biochem*, 2021) state that “NHEJ is the predominant pathway in human cells and repairs up to ~80% of all DSBs,” and Shibata et al. (*EMBO J*, 2011) report that “NHEJ represents the major DSB repair pathway in G2 as in G1 with ~75–85% of IR-induced DSBs undergoing repair by NHEJ.” These place NHEJ in the ~75–85% range in mitotic human cells, with homologous recombination accounts for the remaining ~15–25% of DSB repair events. A working estimate of 15–25% HR is therefore supported.

Moynahan and Jasin (*Nat Rev Mol Cell Biol*, 2010) note the “low overall frequency of inter-homologue HR (1 in 100 HR events)” in mitotic cells, indicating that only ~1% of homologous

recombination events use the homologous chromosome as a repair template rather than the sister chromatid.

It is well established (e.g. reviewed in Sung, P., Klein, H. *Nat Rev Mol Cell Biol*, 2006) that in mitotically dividing cells, HR overwhelmingly resolves DSBs as non-crossover gene conversion. This strong bias reflects active crossover-suppression mechanisms during mitotic repair, including dissolution of recombination intermediates by the BLM helicase complex. Consistent with this, our blood-based analyses similarly detect nearly zero crossover events, supporting the conclusion that >99% of mitotic recombination outcomes are non-crossover gene conversions.

Finally, based on our own calculations (as described in the manuscript) and explicitly accounting for the short tract lengths and marker density, we estimate that only ~1–2% of gene conversions would convert at least one SNP and be detectable.

Combining these ranges yields a straightforward quantitative bound: For a mean donor age of ~42 years, we expect ~600–700 pre-meiotic mitotic divisions per lineage (broader range ~300–1,400). Assuming 10–50 DSBs per division gives ~6,000–35,000 total DSBs per lineage (up to ~70,000 at the extreme). With ~15–25% repaired by HR, this corresponds to ~900–8,750 HR events, of which ~1% involve the homologous chromosome, yielding ~9–88 inter-homologue gene conversions per lineage. Accounting for our estimated 1–2% detectability given short tract lengths and marker density, this translates to an expected ~0.1–1.8 detectable inter-homologue GCs per sperm lineage, with central parameter choices near ~0.5–1 event. In our data, at a total coverage of 1173.1× we observe 2,382 non-crossovers; if ~30% of these are pre-meiotic (“mitotic”), this corresponds to ~0.6 detected mitotic non-crossovers per haploid genome, well within the predicted range and definitely within the correct order of magnitude.

We further note that Jia et al. (PLOS Biology, 2021) make a closely related quantitative argument based on large-scale empirical data in plants. By examining ~1.1 million rice plants, they directly estimated the per-mitosis rate of mitotic gene conversion and found it to be roughly two orders of magnitude lower than the meiotic rate. However, they then explicitly modeled the cumulative number of gene conversion tracts expected between zygote and gamete, incorporating both the number of pre-meiotic mitoses and tract length distributions. They showed that, despite the low per-division rate, the large number of mitotic divisions and relatively long mitotic tract lengths can result in a total number of converted markers comparable to that generated by meiosis. Their analysis demonstrates that even rare mitotic events can accumulate to non-negligible levels over many cell divisions, directly paralleling the quantitative framework applied here.

We added a sentence about this calculation in the main text, with the calculation itself in the supplement, and also reference the rice paper in the results section.

My other concern is the lack of GC bias in the conversion tracks of the “mitotic” NCOs. The authors use this as one of the criteria that supports their non-meiotic origin, stating that gBGC is specific to meiosis. As far as I know, GC bias is observed regardless of whether repair occurs in

meiotic or somatic tissues. If the authors have evidence to the contrary, they need to provide appropriate citations.

We agree that GC bias is a well-established feature of meiotic gene conversion in humans. However, the expectation that all inter-homologue gene conversions, regardless of context, must exhibit GC bias is not supported by the broader literature. First, mitotic inter-homologue gene conversions in humans are rare and so far have been technically difficult to detect, so there is in fact very limited direct evidence regarding their allele transmission bias. Second, even within meiosis, GC bias is not universal across repair contexts. Li et al. (*Nat Commun*, 2019) showed in mouse that short NCO tracts display strong GC bias whereas longer NCO tracts do not, demonstrating that different repair scenarios can produce gene conversion without gBGC. Similarly, in budding yeast, Lesecque et al. (*Mol Biol Evol*, 2013) showed that gene conversion tract properties are inconsistent with a simple, universally GC-biased mechanism. More broadly, Liu et al. (*Nat Ecol Evol*, 2018) reported no detectable gBGC in gene conversion tracts across multiple plants and fungi. Taken together, these results indicate that gBGC depends on the specific molecular context of repair and cannot be assumed to accompany all gene conversion events.

More importantly, the gBGC pattern is independently recapitulated in the deCODE pedigree dataset (Palsson et al., *Nature* 2025). Stratifying paternal NCOs by local genetic map rate (cM/Mb) at converted SNPs in detected GCs, we observe that GC bias is high in regions of elevated crossover recombination rate and declines progressively as the local cM/Mb decreases, approaching ~50% in the lowest-rate bins (see new Fig. 2). In contrast, maternal NCOs show no such dependence on cM/Mb: their GC bias remains stable across the full range of recombination rates. Thus, attenuation of GC bias with decreasing map rate is specific to the paternal lineage. If low-map paternal NCOs were technical artefacts, there is no reason why the same analysis framework would yield this pattern only in fathers but not in mothers. This supports the interpretation that a subset of paternal NCOs arises from a PRDM9-independent, pre-meiotic process lacking detectable gBGC.

Because of this concern we consulted Laurent Duret, an expert on gBGC and author of a major review (*Annu Rev Genomics Hum Genet*, 2009). His position was that the underlying mechanism is still unclear (e.g. whether BER or MMR is the source) and we don’t know which processes contribute in which contexts, so our observations are not inherently unexpected.

We changed our statement in the results where we motivate looking at gBGC and referenced the relevant papers.

Another criterion is that observed NCOs overlap PRDM9 binding motifs, known DSB sites or the sites of COs less frequently than observed COs do. Although this could indicate that a fraction of NCOs is of non-meiotic origin, this could also mean that these are misidentified NCOs, consistent with the fact that NCOs are much more difficult to detect.

Our analyses of the deCODE data and Porsborg data suggest this is not the case. For deCODE, the detection method is entirely unrelated, and for Porsborg, the increase in the fraction when looking at germline testis reads which are enriched for pre-meiotic events is consistent with the non-overlapping events being real and pre-meiotic.

The correlation with fragile sites is rather small (a 5% increase over a 21% background). It would be interesting to know whether “mitotic” NCOs share some other genomic features – e.g., do they show any correlation with promoters, enhancers, CGIs, etc.?

We agree that this association is small, although we now confirm it in the deCODE data. We should not expect that all double-strand breaks occur at fragile sites, so the quantitatively low, although significant, level of association is not surprising. Following the reviewer’s comments, we did explore some of the suggested features, and others such as replication timing, but none had substantial increases (or decreases) and any weak signals that exist could not be disentangled from correlations between annotation types.

As for the other main findings, inter-individual variation in recombination hotspots among individuals with identical PRDM9 alleles has been previously reported.

We have only been able to find one study that directly reports inter-individual variation in hotspot activity among individuals carrying identical PRDM9 alleles, namely Pratto et al. *Science* 2017. That demonstrated that a small fraction (~5%) of $PRDM9_A$-defined hotspots vary in strength between individuals, but was based on DSB maps from a limited number of donors (two with $PRDM9_A$) and focused on hotspot initiation rather than on recombination outcomes. In contrast, our study quantifies inter-individual variability in crossover and non-crossover recombination products. So to our knowledge, our work constitutes the first genome-wide characterization of inter-individual variation in recombination landscapes among individuals with identical PRDM9 alleles at the level of resolved recombination events. Previously Jeffreys et al. had reported differences in crossover recombination rate between individuals at a specific locus, but those individuals were not genotyped for PRDM9. Nevertheless, we have removed our claims of “previously unreported” in the introduction and “first” in the discussion for this finding. In any case, if we have missed something here and a reference can be provided we would be happy to include it.

The authors show the variation in additional recombination features, but they are looking at only ~180 NCO events per individual, whereas there are ~40K hotspots in humans. These hotspots vary in strength and genomic context, and the observed inter-individual differences in NCO features may simply result from sampling different subsets of hotspots in different individuals.

We appreciate the reviewer’s point that individual genomes contain on the order of ~40,000 recombination hotspots, which vary in strength and genomic context. However, our analysis does not attempt to estimate hotspot-specific properties across all loci. Rather, for each individual we analyse approximately ~180 independent NCO events and compare the distribution of *their properties* across individuals. Under the null hypothesis that individuals with identical PRDM9

alleles share the same underlying distribution of NCO properties, each observed NCO represents a draw from that common distribution. Sampling different subsets of hotspots therefore generates ordinary sampling variance, and this variance is explicitly accounted for by our two-sample distribution tests and permutation procedures. If the observed inter-individual differences were solely due to chance sampling of different hotspots, they would be captured by this variance. The fact that we observe statistically significant and reproducible shifts in these distributions beyond sampling variance, including between monozygotic twins, indicates that the differences exceed what would be expected from hotspot sampling alone.

The authors may ultimately be correct that a substantial fraction of NCOs are indeed produced pre-meiotically. However, the data presented and/or perhaps insufficient discussion leave some doubts regarding the validity of this conclusion. It would be very helpful if the authors could examine NCOs identified by a different approach to determine whether the "mitotic" NCOs can also be detected in that data.

As we described above, we have now analysed two additional datasets: the deCODE data which detects NCOs using a different approach and technology, and long-read PacBio HiFi sequencing from sperm samples and testis biopsies. Both these, and further analyses we present based on them, replicate and confirm our findings.

Specific comments:

Abstract, line 40: "... we see non-crossover gene conversions with similar properties, increasing with age" - this is misleading, because, according to the main text, this correlation does not reach statistical significance.

We removed this from the abstract, and added the deCODE confirmation.

Line 65: "... the presence of events weakly linked to PRDM9 hotspots" - it is not clear what the authors mean by that, and the reference is missing

We rewrote this and provided a reference.

Line 97: "... single sperm..." this could be interpreted as "single cell", suggest rephrasing

We changed the text from "single sperm sample" to "deep sequencing of a bulk sperm sample from a single individual".

Line 101: "... previously unreported variation between individuals in patterns of recombination..." - this is an overstatement. E.g., hotspot variation in individuals carrying the same PRDM9 alleles has been previously reported.

We removed this, as discussed above.

Line 150. SNVs within tandem repeats have been removed. How does this affect reads within fragile sites?

The fragile site annotation that we use extends well beyond tandem repeats (many regions in the megabases). We will indeed have lower power to detect recombination events, in particular short NCOs, in regions dense in tandem repeats. But this would only weaken, not strengthen the enrichment that we see.

Line 171: "... we detected 11 COs, 369 NCOs..." (in blood samples). The authors detected 7,143 COs and 2,382 NCOs in sperm samples. How do they explain that the fraction of NCOs (compared to COs) in blood is so much higher than in sperm? If I remember this correctly, around 8% of DSBs repaired through homologous recombination in somatic tissues result in COs rather than NCOs - a similar ratio (5-10%) as in meiosis. I did not quite understand the explanation provided in lines 173-175.

It is well established that the fraction of DSBs resolved as crossovers by HR is much lower in somatic tissue than in meiosis, e.g. reviewed in Sung, P., Klein, H. (*Nat Rev Mol Cell Biol*, 2006). This is the consequence of the Bloom's complex, loss of function in which leads to reappearance of somatic homologous crossover and large scale loss of homozygosity. We changed the confusing sentence in lines 173-175, which wasn't providing the right explanation.

Lines 184-186. I found this sentence confusing, and I did not find the description in the methods section.

We simplified the sentence in the results, and added a description in the methods.

Fig. 2B and lines 197-198. I have some difficulty understanding what is what here; there are three color shades in the legend, but five on the figure. I realize that some color shades are created by overlap, but I can't figure out which overlaps produce which colors. It doesn't make it easier that the legend refers to grey color reads as "all reads", but the main text refers to them as "all remaining reads" – i.e., those that are left after CO and NCO reads are removed. I basically have to believe the authors' word that the distribution of NCO genetic length is bimodal.

We changed these plots from shaded regions to lines.

Line 208 and citation 37. In the cited work, somatic gene conversion was investigated in cancer cells and could hardly be applied to regular tissues. It's got to be a better citation.

We replaced this citation with a review which describes gene conversion in a mitotic DNA repair context (Moynahan, M. E. & Jasin, M.. *Nat. Rev. Mol. Cell* (2010)).

Line 211, citation 38. There is nothing about NCO enrichment at fragile sites in Blooms in the cited paper.

We removed this statement and reference.

Lines 211-213: "Finally, they may lack the GC-biased allele transmission that is a hallmark of meiotic mismatch repair-mediated gene conversion". This is oddly put, because it sounds as if gBGC only occurs during meiosis. As far as I know, GC bias is present in both meiotic and somatic tissues, but because somatic DSBs are mostly randomly distributed, it is meiotic DSBs that create observable gBGC signatures (evolutionary changes in genomic sequence) at recombination hotspots.

We have changed this text, and discuss further above.

Line 233: "previously published meiotic DSB map of 28,286 human recombination hotspots" – one of the listed references reports more than 34K DSB hotspots for an AA individual, what was the reason not to use all of them?

The original Pratto et al. *Science* 2014 contains the DMC1 data from which 34K DSB hotspots were indeed called for an AA individual. However the later Hinch et al. *Science* 2023 re-analyses the same data with a custom pipeline which was specifically designed for DMC1 data, which has higher sensitivity and specificity, and only calls 28,286 hotspots. We chose to use the latter.

Line 270 and the paragraph below, GC-bias in NCO tracts. The fact that "somatic" NCOs in sperm and blood do not show GC bias may suggest that these are erroneous NCO calls. This will also explain why they are not associated with PRDM9 motifs.

We address this above, and now more thoroughly in the manuscript.
